# Supplementary material for: Sustained SREBP-1-dependent lipogenesis as a key mediator of resistance to BRAF-targeted therapy
Source: Nat Commun. 2018 Jun 27;9:2500. doi: 10.1038/s41467-018-04664-0 (PMC6021375; doi:10.1038/s41467-018-04664-0)
Supplement: Supplementary file 1 — Supplementary Information [file 41467_2018_4664_MOESM1_ESM.pdf]

Sustained SREBP-1-dependent lipogenesis as a key mediator of resistance to BRAF-targeted therapy

Talebi et al.

Supplementary Information

Supplementary Table 1 Overview of the cell line panel including driver mutations and the mechanisms of drug resistance if applicable.

Cell Line	cell type
451lu	Human BRAF^{V600E} MM
451lu R	Vemurafenib resistance Human BRAF^{V600E} MM clone
mode of resistance:	*RAF kinase flexibility and enhanced IGF-1R signalling*
M229	Human BRAF^{V600E} MM
M229 R	Vemurafenib resistance Human BRAF^{V600E} MM clone
mode of resistance:	*Enhanced RTK signalling*
M238	Human BRAF^{V600E} MM
M238 R	Vemurafenib resistance Human BRAF^{V600E} MM clone
mode of resistance:	*Enhanced RTK signalling*
M249	Human BRAF^{V600E} MM
M249 R	Vemurafenib resistance Human BRAF^{V600E} MM clone
mode of resistance:	*Acquired NRAS^{Q61K} mutation*
A375	Human BRAF^{V600E} MM
FLCM	Mouse BRAF^{V600E} MM
M202	Human BRAF WT NRAS mutant MM
M207	Human BRAF WT NRAS mutant MM
M257	Human NRAS and BRAF WT MM
NHEM	Normal neonatal human epidermal melanocyte pool from three donors

a

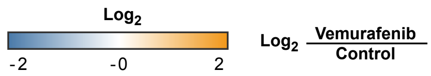

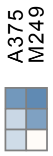

Annotation	Log$_2$ FC A375	Log$_2$ FC M249	P-value A375	P-value M249	FDR A375	FDR M249
FASN	-1.27	-1.86	1.97E-65	1.63E-135	2.58E-64	3.21E-133
ACLY	-0.42	-1.97	1.68E-13	1.32E-255	5.60E-13	5.87E-253
ACACA	-0.13	0.12	0.01	0.02	0.02	0.05

b

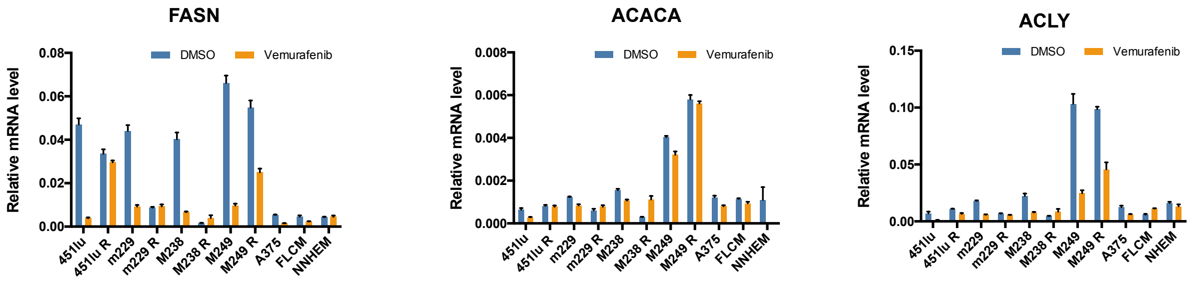

Supplementary Figure 1 (a) RNAseq of A375 and M249 cells treated with Vemurafenib (5 µM). (b) The full cell line panel was subjected to RT-qPCR for FASN, ACACA and ACLY after Vemurafenib treatment, data are represented as mean ± s.e.m.

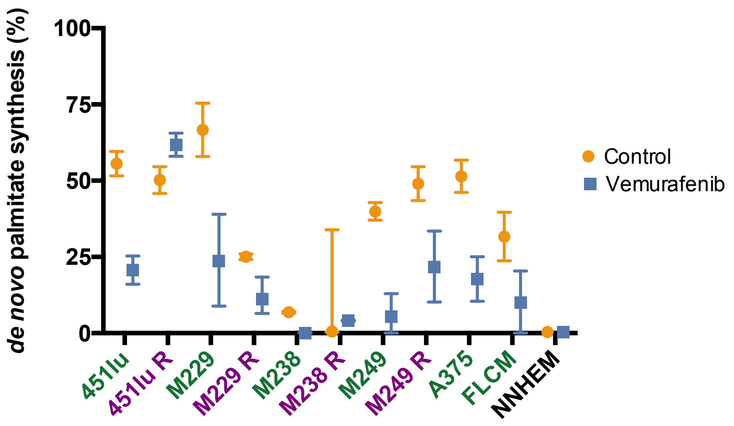

Supplementary Figure 2 Fraction of *de novo* synthesized palmitate was measured based on isotopomer spectral analysis using $^{13}C_6$ glucose (4.5 g L^{-1}) incorporation into palmitate upon Vemurafenib (5 µM) treatment. *De novo* palmitate synthesis in NHEM was below the detection threshold. All bars represent 95% confidence intervals.

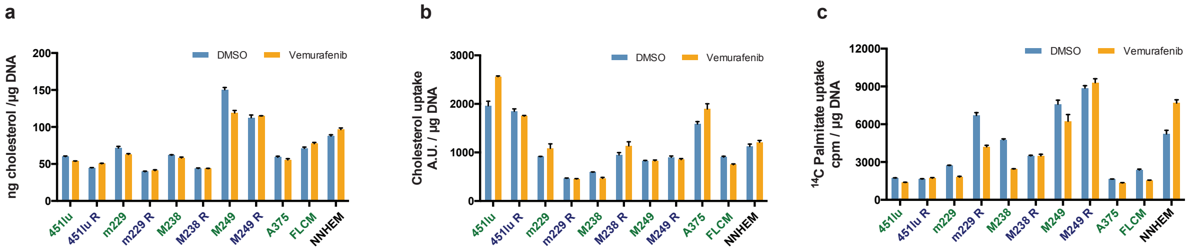

Supplementary Figure 3 (a) Cholesterol quantification using the Amplex Red cholesterol assay kit (b) Cholesterol uptake was quantified by adding NBD cholesterol to the culture medium and normalizing the fluorescent signal from collected cells to DNA content. (c) ^{14}C palmitate uptake was measured after 4 hours of incubation by counting radioactivity in collected cells. All data are represented as mean ± s.e.m.

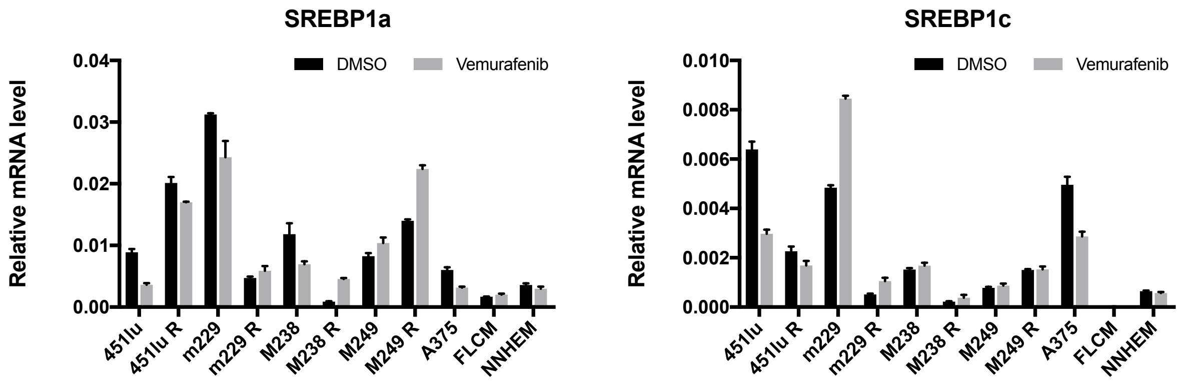

Supplementary Figure 4 The full cell line panel was subjected to RT-qPCR for SREBP1a and SREBP1c after Vemurafenib (5 μM) treatment, data are represented as mean ± s.e.m.

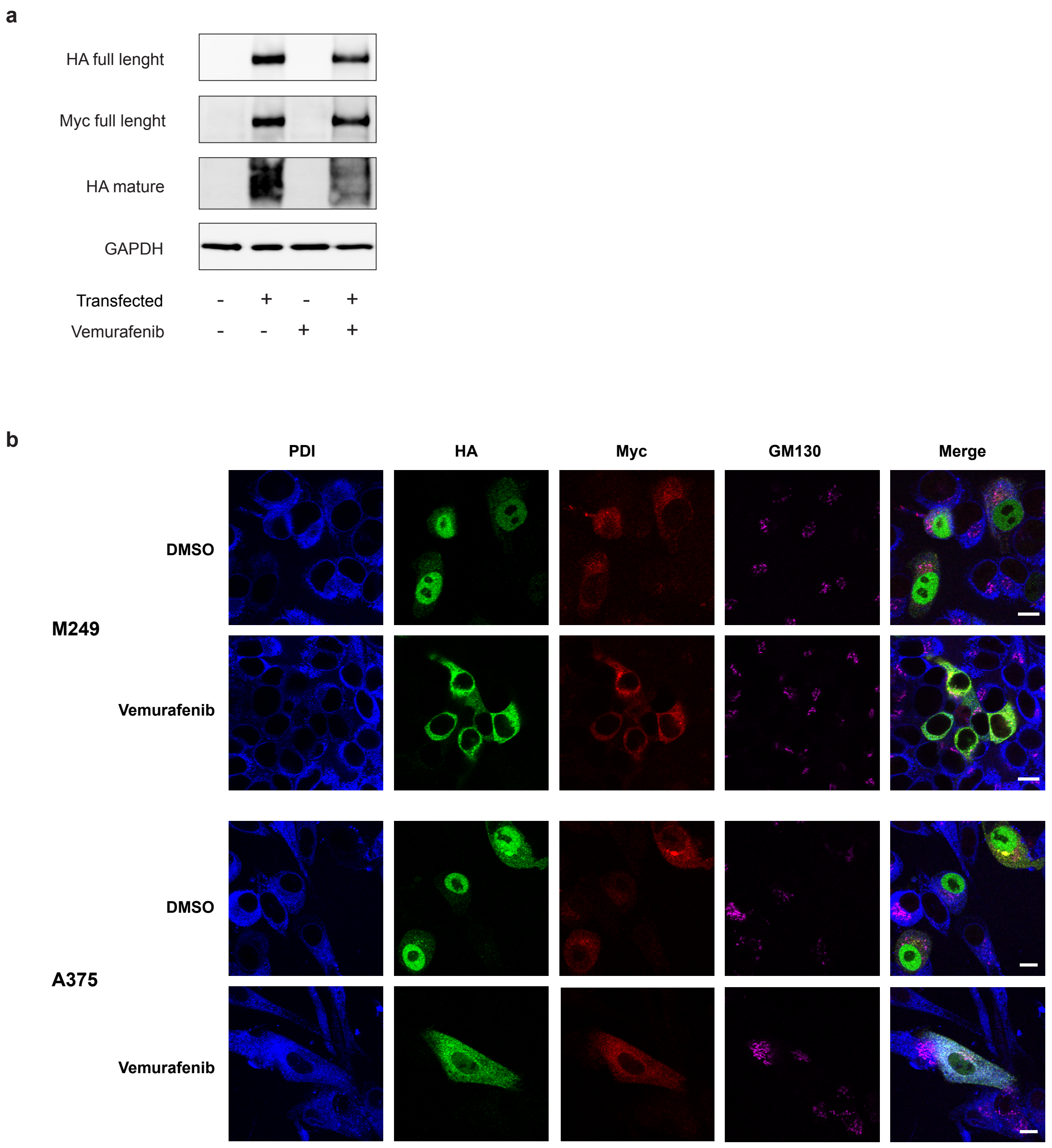

Supplementary Figure 5 (a) Western blot analysis of the HA-SREBP1(Y337R)-MYC construct in response to vemurafenib (5 µM) treatment in 451lu cells (b) Confocal analysis of M249 and A375 cells expressing HA-SREBP1(Y335R)-MYC treated with vemurafenib. A PDI antibody was used to mark the ER and GM130 was used for cis-Golgi. Scale bar indicates 25 µM.

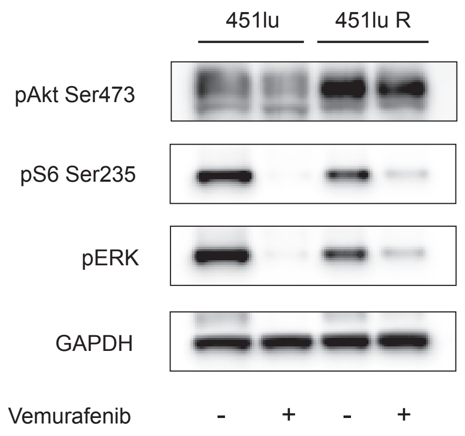

Supplementary Figure 6 Western blot analysis of pAKT, pS6, pERK and GAPDH in response to trametinib (0.5 µM) treatment in 451lu and 451lu R.

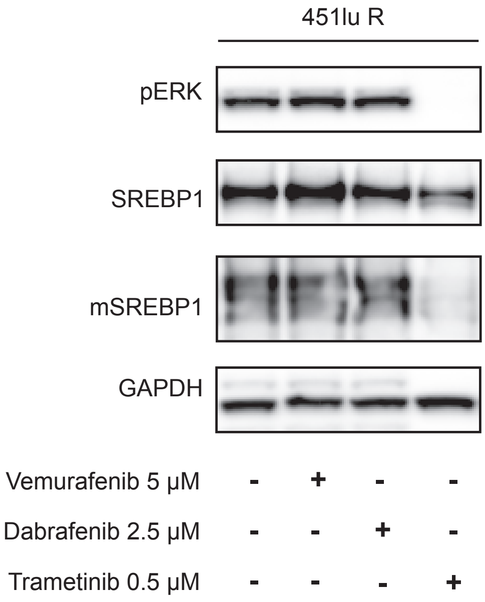

Supplementary Figure 7 Western blot analysis of pERK, GAPDH and SREBP-1 in the cell lines 451lu and 451luR in response to vemurafenib (5 µM), dabrafenib (2.5 µM) and trametinib (0.5 µM).

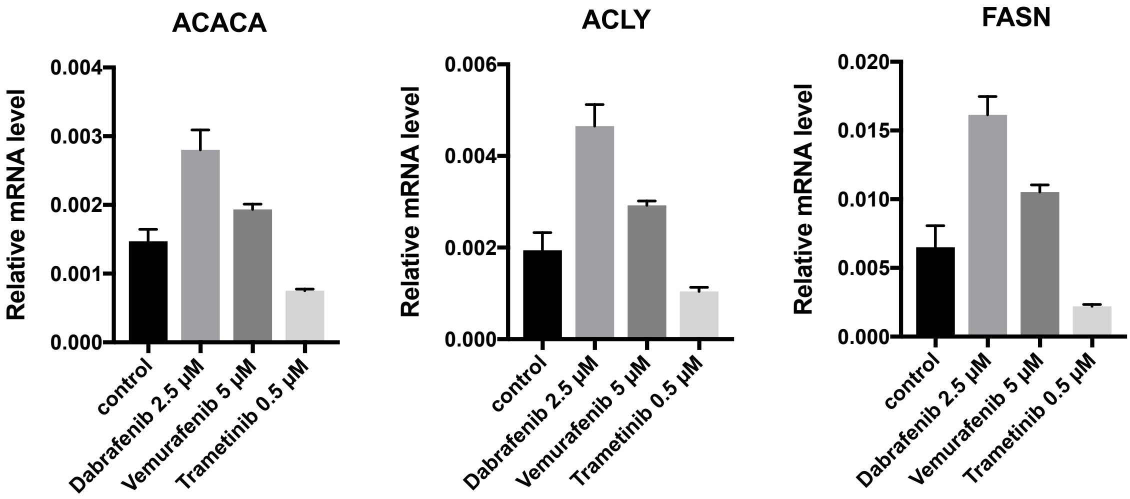

Supplementary Figure 8 RT-qPCR analysis of ACLY, FASN and ACACA normalized to 18S, in 451luR in response to vemurafenib, dabrafenib or trametinib. Data are represented as mean ± s.e.m.

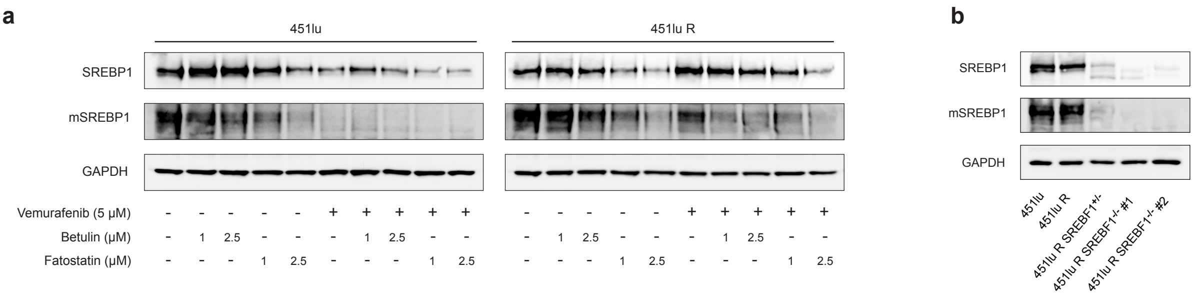

c

451lu R SREBF1$^{+/-}$

```
Ref:      GCGGCCCGGCGGGCCTCCCGGAGGAGGCGGCTGCGCCATGGACGAGCCACCCTTCA-GCGAGGCGGCTTTGGA
Allele 1: GCGGCCCGGCGGGCCTCCCGGAGGAGGCGGCTGCGCCATGGACGAGCCACCCTTCA----------CTTTGGA
Allele 2: GCGGCCCGGCGGGCCTCCCGGAGGAGGCGGCTGCGCCATGGACGAGCCACCCTTCAAGCGAGGCGGCTTTGGA

delete 9
insert 1
```

451lu R SREBF1$^{-/-}$ #1

```
Ref:      GCGGCCCGGCGGGCCTCCCGGAGGAGGCGGCTGCGCCATGGACGAGCCACCCTTCA-GCGAGGCGGCTTTGGA
Allele 1: GCGGCCCGGCGGGCCTCCCGGAGGAGGCGGCTGCGCCATGGACGAGCCACCCT-----CGAGGCGGCTTTGGA
Allele 2: GCGGCCCGGCGGGCCTCCCGGAGGAGGCGGCTGCGCCATGGACGAGCCA-CCTTCA-GCGAGGCGGCTTTGGA
Allele 3: GCGGCCCGGCGGGCCTCCCGGAGGAGGCGGCTGCGCCATGGACGAGCCACCCTTCAAGCGAGGCGGCTTTGGA

delete 4
delete 1
insert 1
```

451lu R SREBF1$^{-/-}$ #2

```
Ref:      GCGGCCCGGCGGGCCTCCCGGAGGAGGCGGCTGCGCCATGGACGAGCCACCCTTCAGCGAGGCGGCTTTGGA
Allele 1: GCGGCCC------------------------------------------------AGCGAGGCGGCTTTGGA
Allele 2: GCGGCCCGGCGGGCCTCCCGGAGGAGGCGGCTGCGCCATGGACGAGCCACCCT-CAGCGAGGCGGCTTTGGA

delete 48 (loss of start codon)
delete 1
```

Legend

SREBF1 Exon 1

Start codon

Supplementary Figure 9 (a) Western blot analysis for SREBP-1 levels in response to betulin or fatostatin treatment combined with vemurafenib (5 µM) in responsive and resistant cells. (b) Western blot analysis for SREBP-1 levels in CRISPR-Cas9 knockouts of SREBF1. (c) Genetic profiling of CRISPR-Cas9 knockouts of SREBF1.

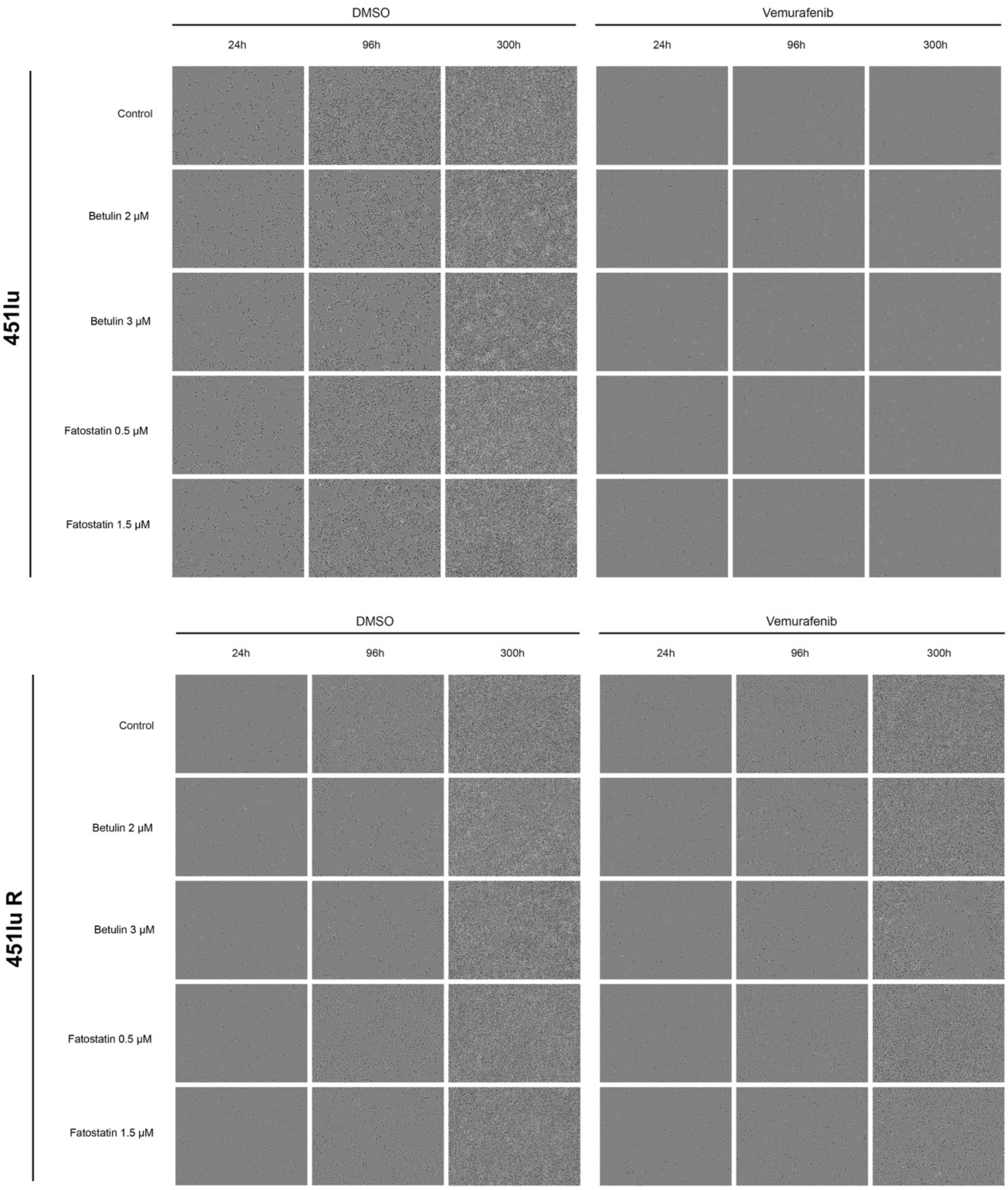

Supplementary Figure 10 Proliferation of 451lu and 451lu R cells treated with vemurafenib (5 µM) and fatostatin or betulin.

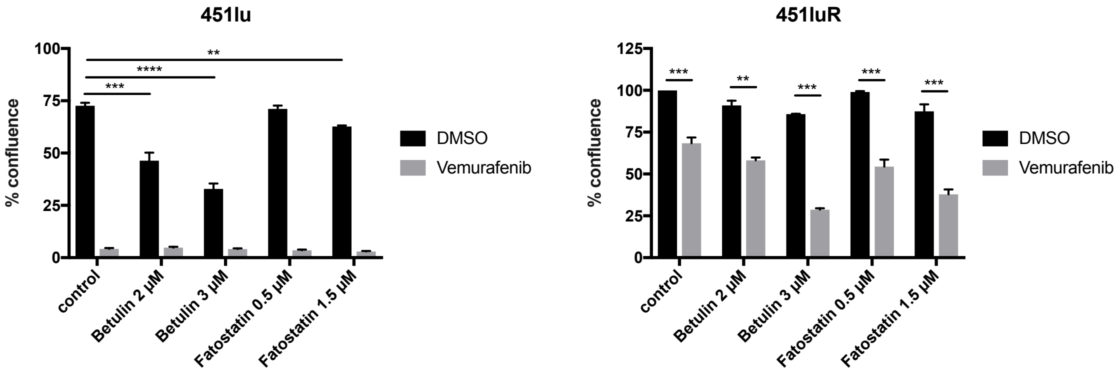

Supplementary Figure 11 Proliferation of 451lu (time point 96h) and 451lu R (time point 168h) cells treated with vemurafenib (5 µM) and fatostatin or betulin. Data was taken from the experiment shown in main figure 3C. Time points chosen represent those where the fastest growing conditions started approaching confluence. Data are represented as mean ± s.e.m.

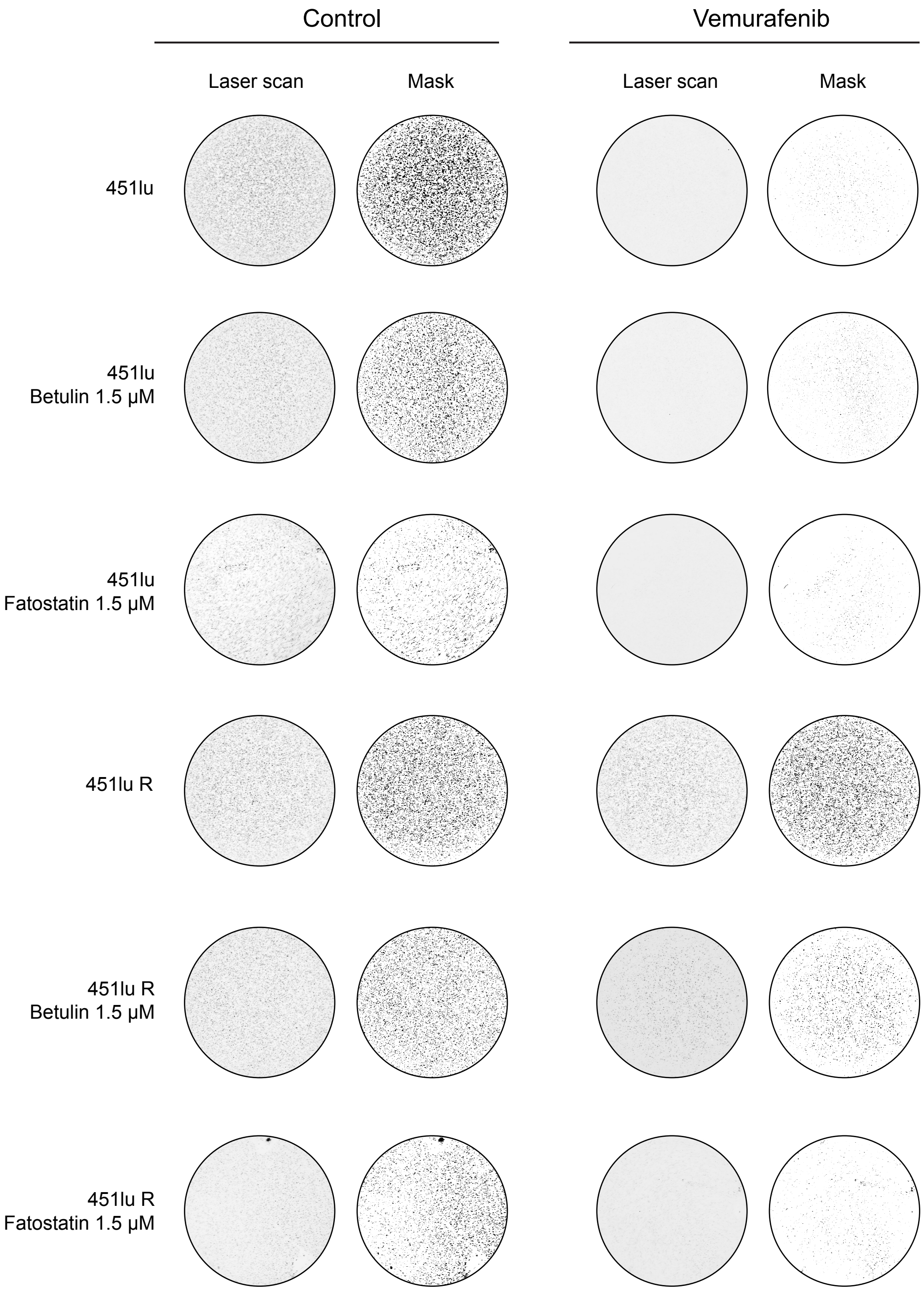

Supplementary Figure 12 451lu and 451lu R cells were treated with SREBP inhibitors and vemurafenib (5 μM) and were assayed for their ability to form colonies in soft agar.

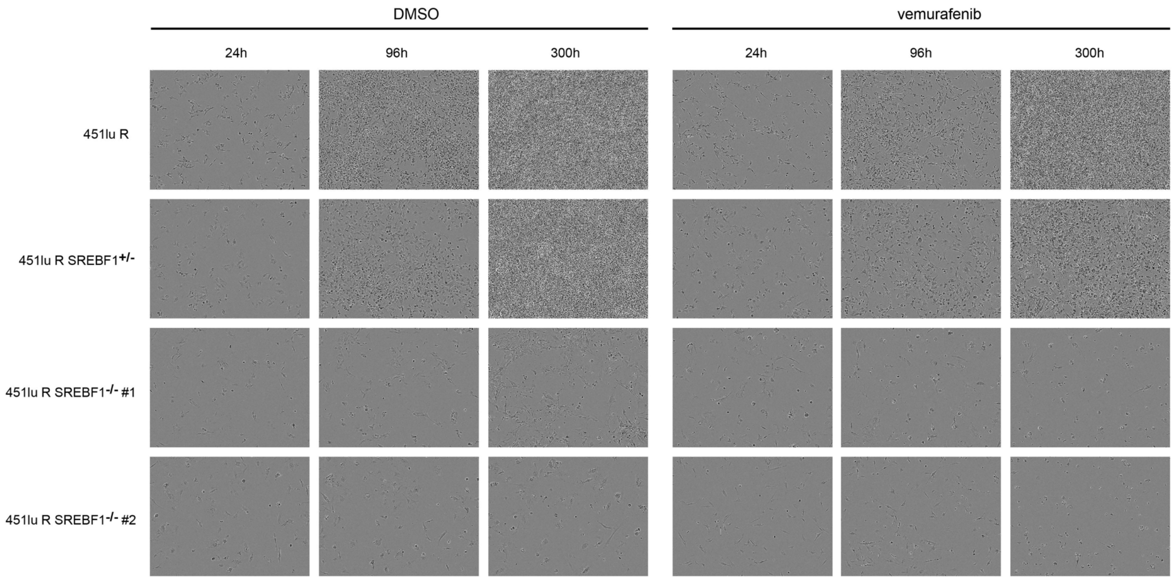

Supplementary Figure 13 Proliferation of CRISPR-Cas9 knockouts of SREBF1 in combination with vemurafenib (5 µM) treatment.

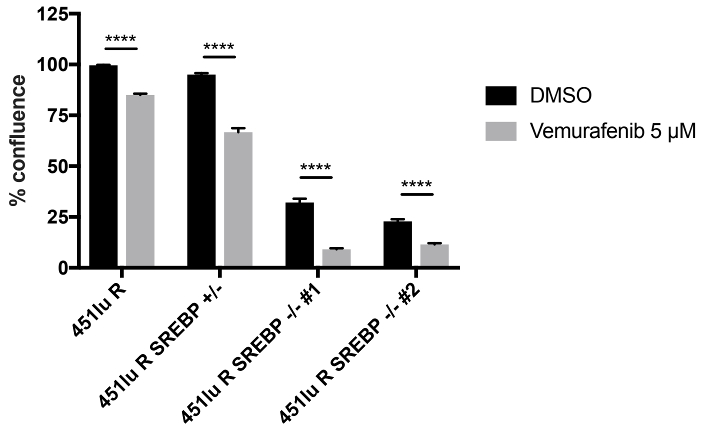

Supplementary Figure 14 Proliferation of CRISPR-Cas9 knockouts of SREBF1 in combination with vemurafenib (5 µM) treatment. Data was taken from the experiment shown in main figure 3E (time point 144h). Data are represented as mean ± s.e.m.

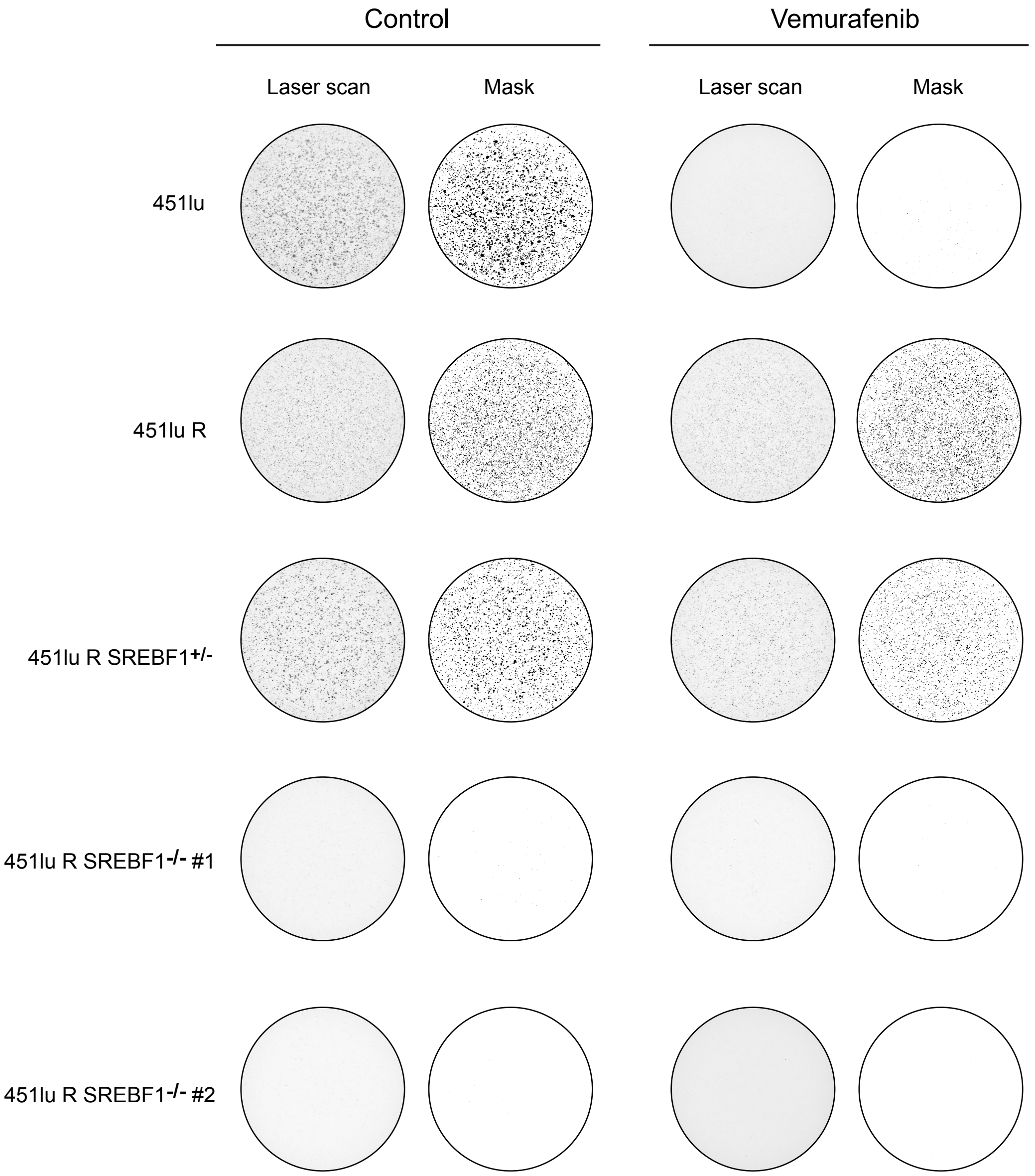

Supplementary Figure 15
SREBP KO 451lu R cells were assayed for their ability to form colonies in soft agar in presence or absence of vemurafenib (5 µM).

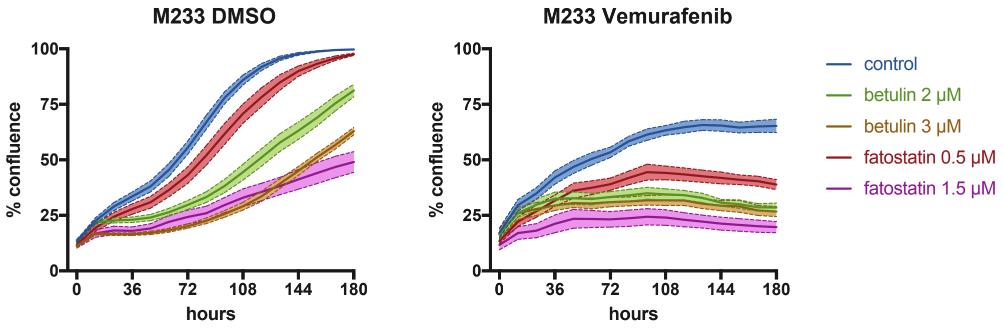

Supplementary Figure 16

Proliferation curves of M233 cells treated with vemurafenib (5 µM) and fatostatin or betulin (phase contrast density measured by the Incucyte system). Data are represented as mean ± s.e.m.

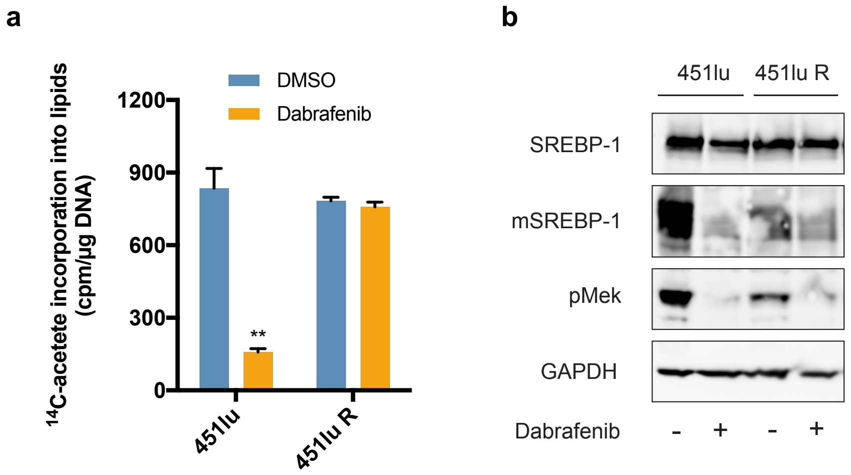

Supplementary Figure 17

(a) Dabrafenib (2.5 µM) or vehicle treated cells were assayed for their ability to incorporate ^{14}C acetate into lipids. Data are represented as mean ± s.e.m. The significance was determined with an unpaired t test and compares dabrafenib treated cells to their matching controls. (**p < 0.01). (b) Western blot analysis for mSREBP levels in response to dabrafenib treatment.

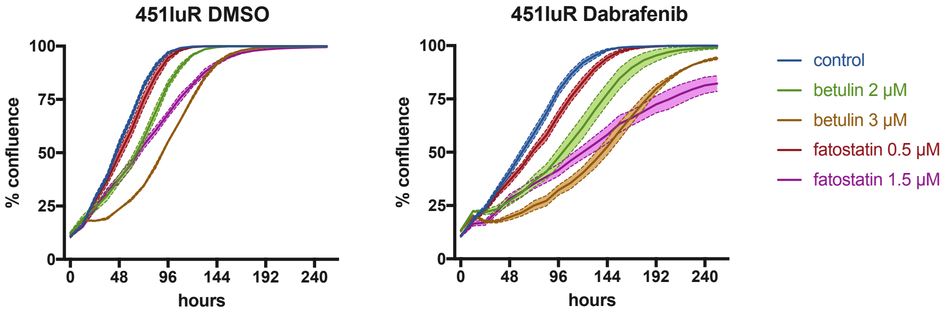

Supplementary Figure 18

Proliferation curves of 415luR cells treated with dabrafenib (2.5 μM) and fatostatin or betulin (phase contrast density measured by the Incucyte system). Data are represented as mean ± s.e.m.

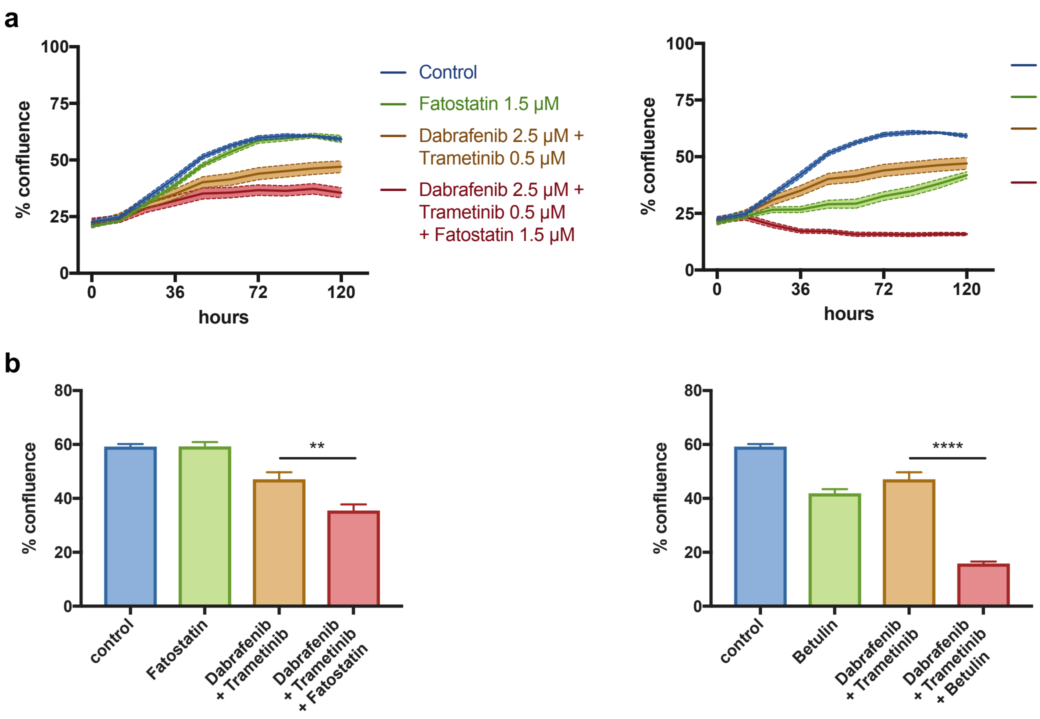

Supplementary Figure 19

(a) Proliferation curves of the dabrafenib + trametinib double resistant cell line A101D BMR treated with dabrafenib, trametinib, betulin and fatostatin (phase contrast density measured by the Incucyte system). (b) Bar graph of time point 120h. Data are represented as mean ± s.e.m.

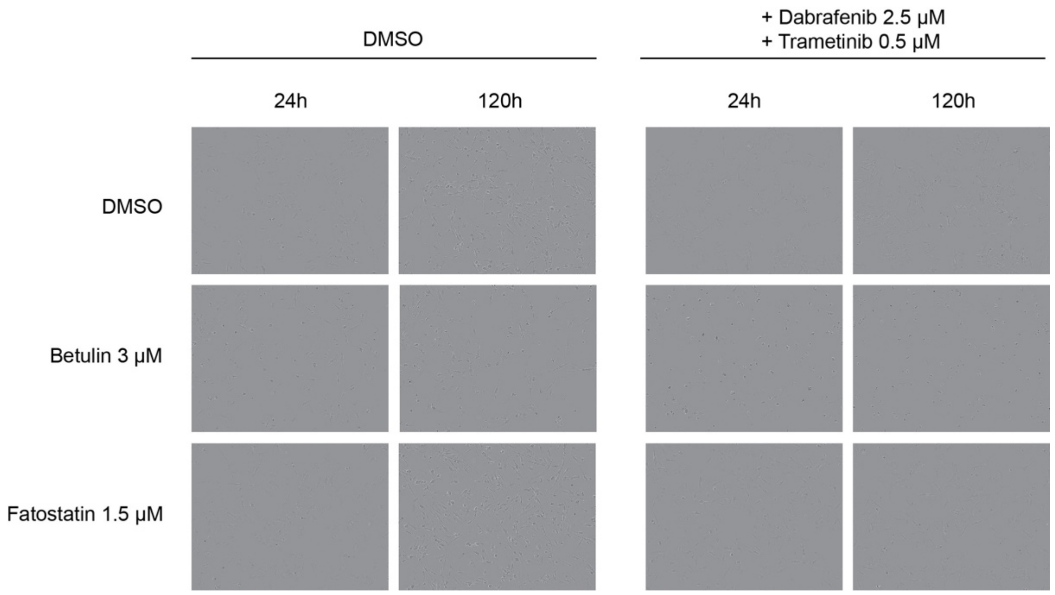

Supplementary Figure 20
Photomicrographs of the dabrafenib + trametinib double resistant cell line A101D BMR treated with dabrafenib, trametinib, betulin and fatostatin (phase contrast density measured by the Incucyte system).

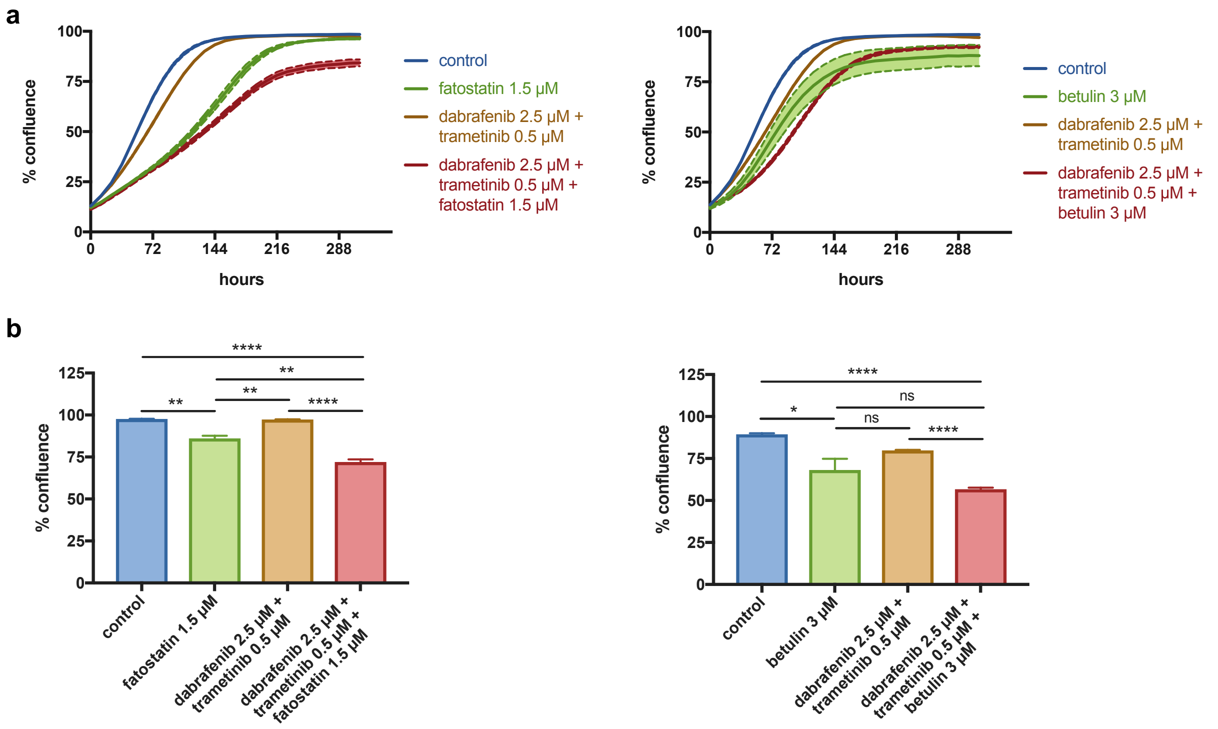

Supplementary Figure 21

(a) Proliferation curves of the dabrafenib + trametinib double resistant cell line D10 BMR treated with dabrafenib, trametinib, betulin and fatostatin (phase contrast density measured by the Incucyte system). (b) Bar graph of time point 192h for the fatostatin experiment, 108h for the betulin experiment. Data are represented as mean ± s.e.m.

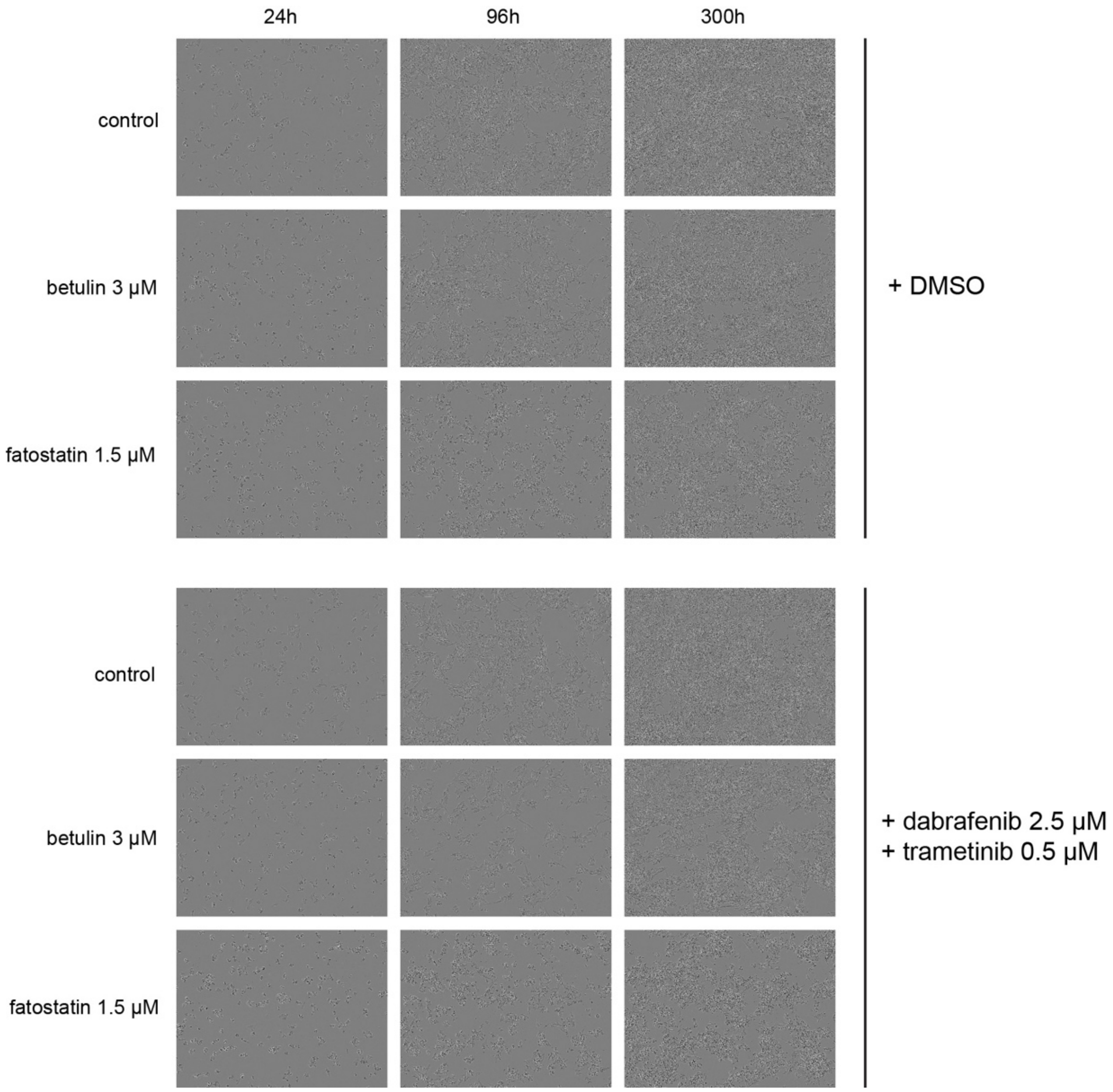

Supplementary Figure 22
Photomicrographs of the dabrafenib + trametinib double resistant cell line D10 BMR treated with dabrafenib, trametinib, betulin and fatostatin (phase contrast density measured by the Incucyte system).

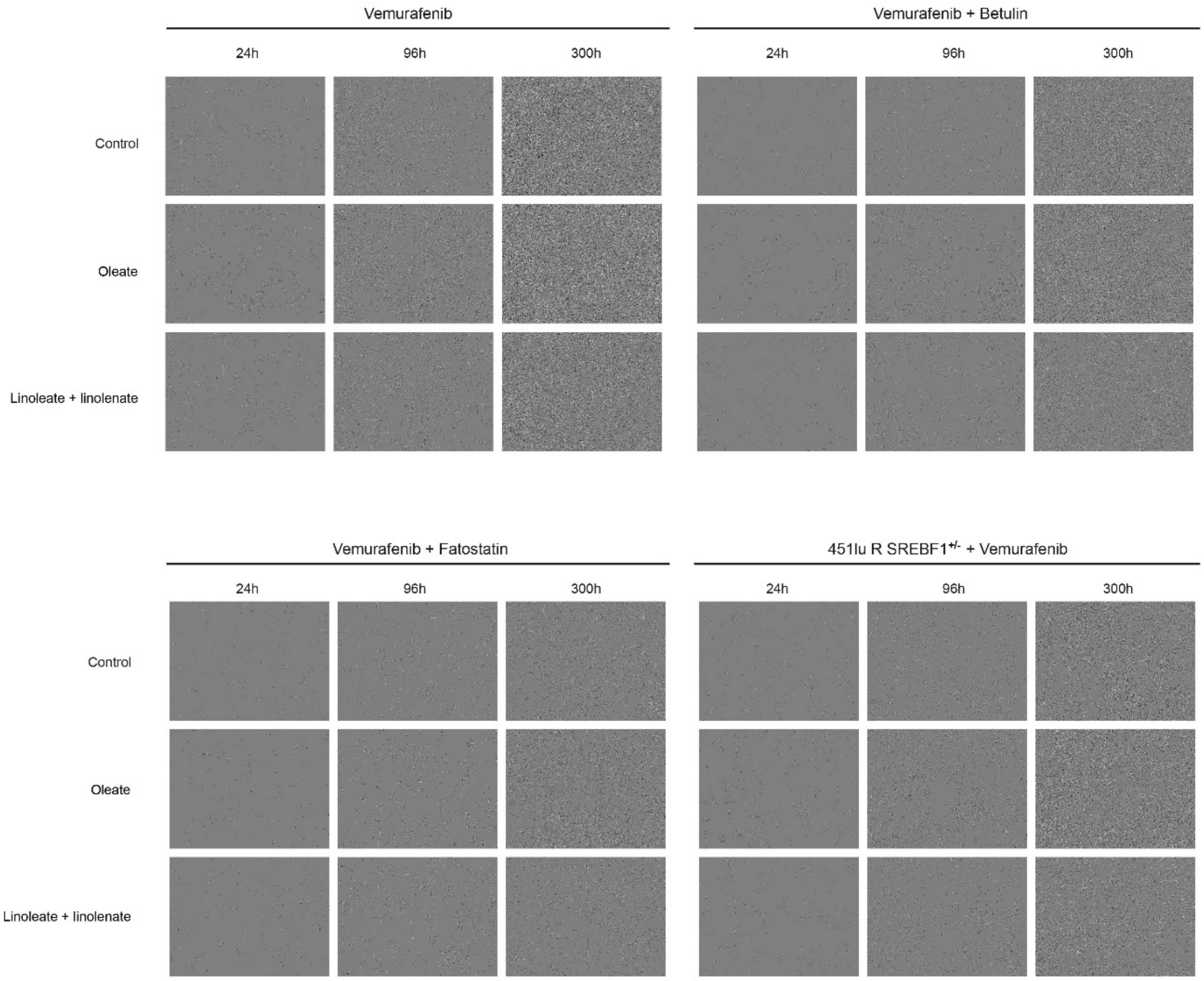

Supplementary Figure 23
Oleate (5 µM) or a mixture of linoleate (10 µM) + linolenate (10 µM) was added to the culture medium of 451lu R SREBF1$^{+/-}$ cells and 451lu R cells treated with either vemurafenib alone or a combination with fatostatin or betulin.

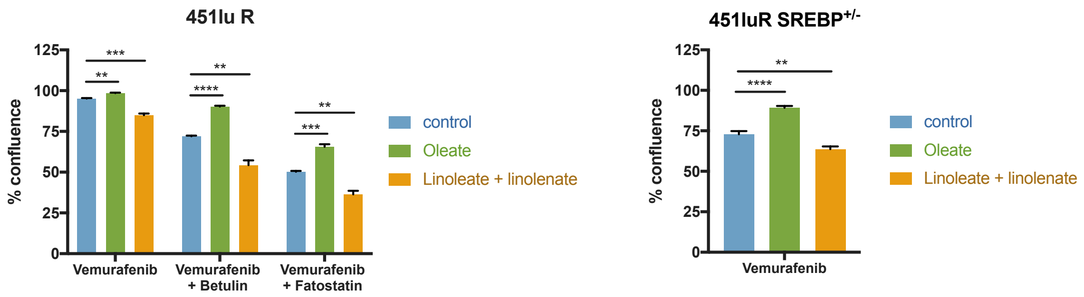

Supplementary Figure 24

Oleate (5 µM) or a mixture of linoleate (10 µM) + linolenate (10 µM) was added to the culture medium of 451lu R SREBF1$^{+/-}$ cells and 451lu R cells treated with either vemurafenib alone or a combination with fatostatin or betulin**.** Data was taken from the experiment shown in main figure 4A (time point 216h for 451luR and 168h for 451luR SREBP$^{+/-}$). Data are represented as mean ± s.e.m.

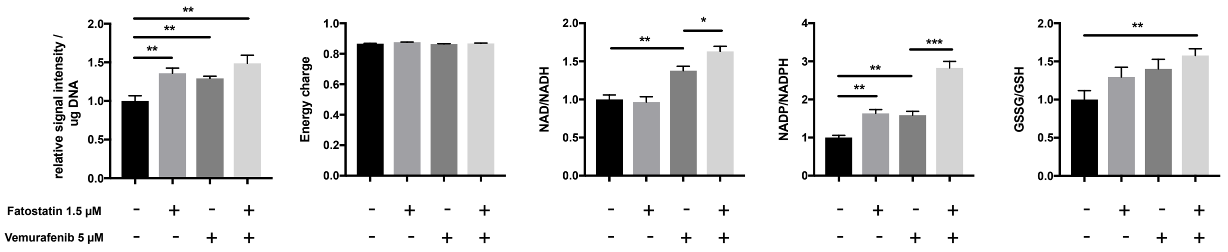

Supplementary Figure 25

Relative ATP concentration, energy charge, NAD/NADH, NADP/NADPH and GSSG/GSH ratios in 451luR were obtained by untargeted metabolomics following vemurafenib and fatostatin treatment. Data are represented as mean ± s.e.m.

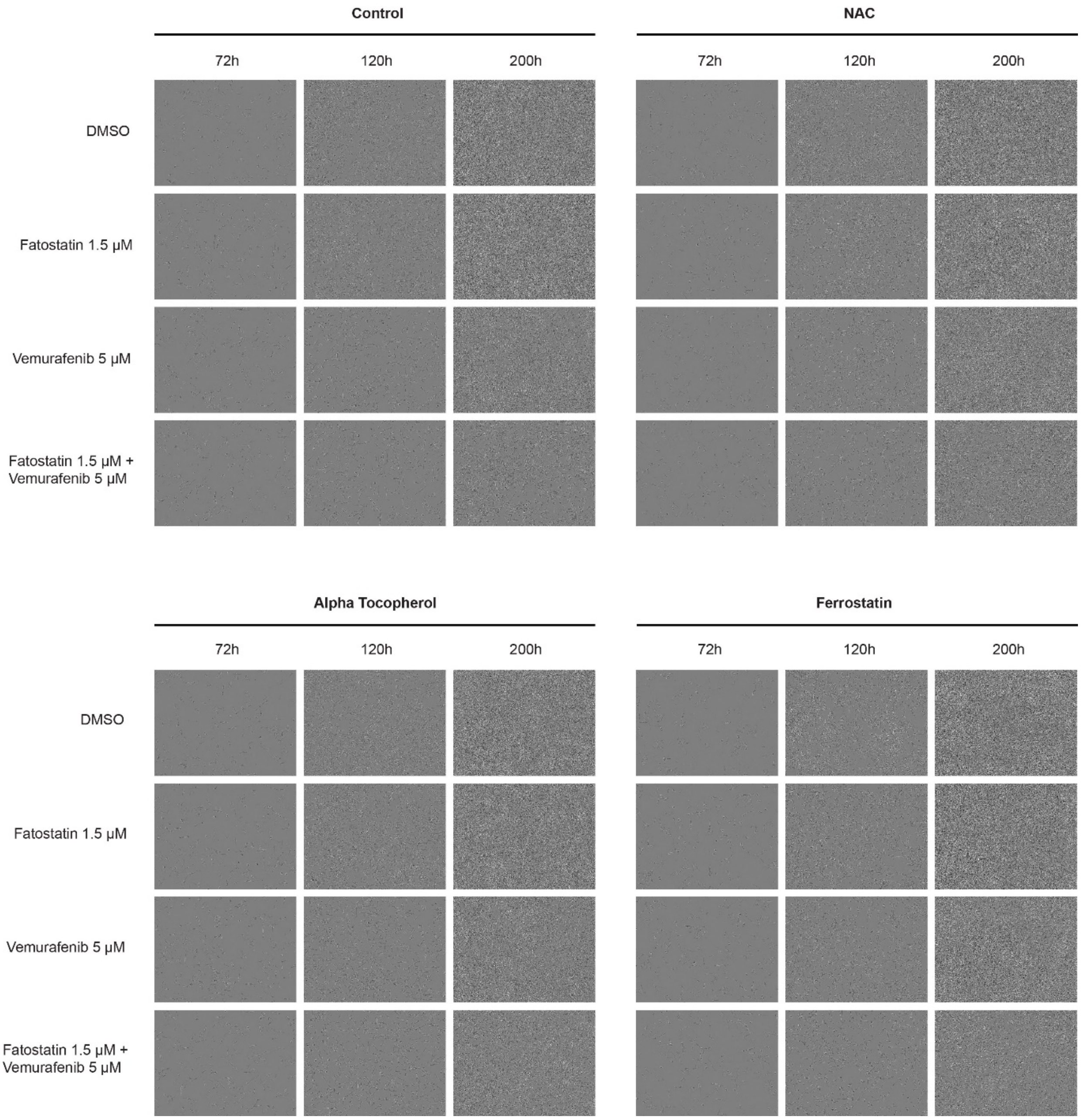

Supplementary Figure 26

Alpha-tocopherol (100 µM), ferrostatin (1.25 µM) or NAC (120 µM) were added to the culture medium of 451lu R cells and were treated with either vemurafenib (5 µM) alone or a combination with fatostatin or betulin.

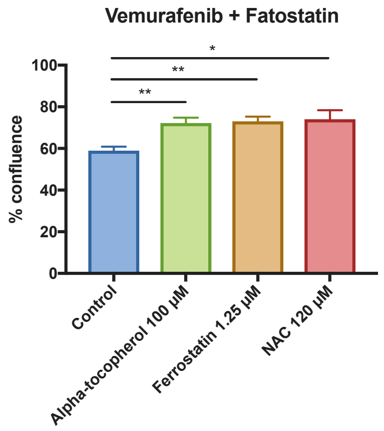

Supplementary Figure 27

Alpha-tocopherol (100 µM), ferrostatin (1.25 µM) or NAC (120 µM) were added to the culture medium of 451lu R cells and were treated with either vemurafenib (5 µM) alone or a combination with fatostatin or betulin**.** Data was taken from the experiment shown in main figure 4D (time point 192h). Data are represented as mean ± s.e.m.

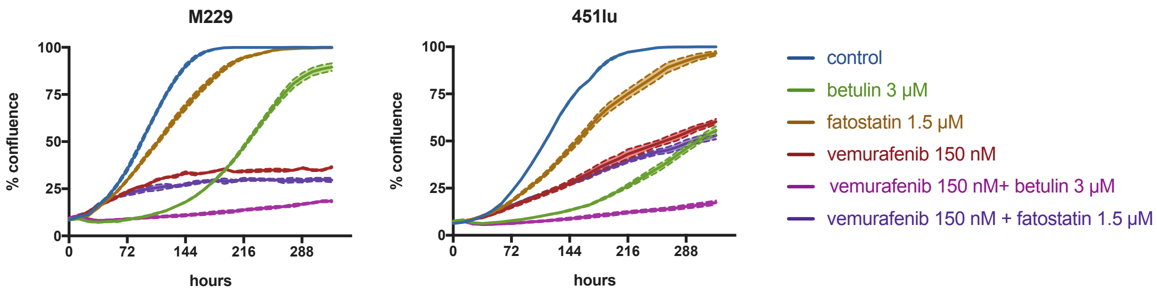

Supplementary Figure 28

Proliferation curves of the therapy-sensitive cell lines M229 and 451lu treated with vemurafenib, betulin and fatostatin (phase contrast density measured by the Incucyte system). Data are represented as mean ± s.e.m.

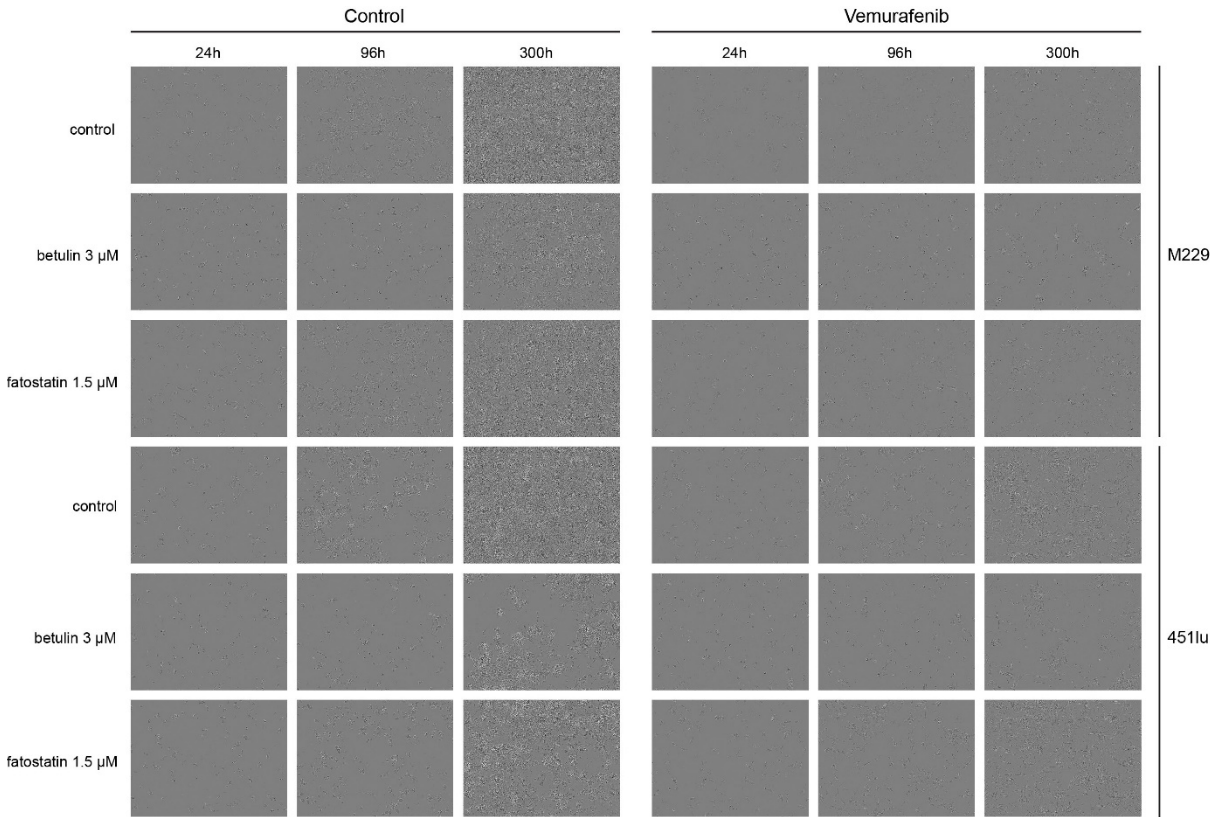

Supplementary Figure 29
Representative photomicrographs of the therapy-sensitive cell lines M229 and 451lu treated with vemurafenib, betulin and fatostatin (phase contrast density measured by the Incucyte system).

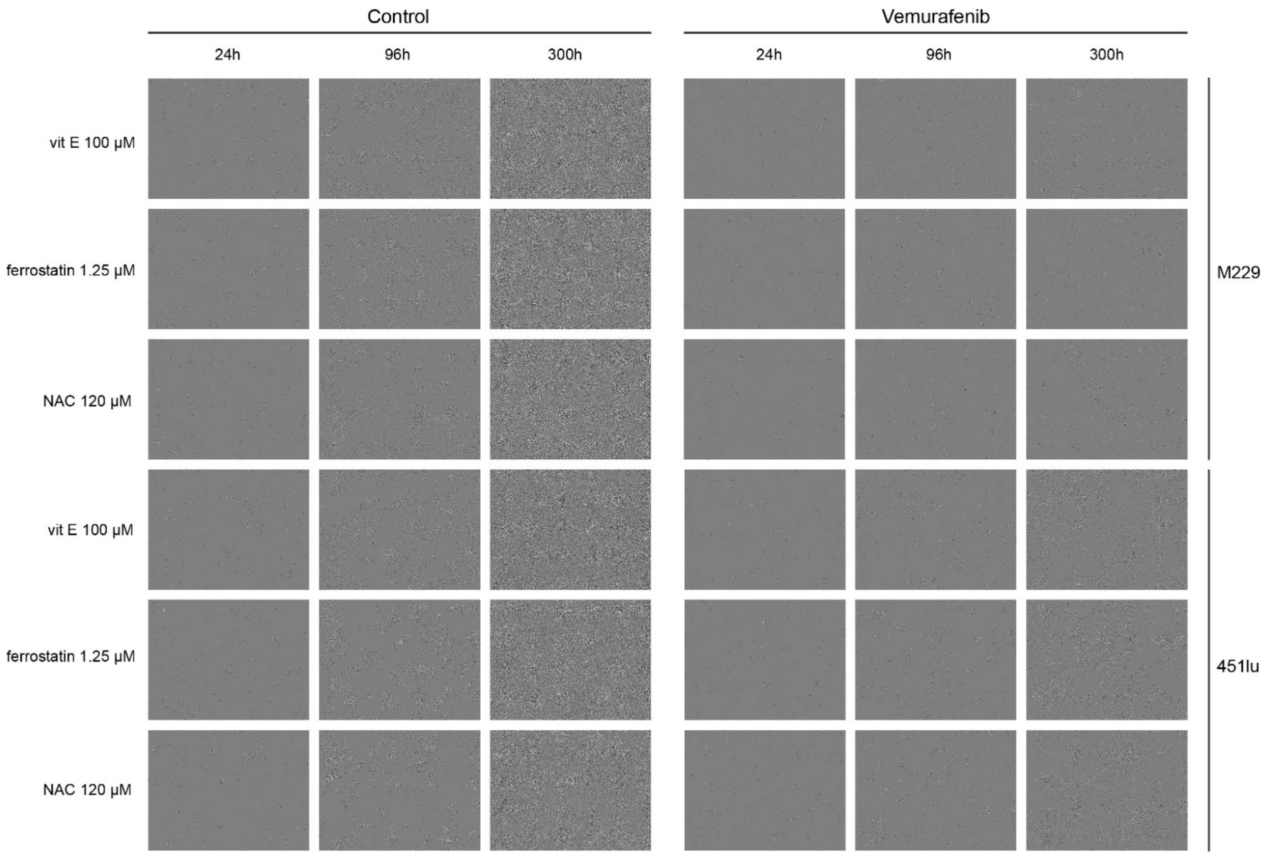

Supplementary Figure 30
Representative photomicrographs of the therapy-sensitive cell lines M229 and 451lu treated with vemurafenib, alpha-tocopherol, ferrostatin and NAC (phase contrast density measured by the Incucyte system).

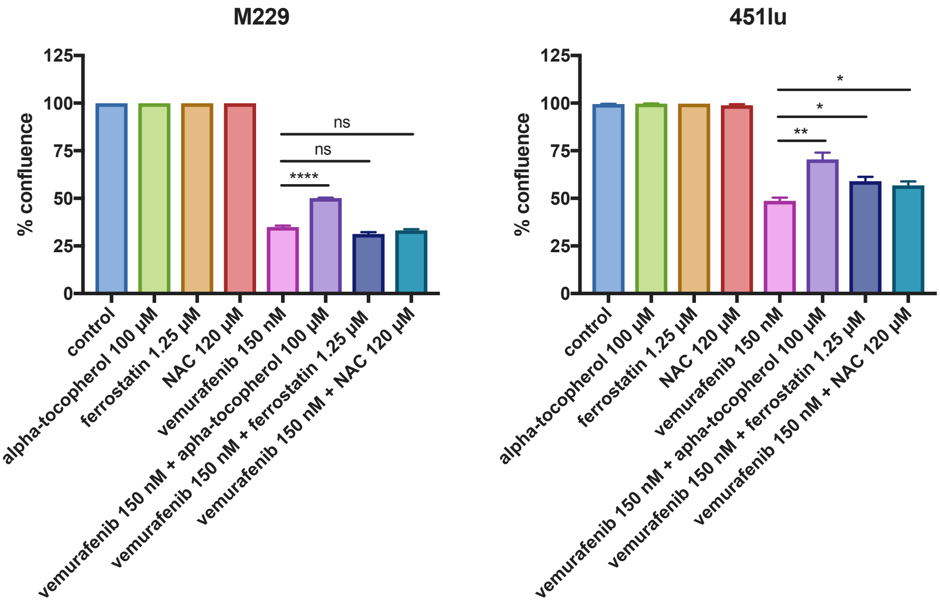

Supplementary Figure 31

Representative photomicrographs of the therapy-sensitive cell lines M229 and 451lu treated with vemurafenib, alpha-tocopherol, ferrostatin and NAC (phase contrast density measured by the Incucyte system). Data was taken from the experiment shown in main figure 4E (time point 253h). Data are represented as mean ± s.e.m.

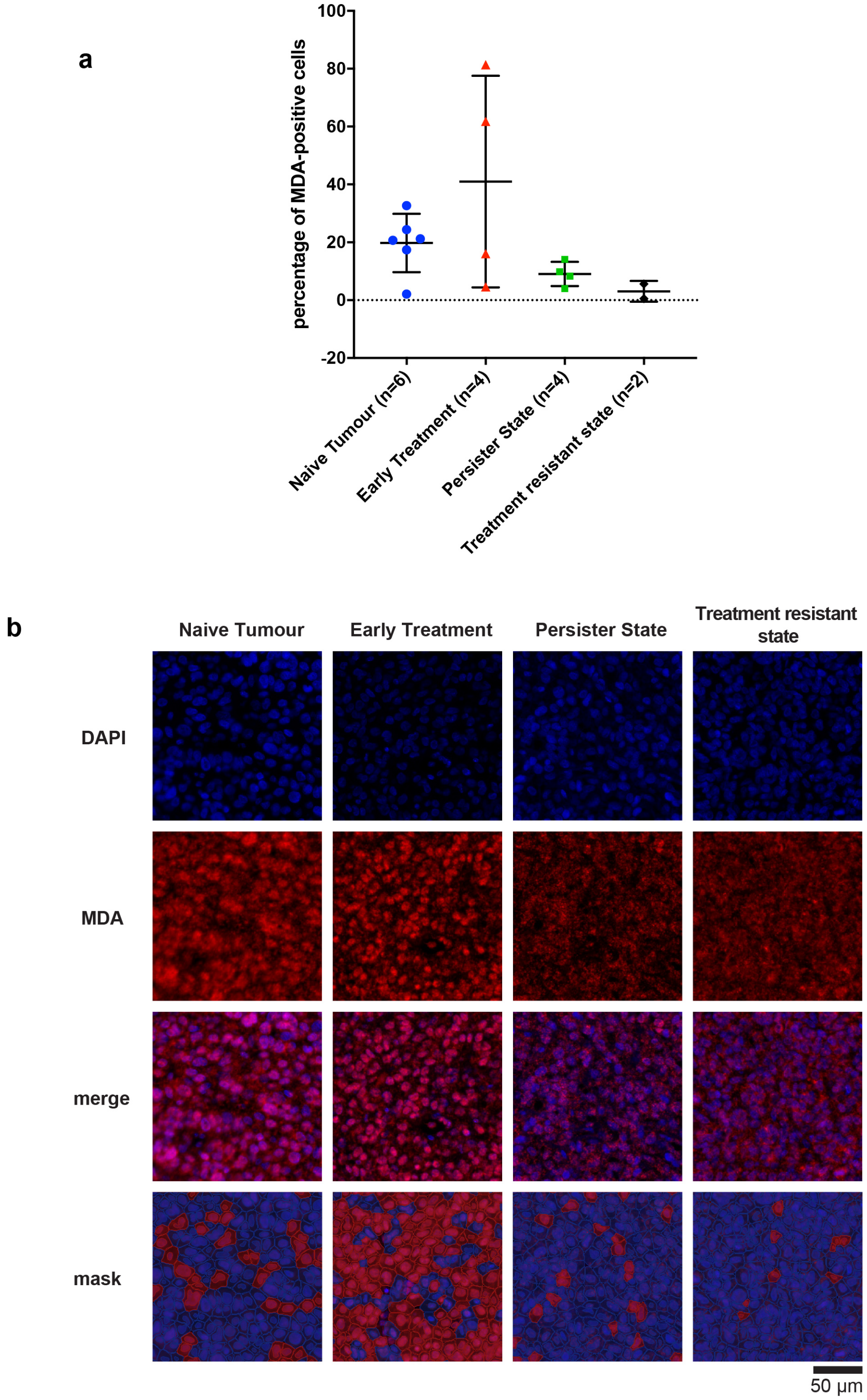

Supplementary Figure 32

(a). Anti MDA quantified histological staining in mel06 tumors. Tumors were stratified into four groups, either the naïve tumors, early treatment following 2 – 4 days of mouse treatment, persister state following two to three weeks of treatment and the tumor resistant state where the tumor develops therapy resistance. Histological staining reveals a non-significant increase in tumor MDA staining shortly after treatment, which is reduced in the persister state and in the resistant tumor. (b) Representative images of anti-MDA (red) staining, nuclei were counterstained with DAPI. Trend is not significant.
