# Supplementary material for: Sustained SREBP-1-dependent lipogenesis as a key mediator of resistance to BRAF-targeted therapy
Source: Nat Commun. 2018 Jun 27;9:2500. doi: 10.1038/s41467-018-04664-0 (PMC6021375; doi:10.1038/s41467-018-04664-0)
Supplement: Supplementary file 3 — Description of Additional Supplementary Files [file 41467_2018_4664_MOESM3_ESM.pdf]

Description of Additional Supplementary Files

File Name: Supplementary Data 1
Description: a collection of the full uncropped western blots of all the blots shown in the main figures.
