# Supplementary material for: Sustained SREBP-1-dependent lipogenesis as a key mediator of resistance to BRAF-targeted therapy
Source: Nat Commun. 2018 Jun 27;9:2500. doi: 10.1038/s41467-018-04664-0 (PMC6021375; doi:10.1038/s41467-018-04664-0)

Full unedited gel for Figure 2B

Full unedited gel for Figure 2B – 451lu

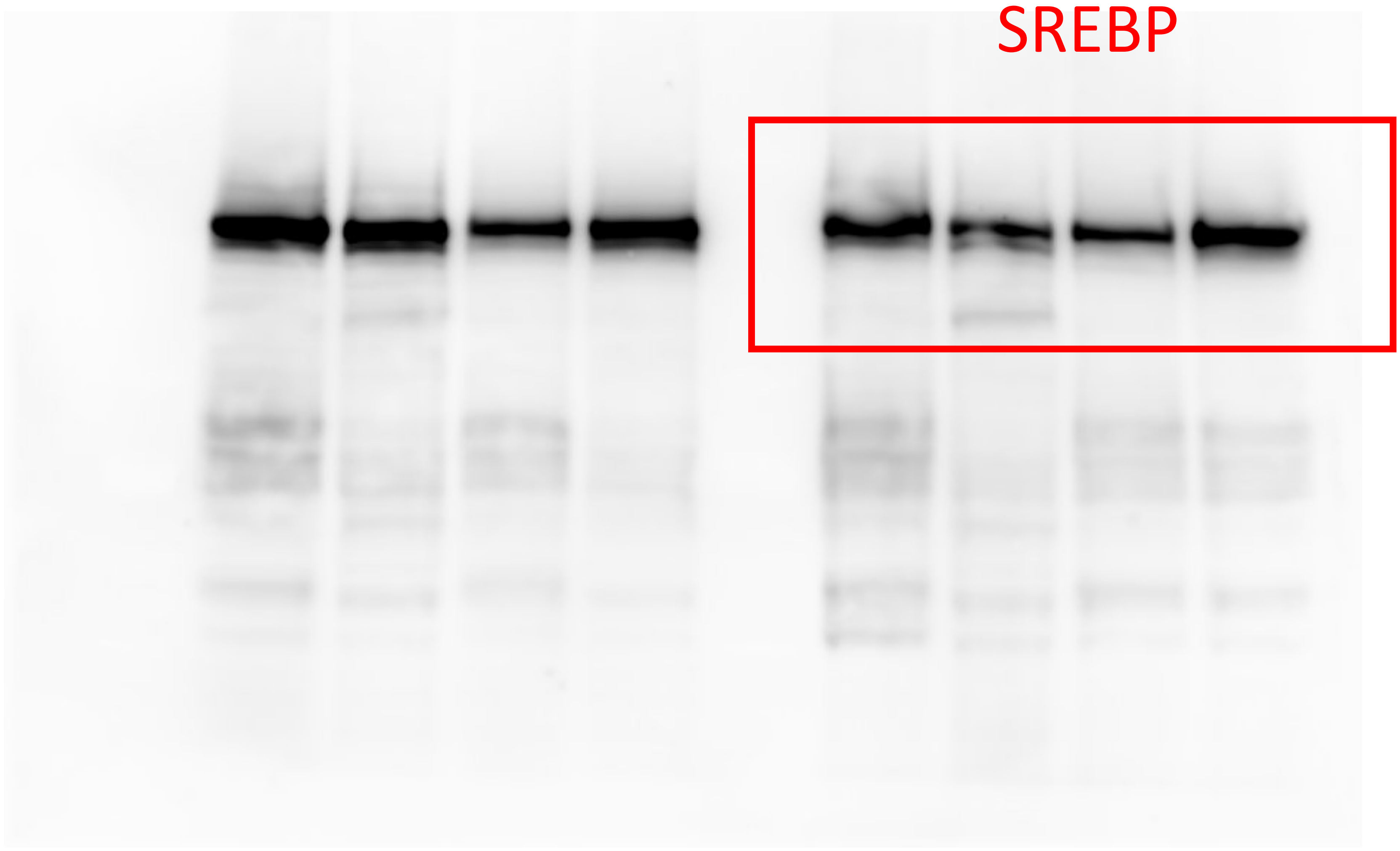

Full unedited gel for Figure 2B – 451lu

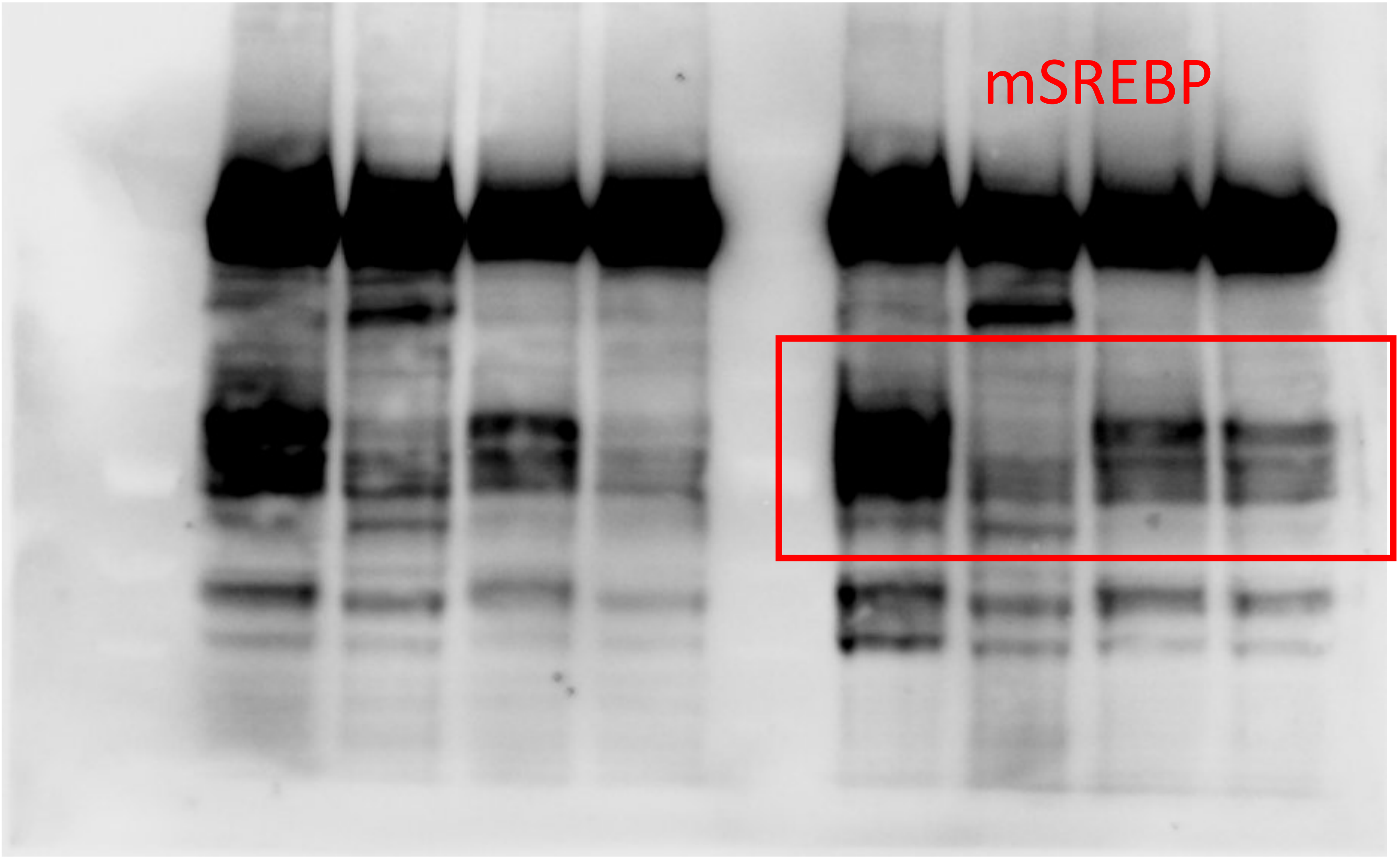

Full unedited gel for Figure 2B – 451lu

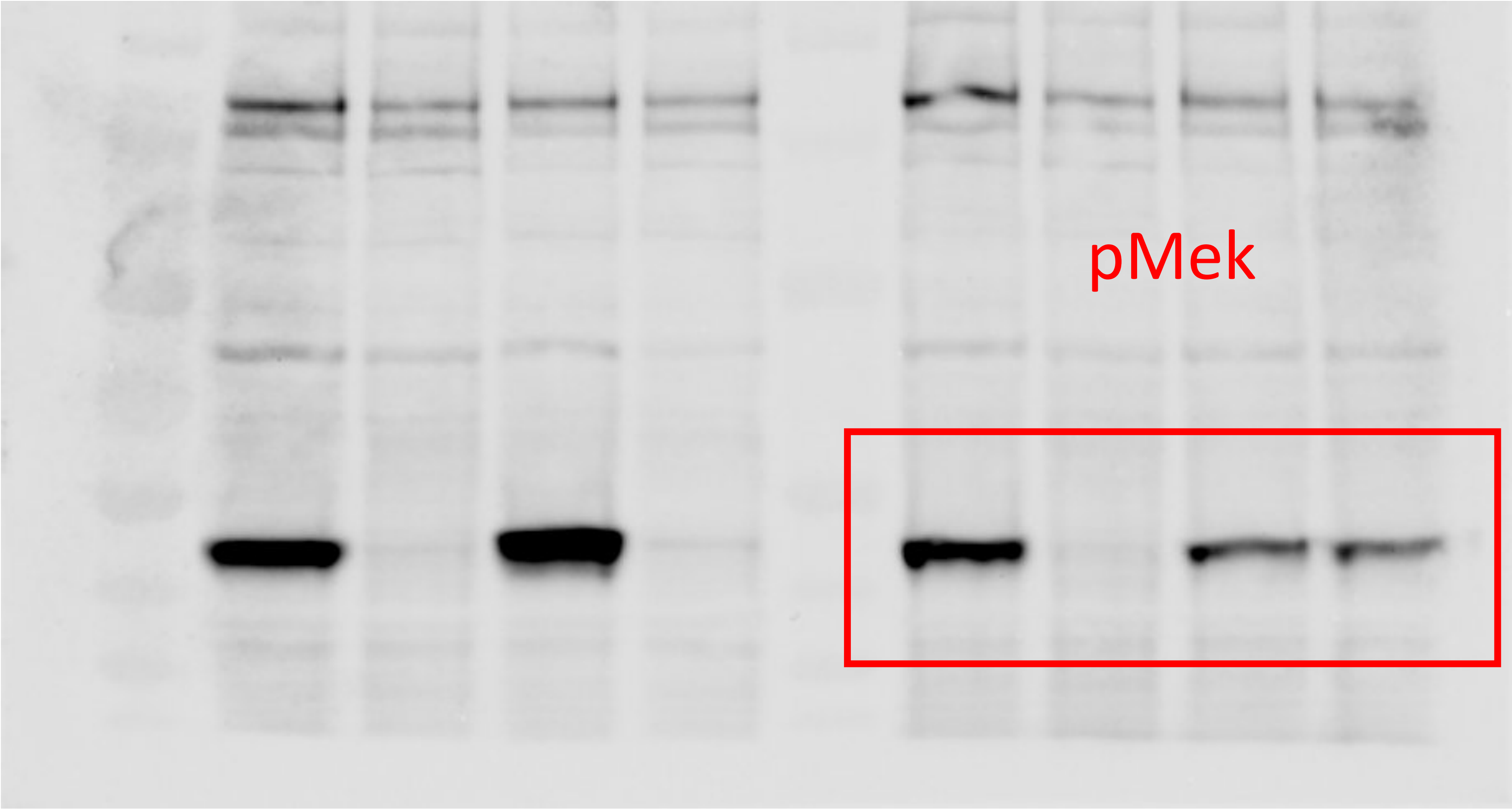

Full unedited gel for Figure 2B – 451lu

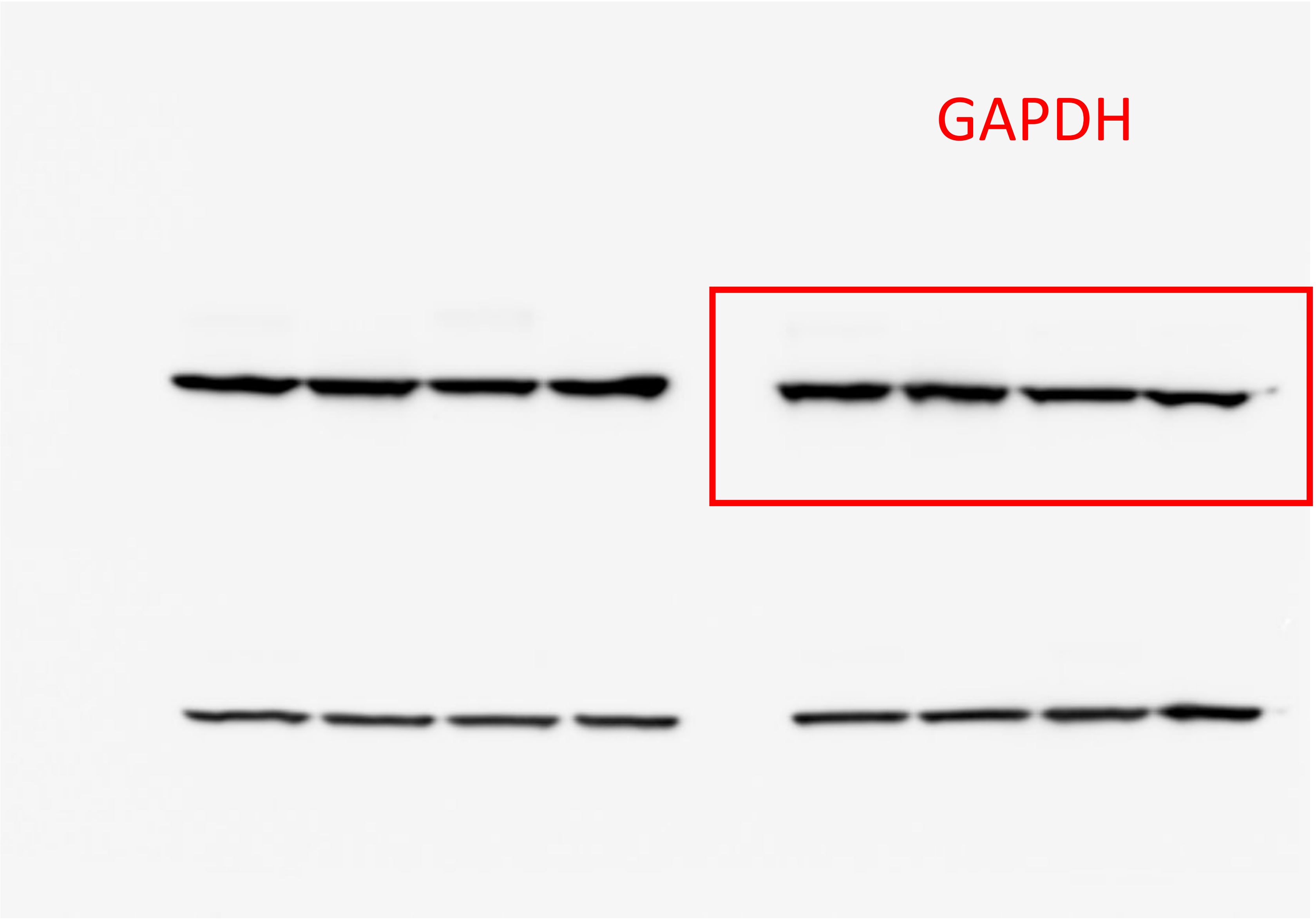

Full unedited gel for Figure 2B – M229

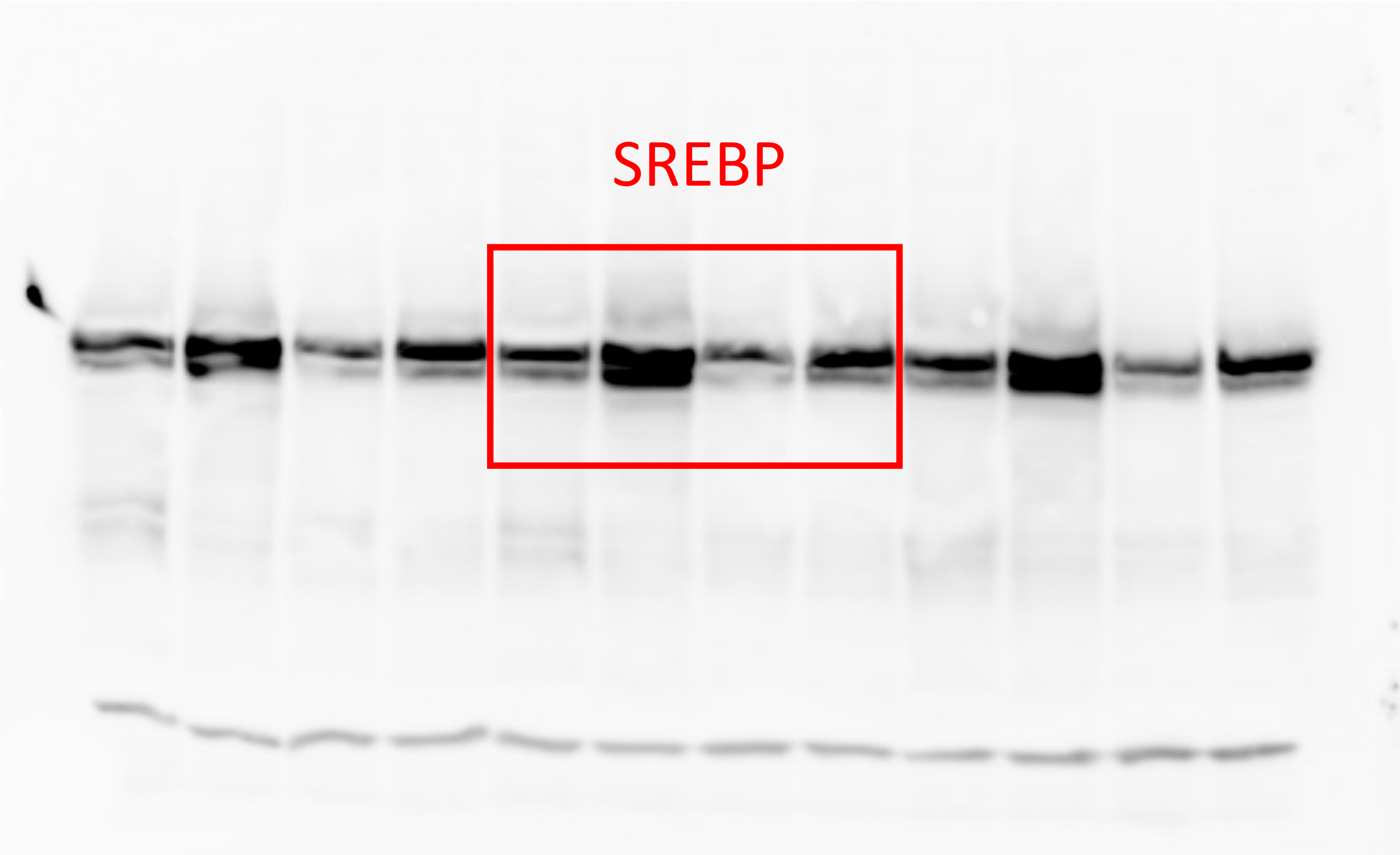

Full unedited gel for Figure 2B – M229

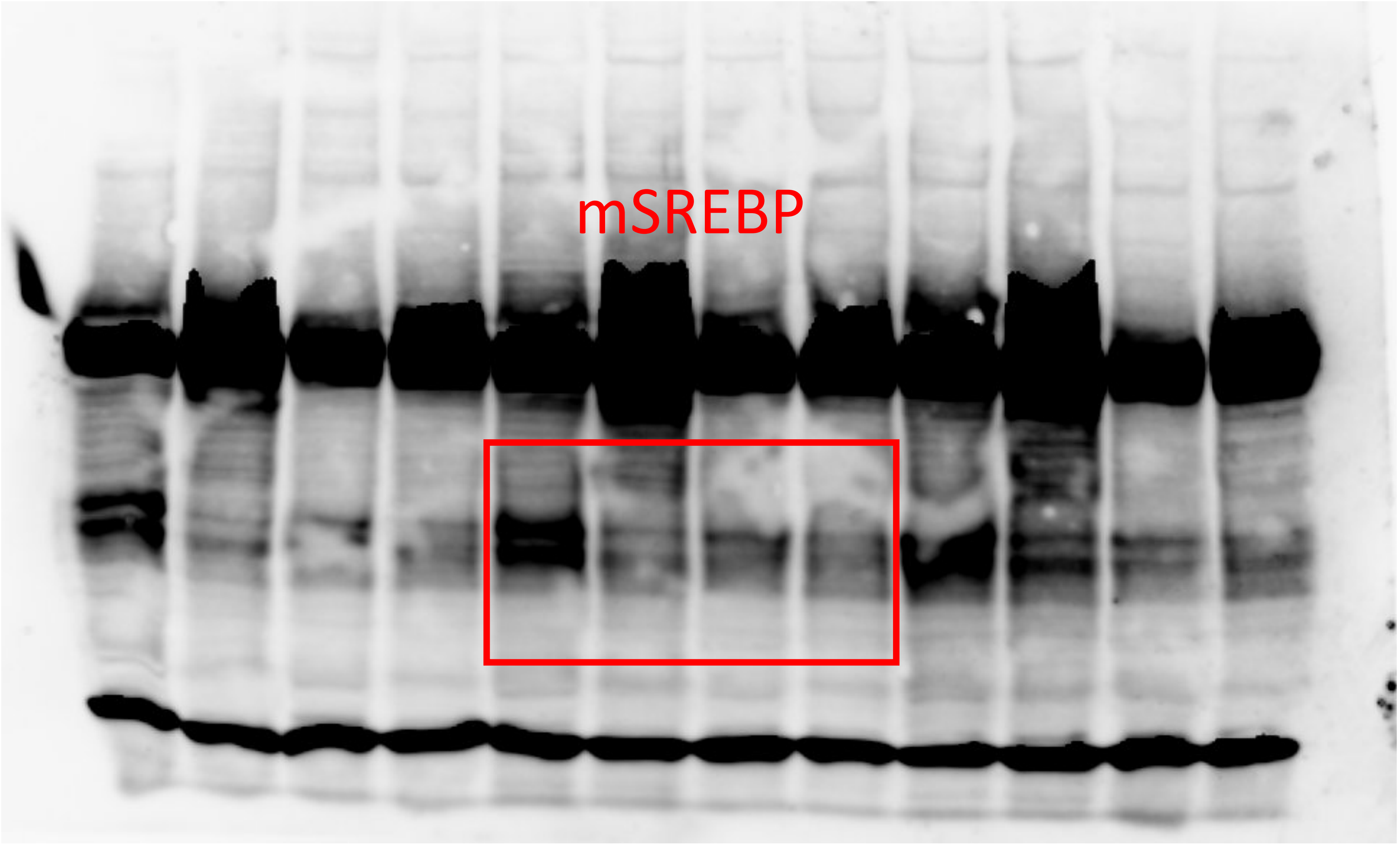

Full unedited gel for Figure 2B – M229

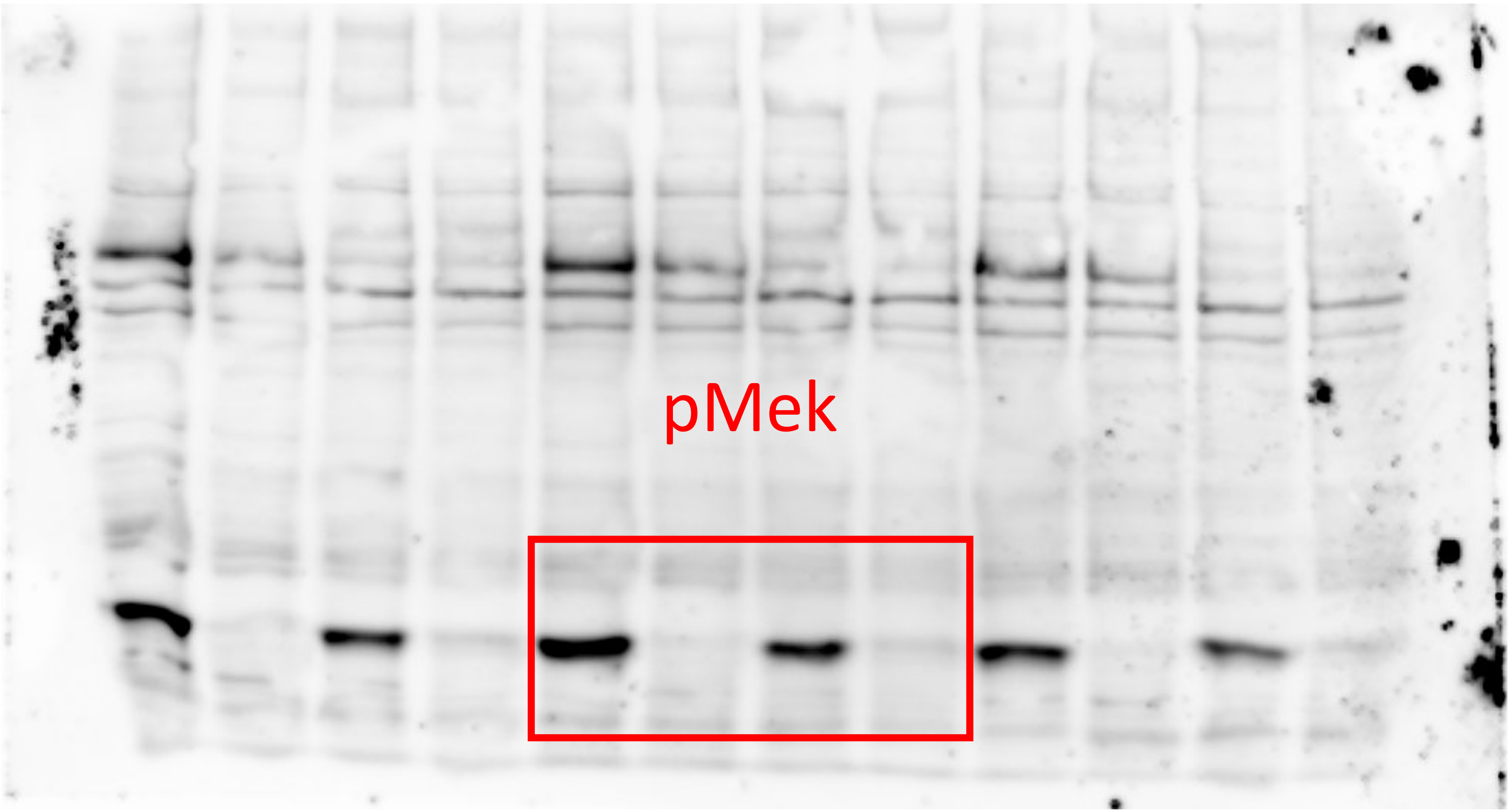

Full unedited gel for Figure 2B – M229

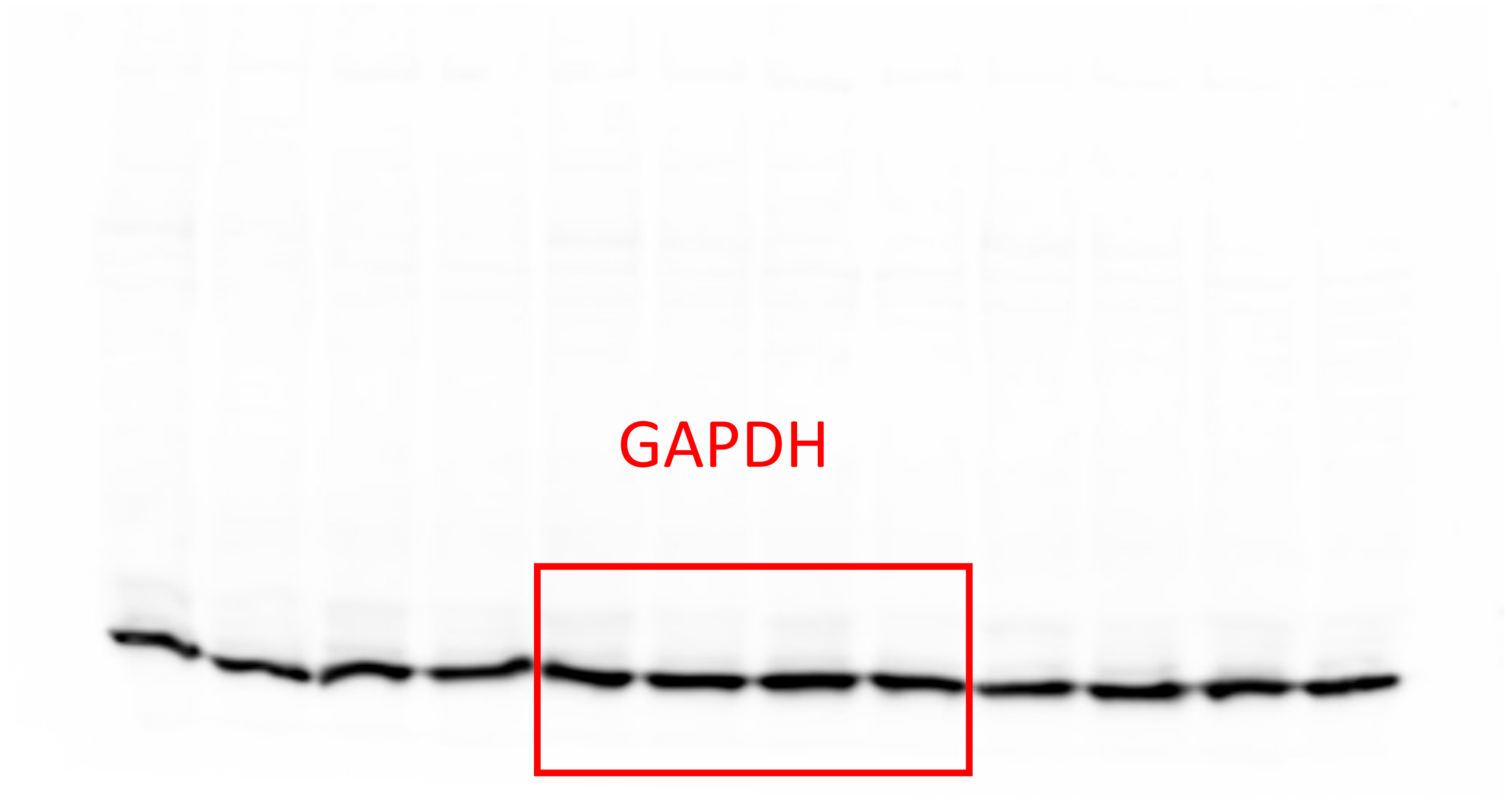

Full unedited gel for Figure 2B – M238

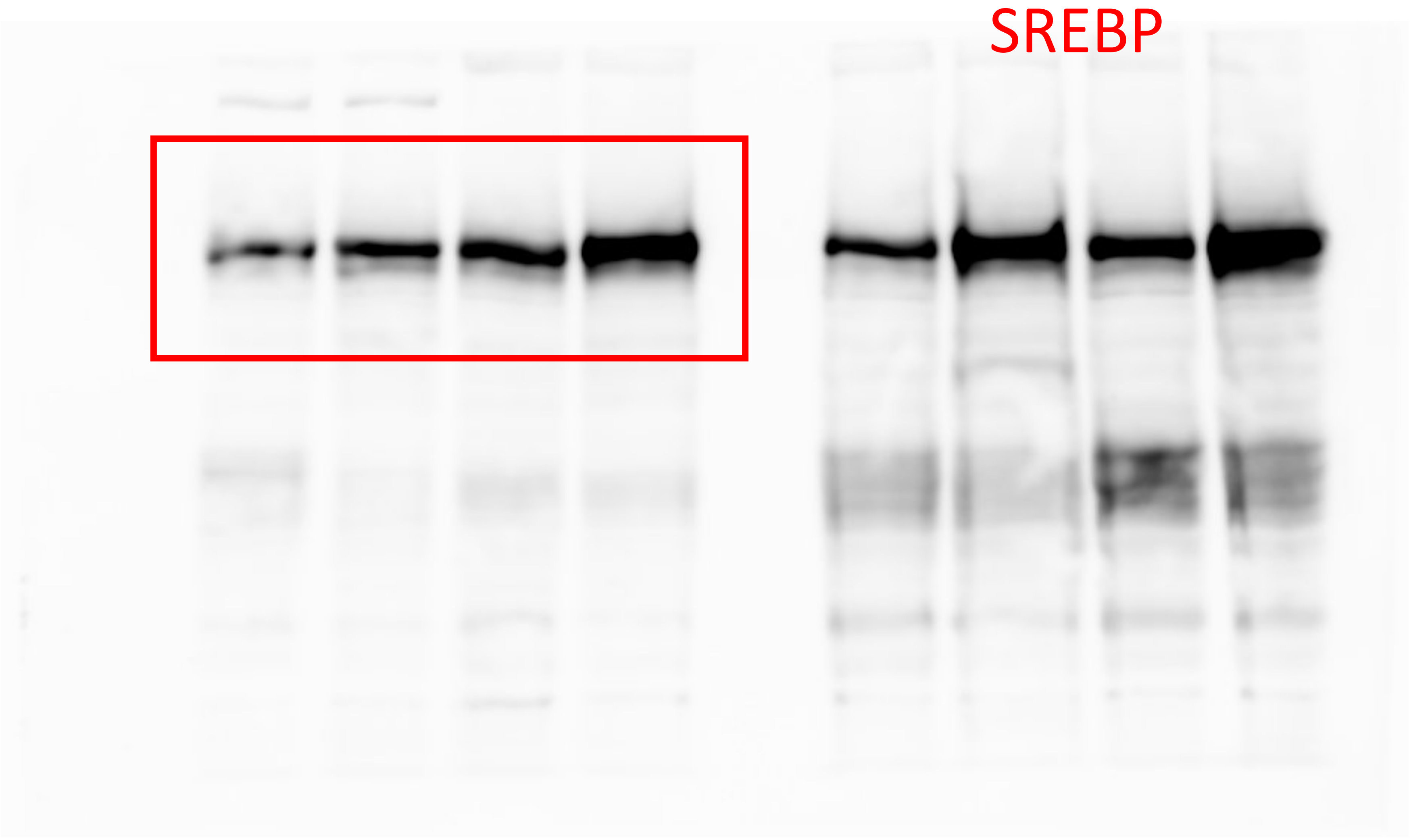

Full unedited gel for Figure 2B – M238

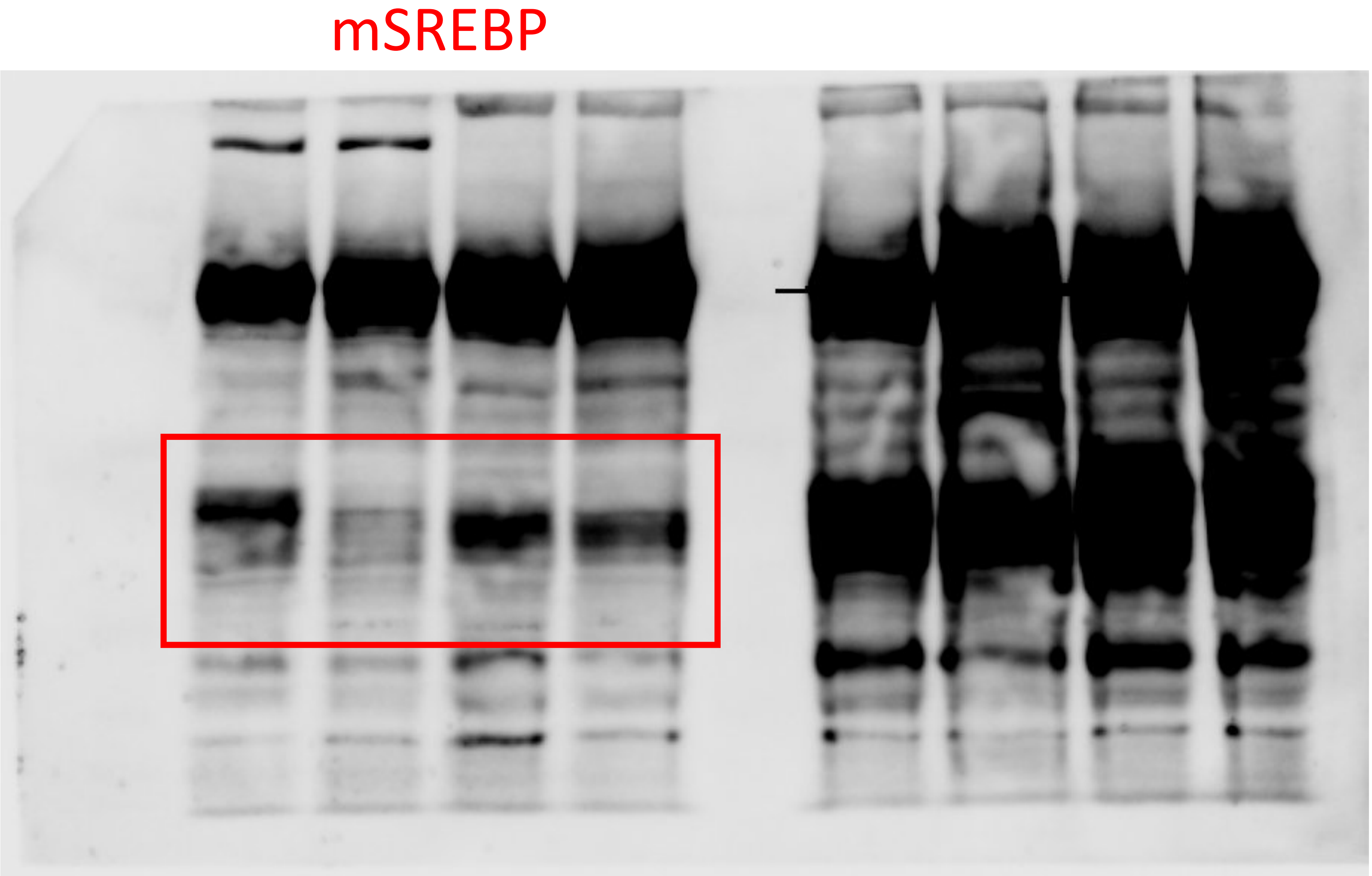

Full unedited gel for Figure 2B – M238

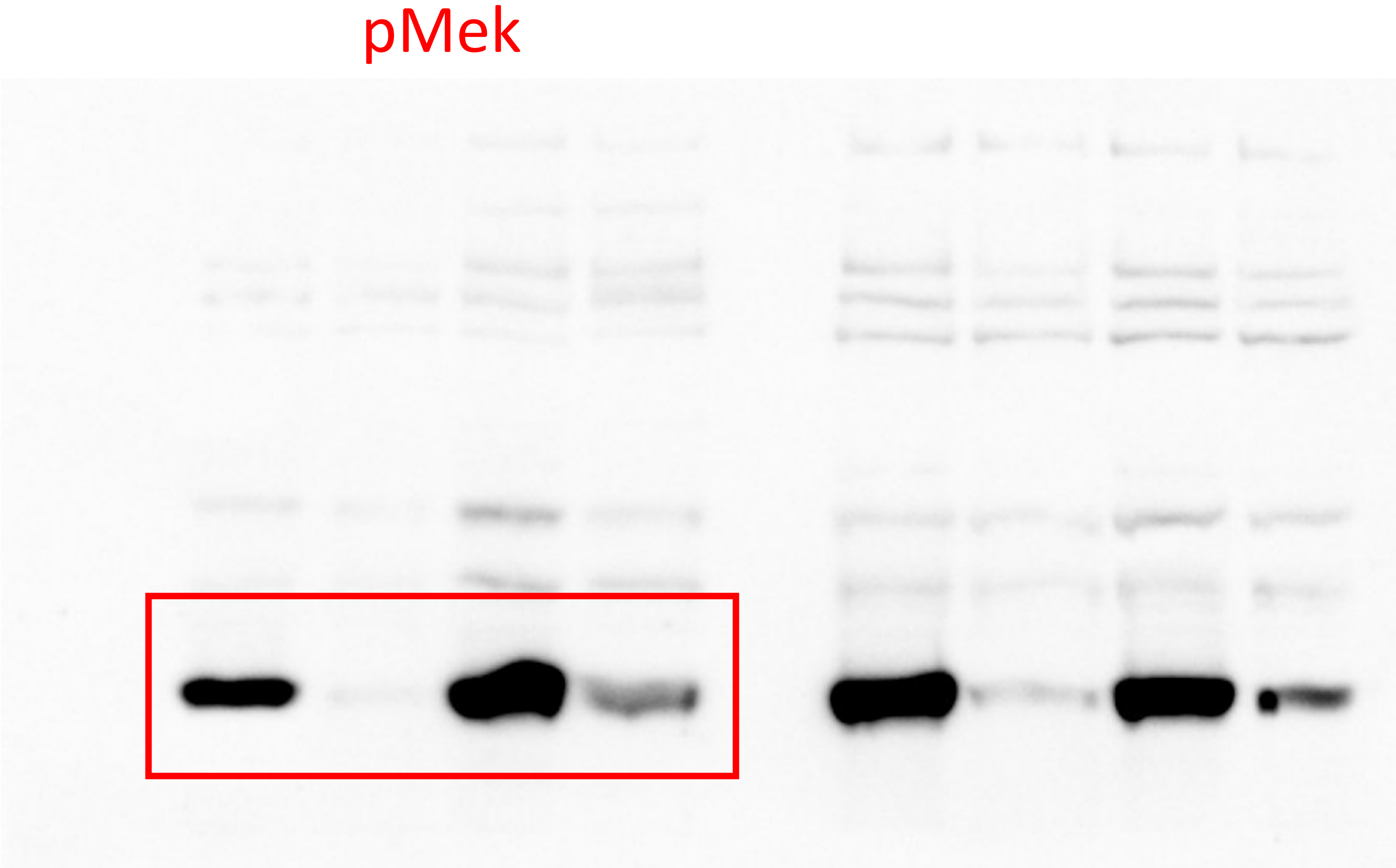

Full unedited gel for Figure 2B – M238

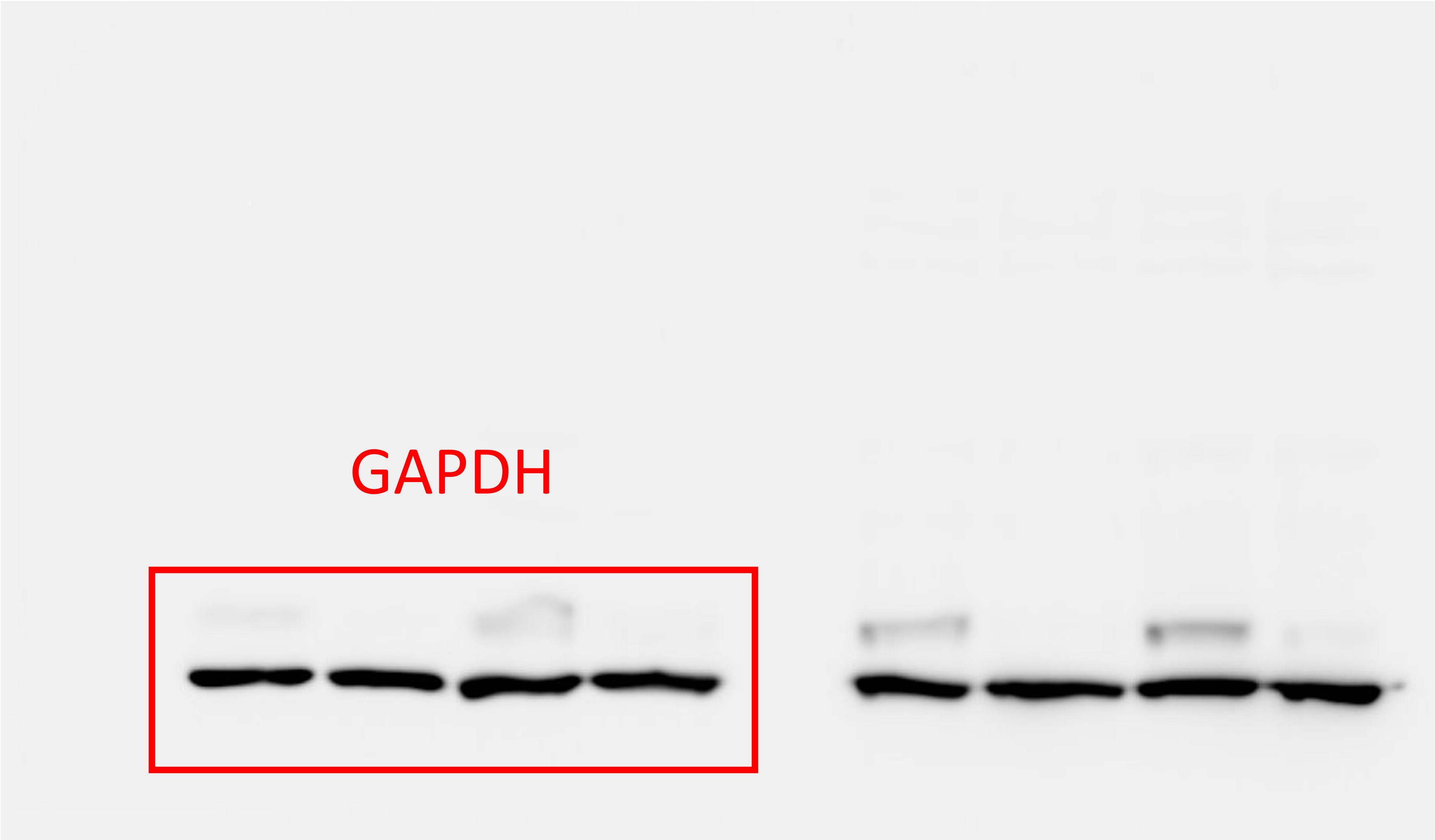

Full unedited gel for Figure 2B – M249

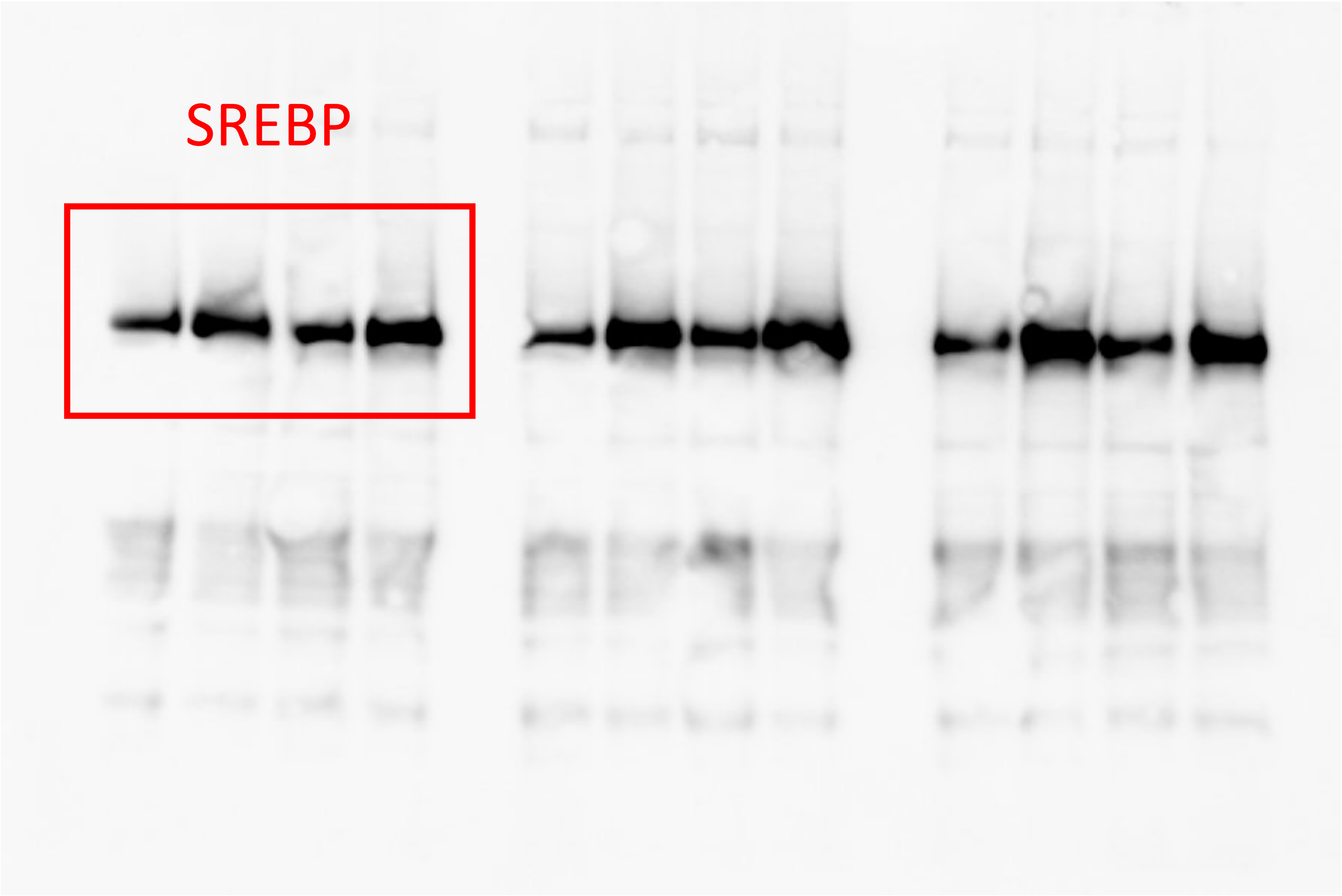

Full unedited gel for Figure 2B – M249

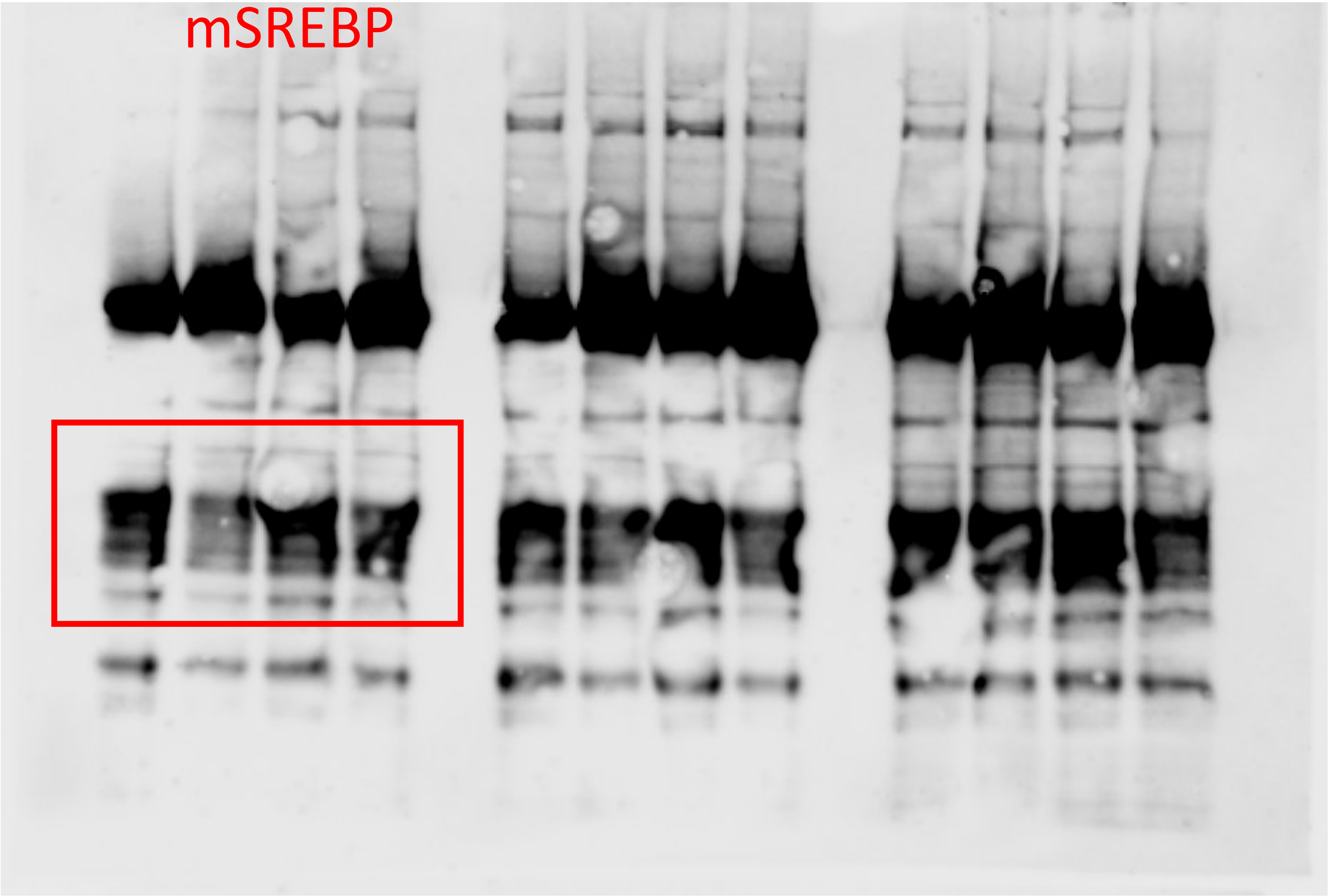

Full unedited gel for Figure 2B – M249

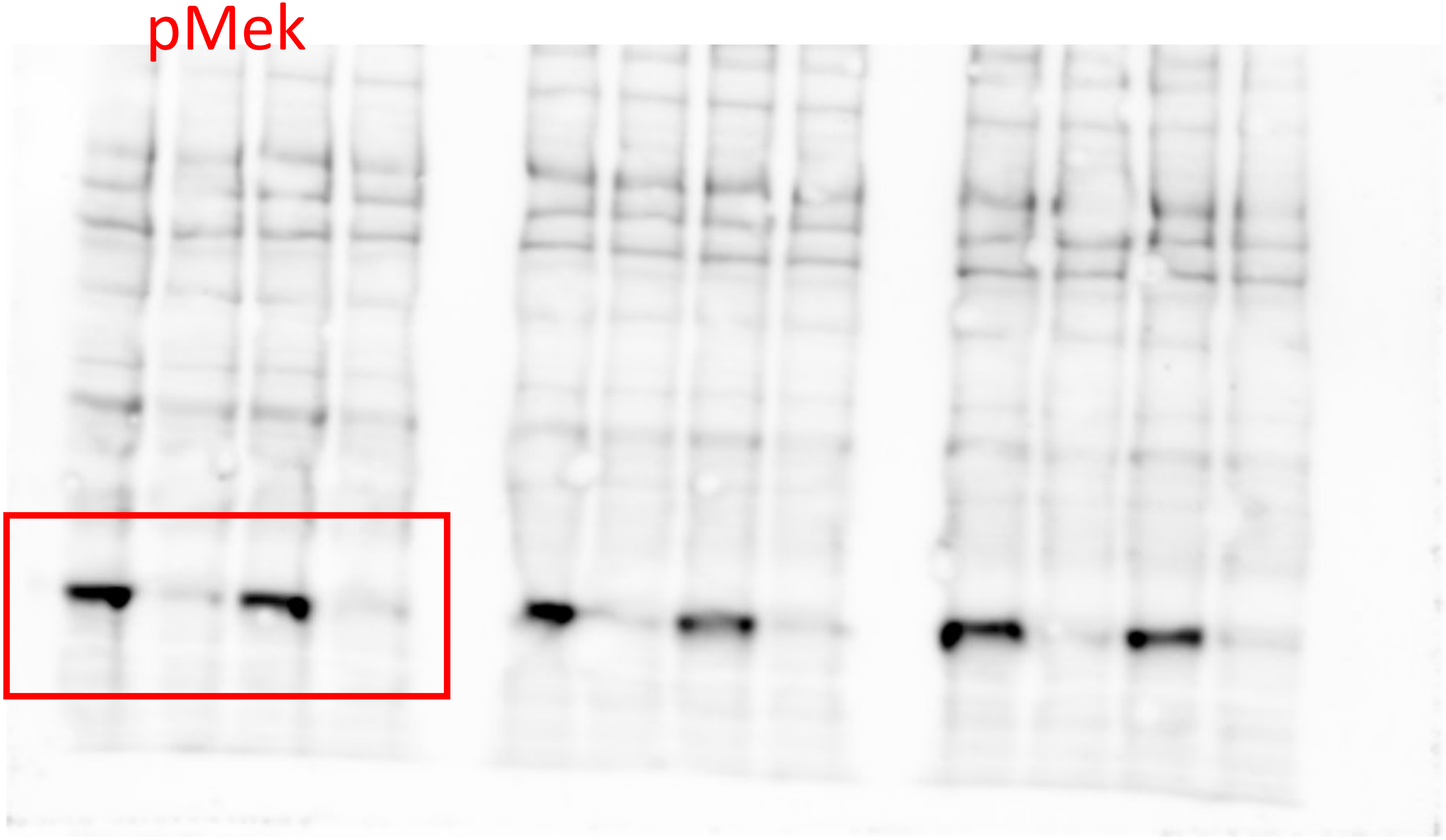

Full unedited gel for Figure 2B – M249

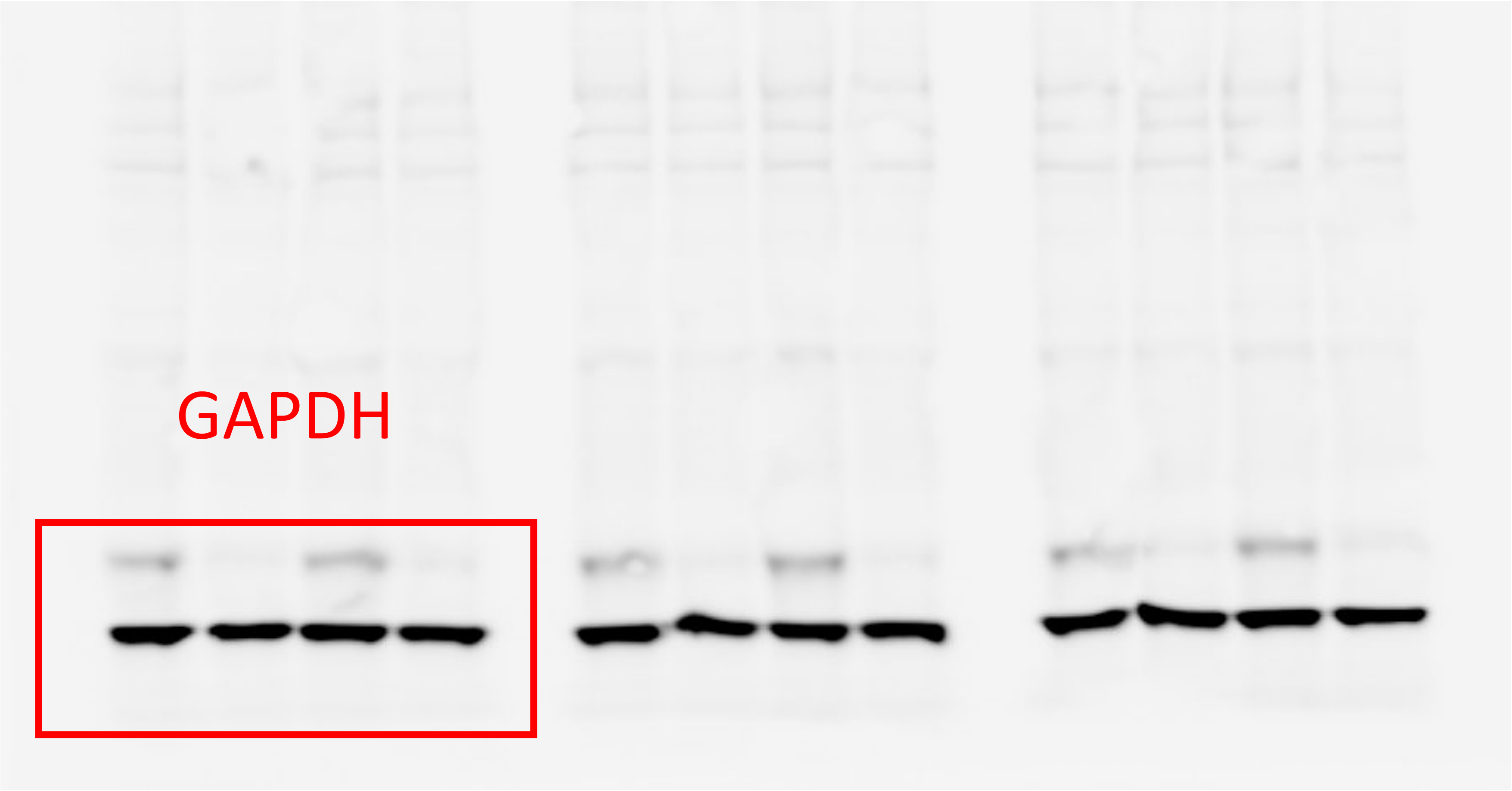

Full unedited gel for Figure 2B – A375 + FLCM

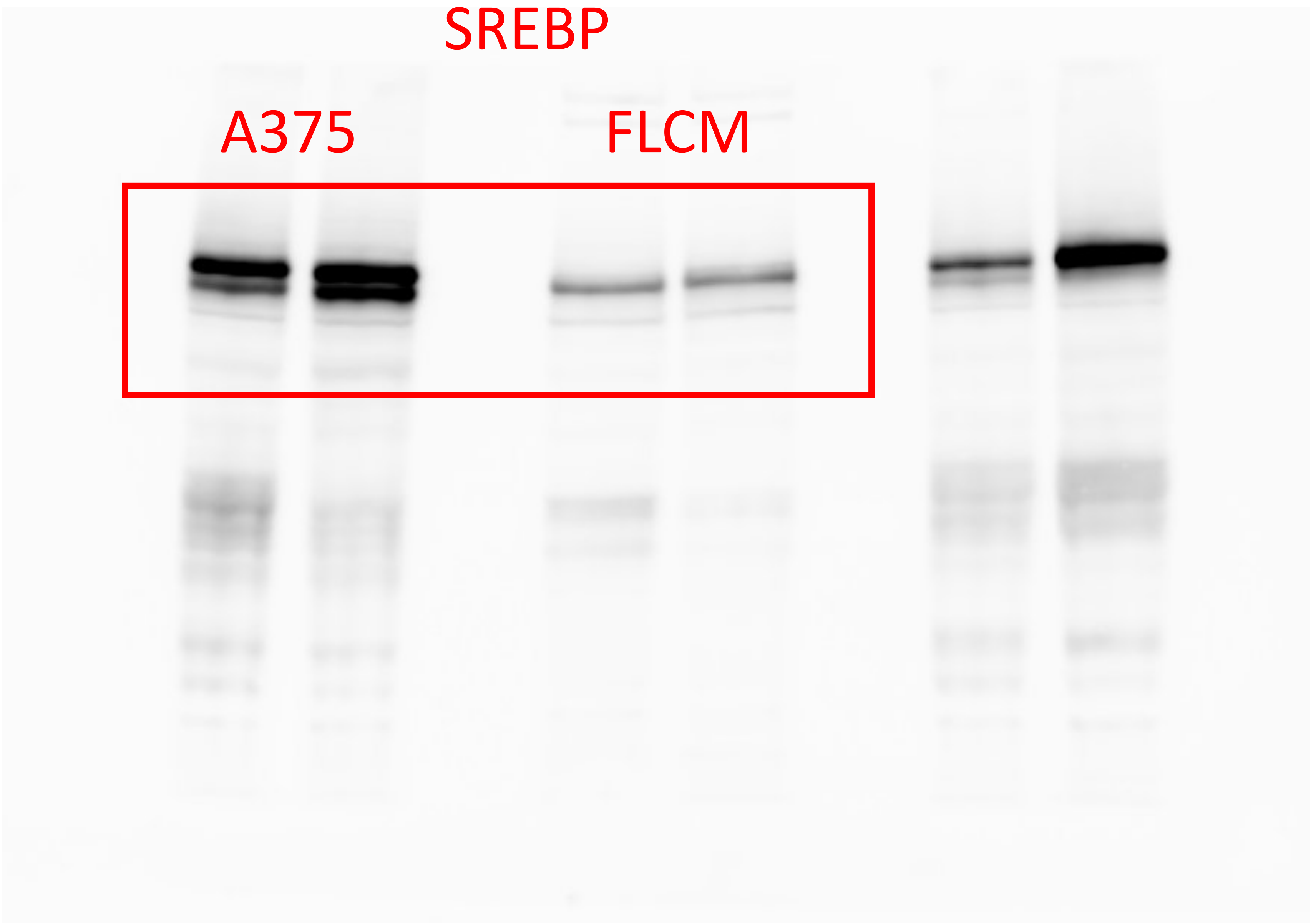

Full unedited gel for Figure 2B – A375 + FLCM

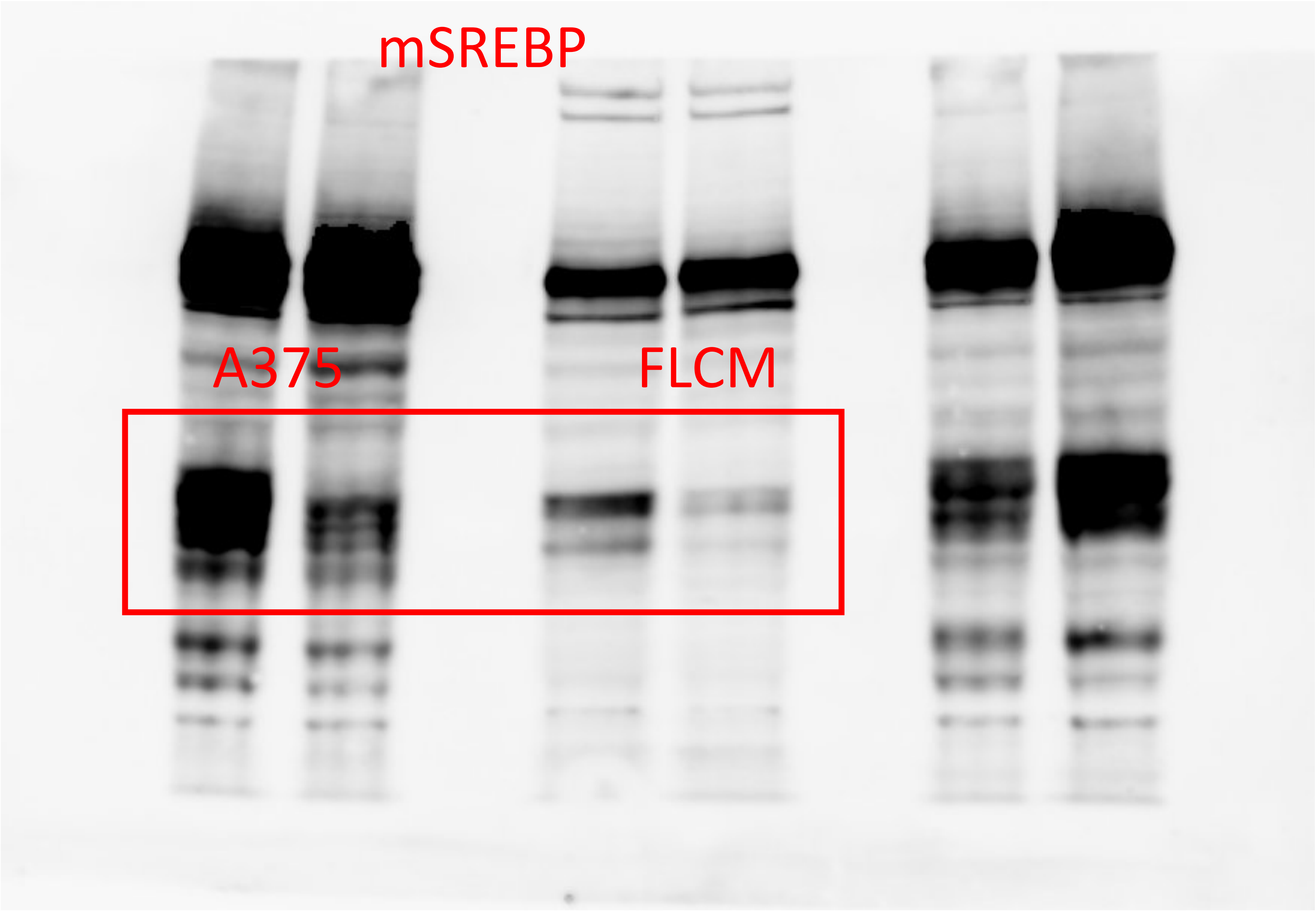

Full unedited gel for Figure 2B – A375 + FLCM

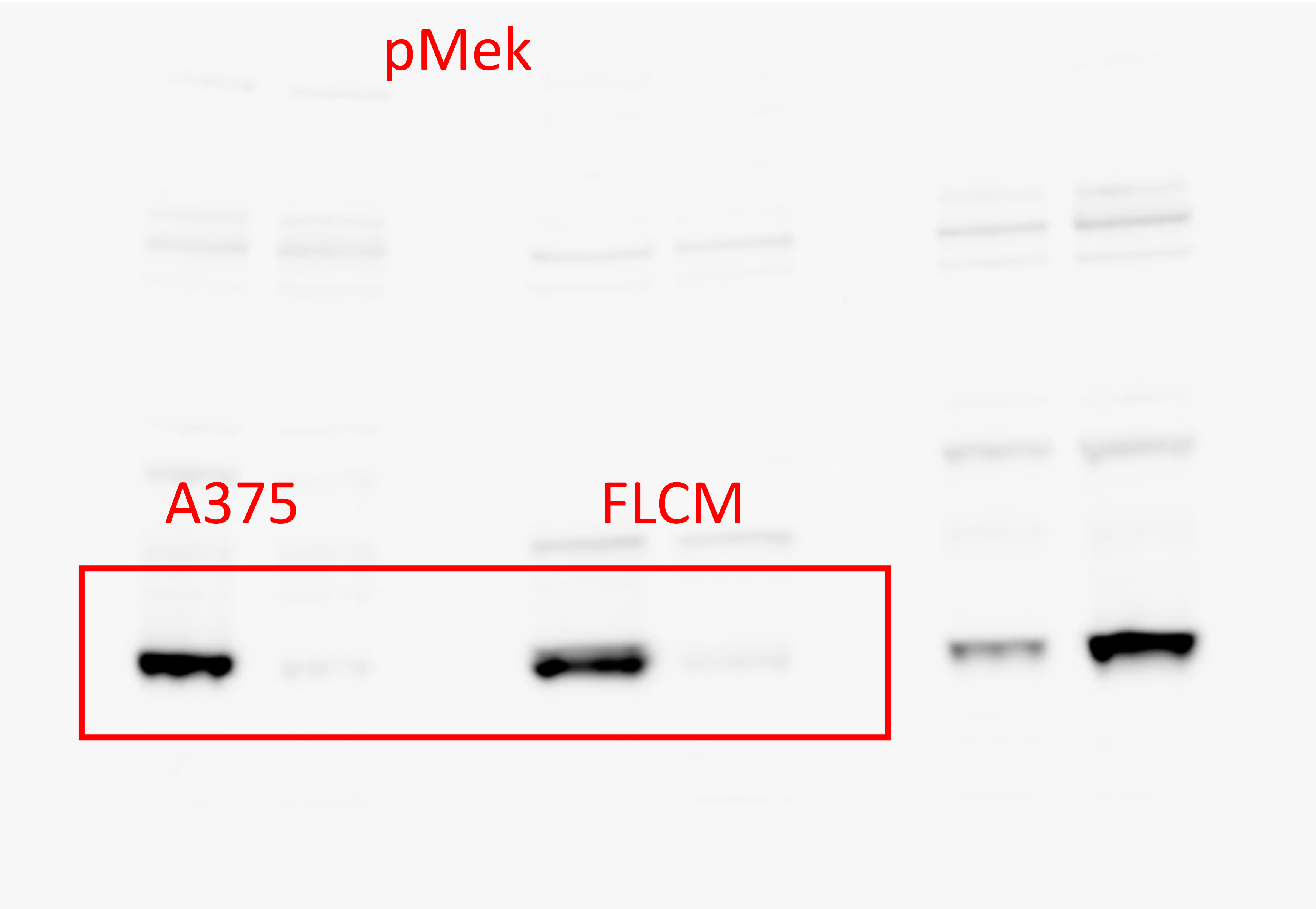

Full unedited gel for Figure 2B – A375 + FLCM

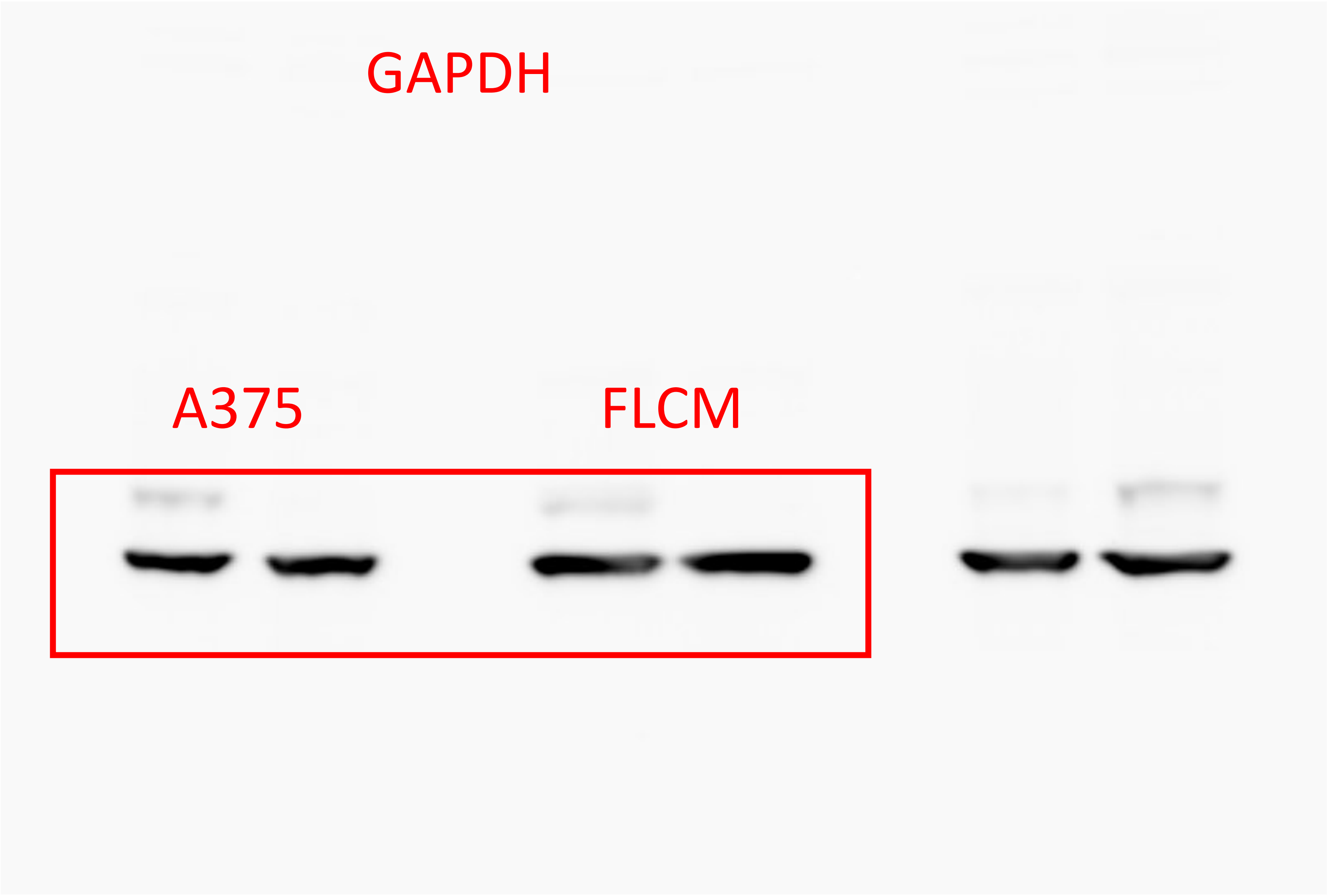

Full unedited gel for Figure 2B – NNHEM

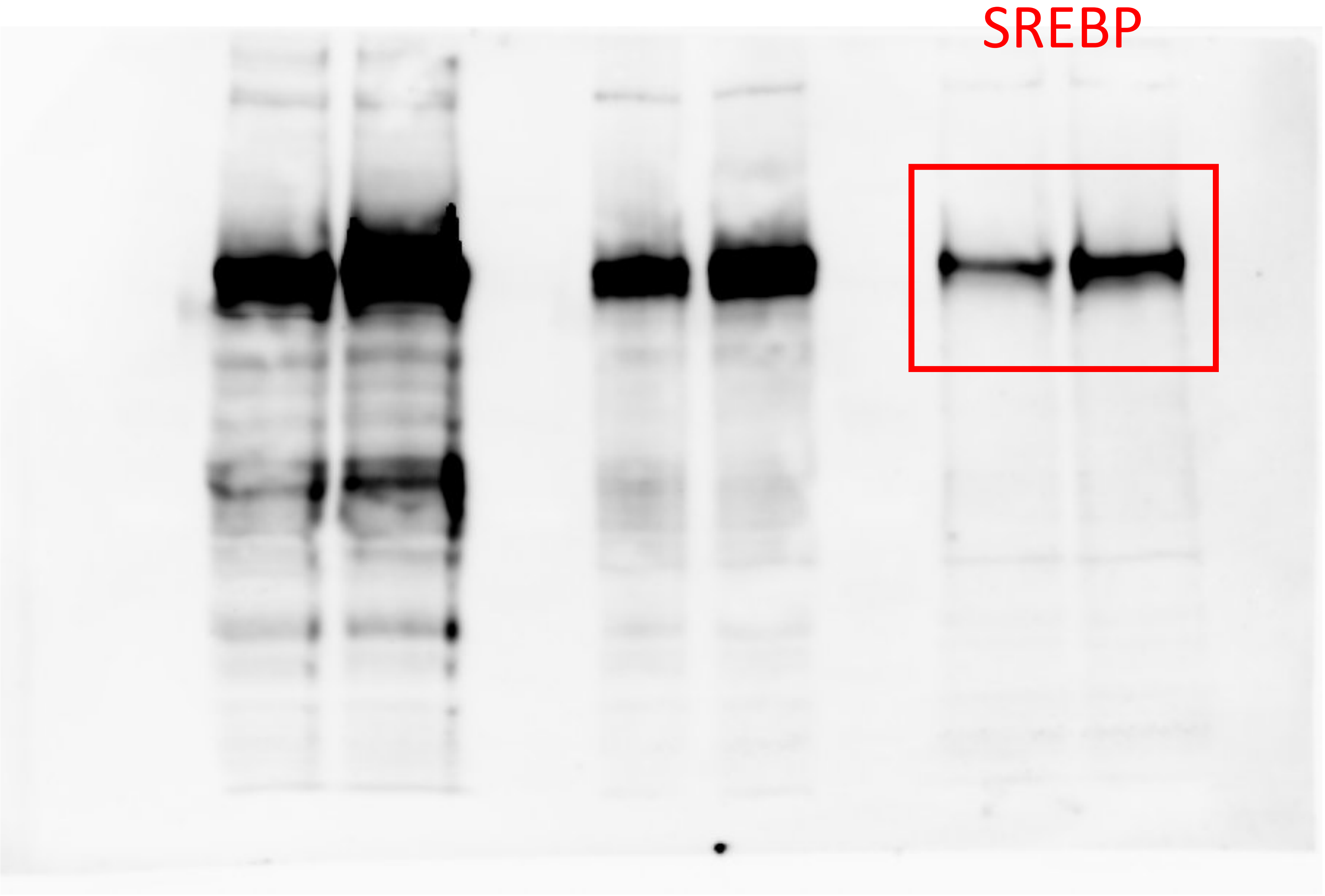

Full unedited gel for Figure 2B – A375 + FLCM

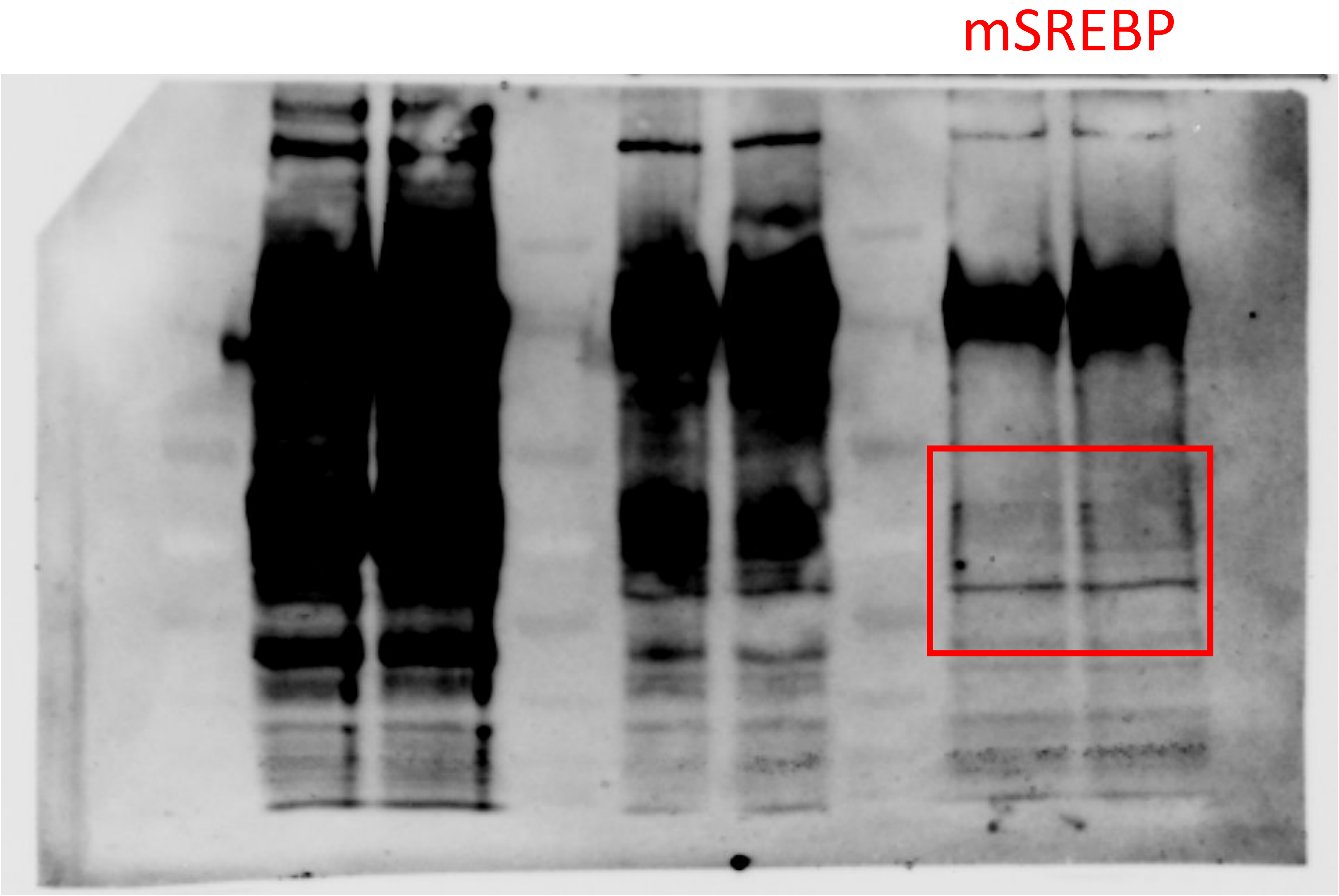

Full unedited gel for Figure 2B – A375 + FLCM

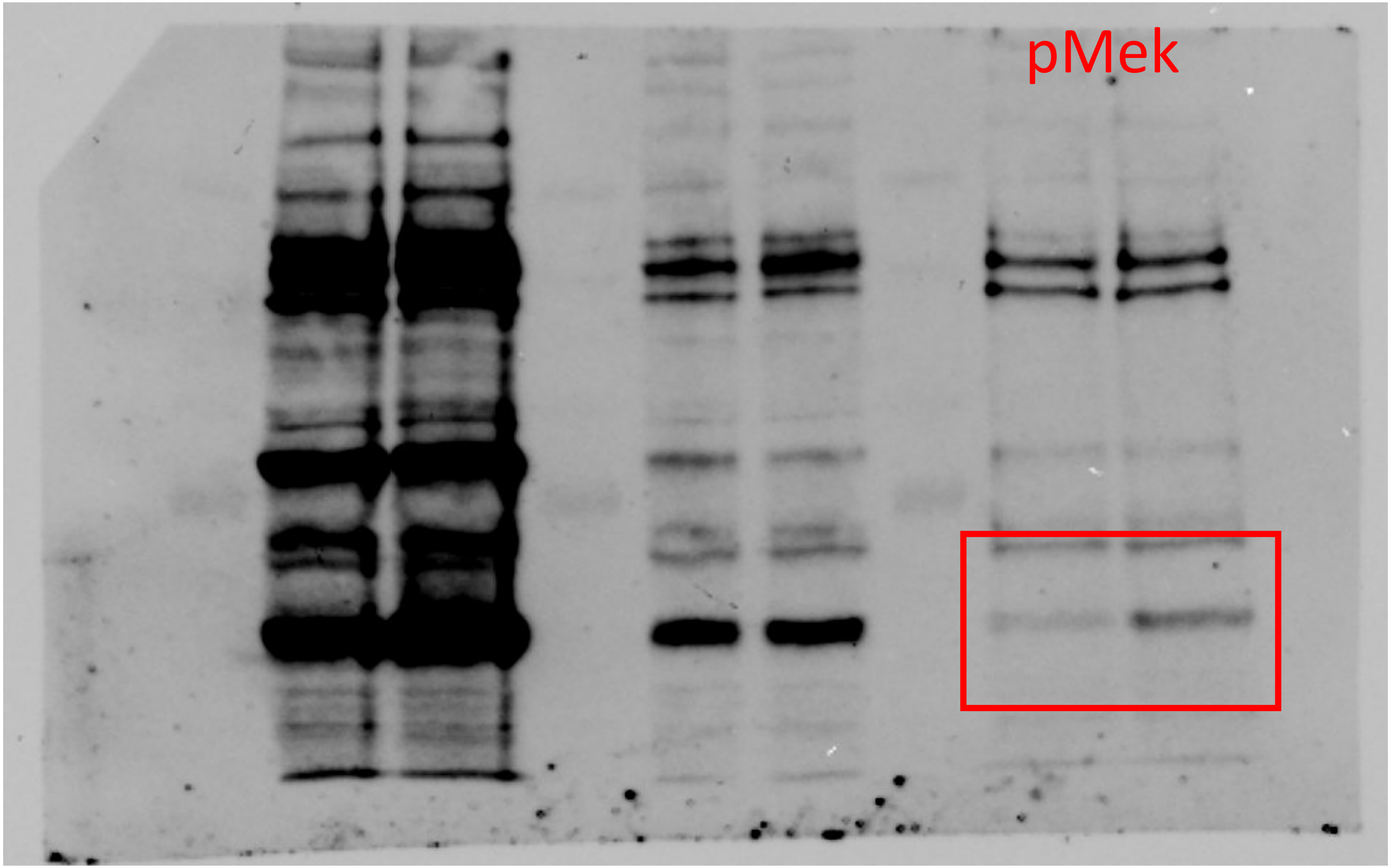

Full unedited gel for Figure 2B – A375 + FLCM

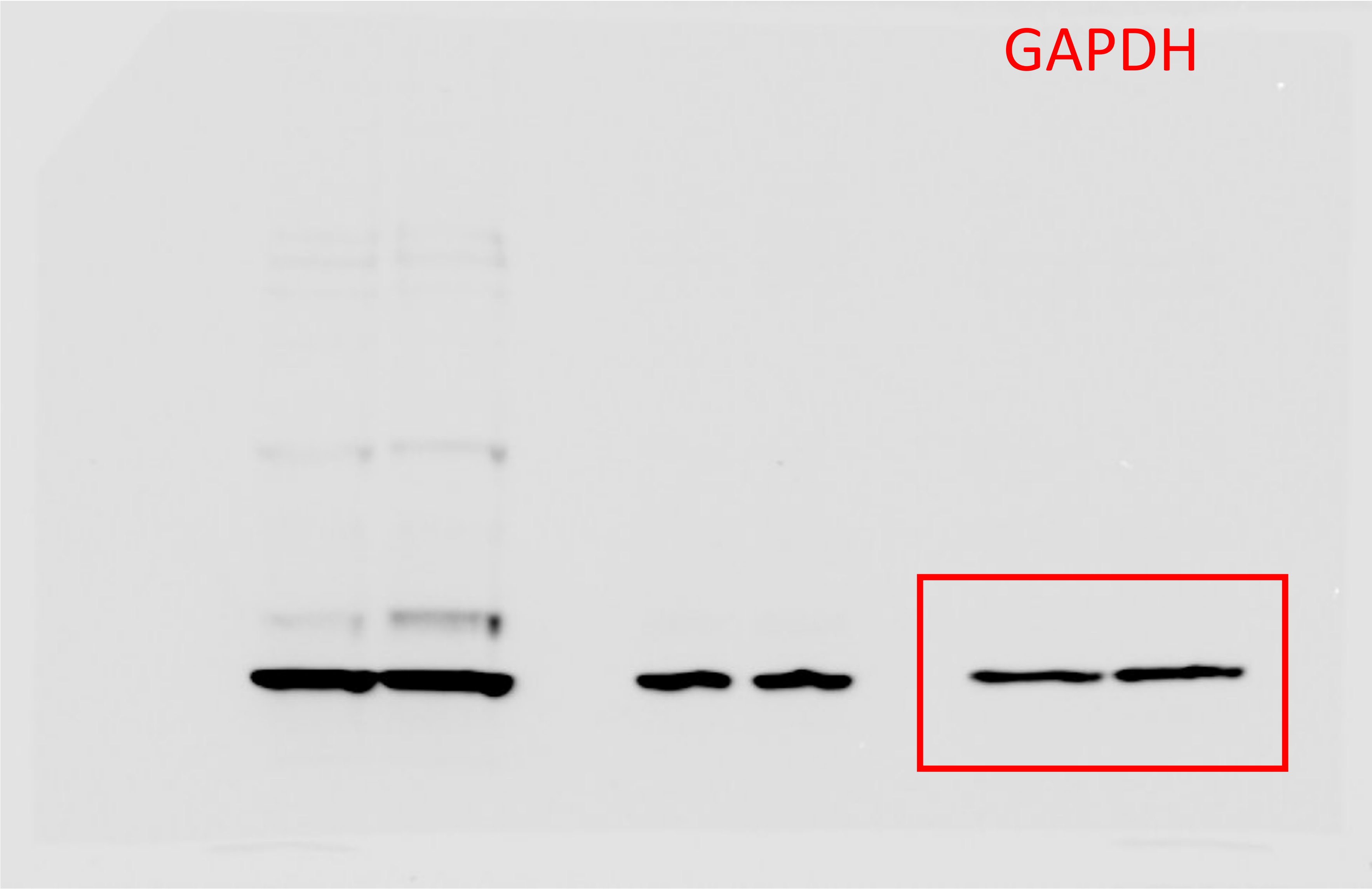

Full unedited gel for Figure 2C

Full unedited gel for Figure 2C - M202

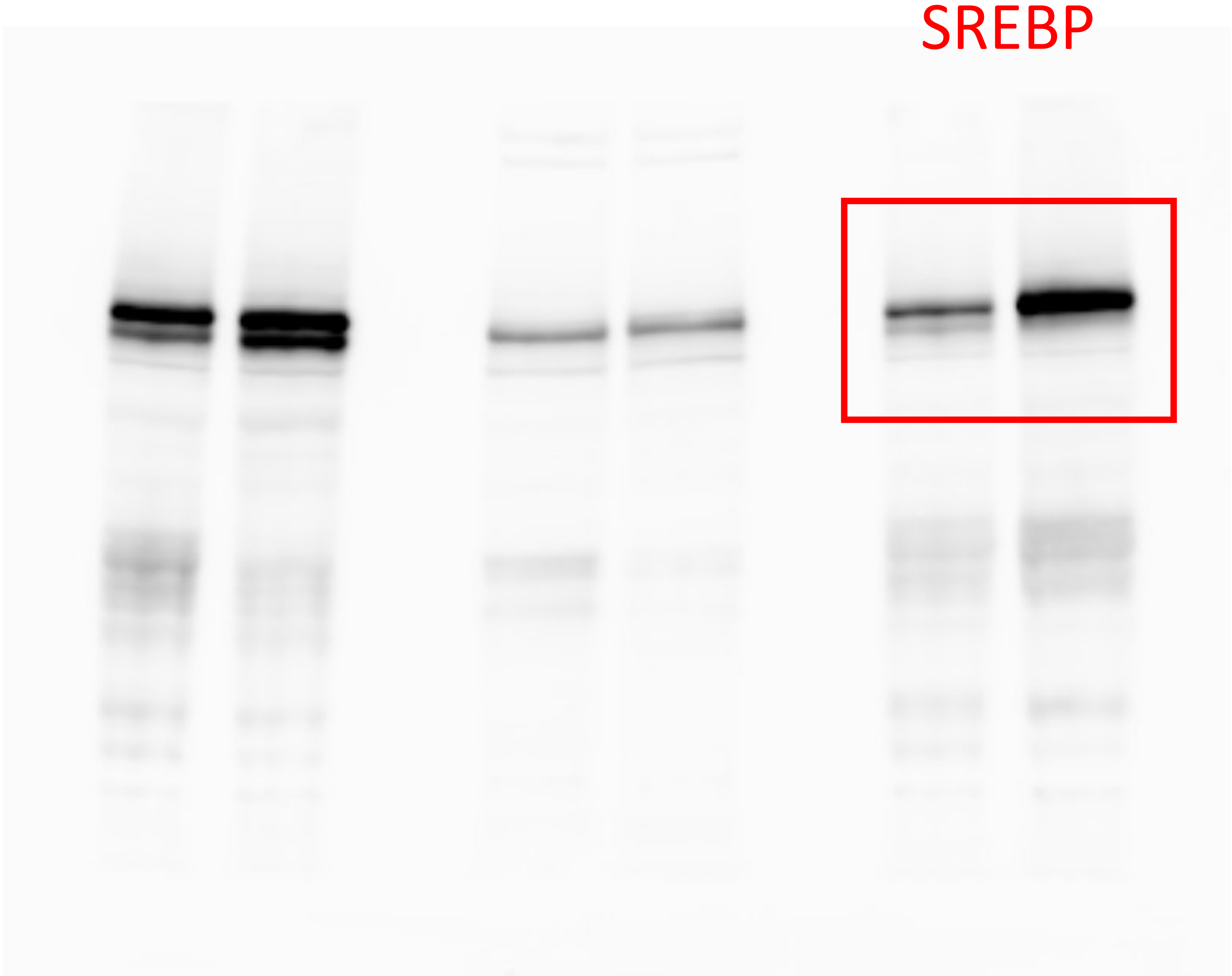

Full unedited gel for Figure 2C - M202

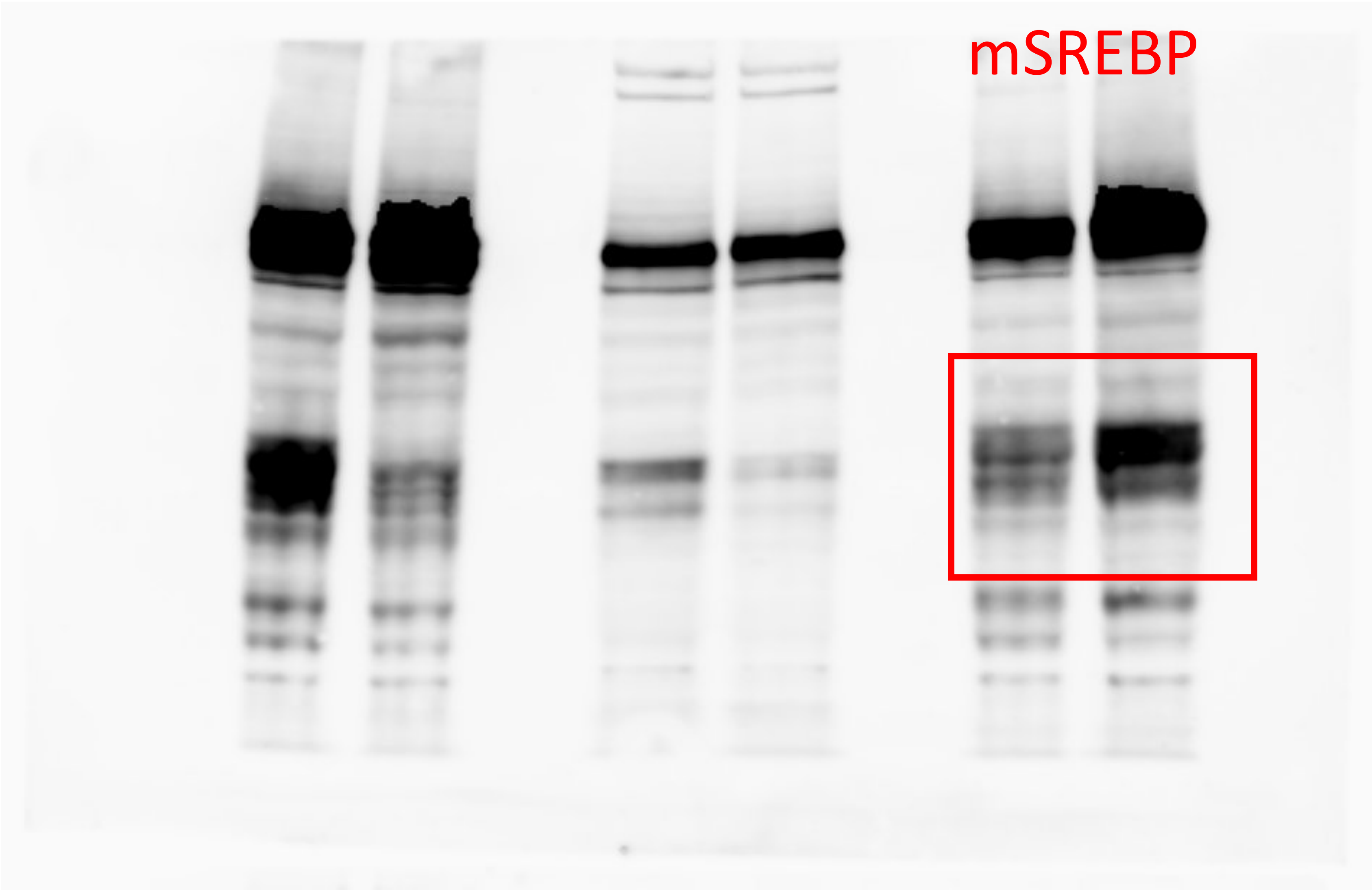

Full unedited gel for Figure 2C - M202

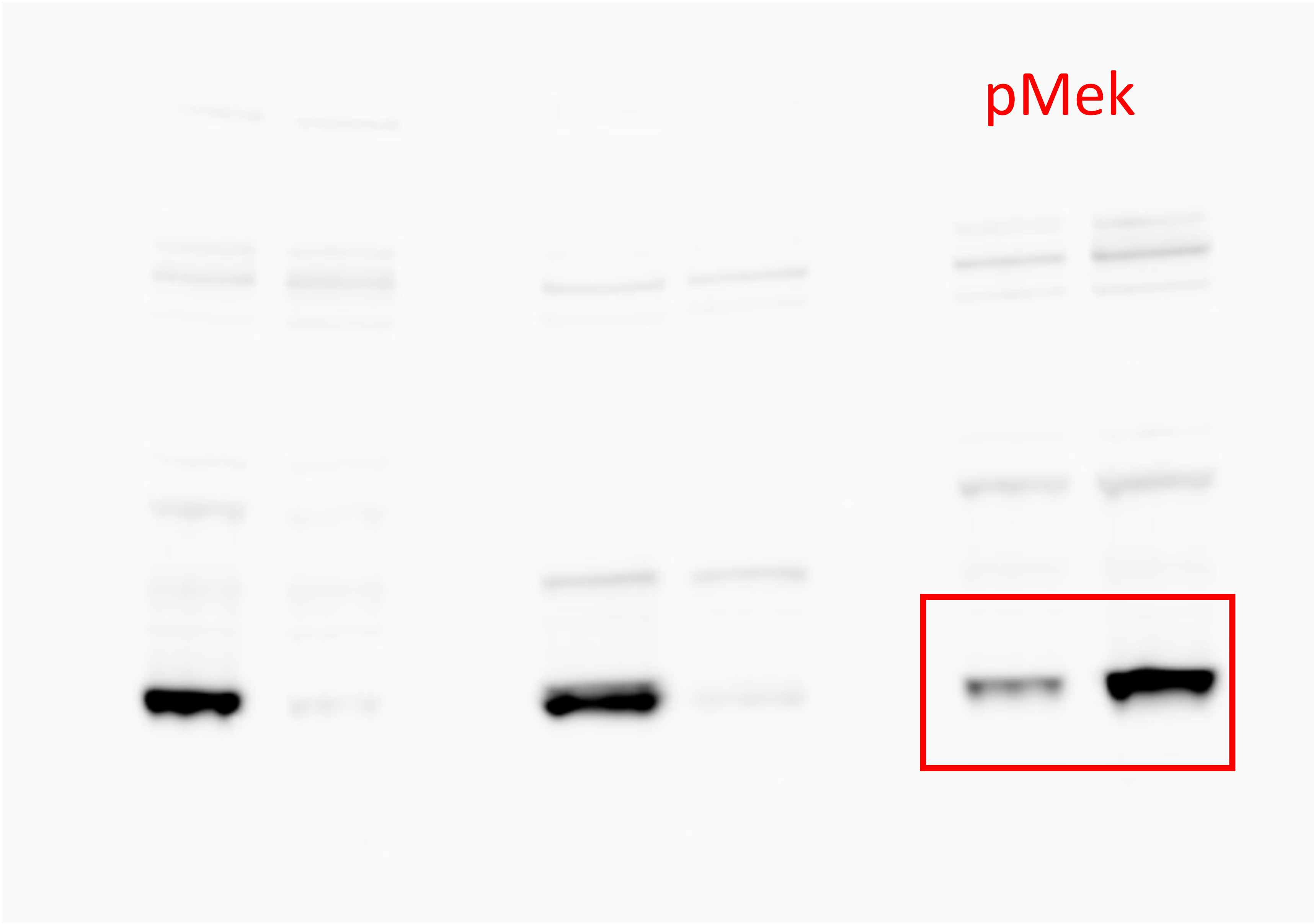

Full unedited gel for Figure 2C - M202

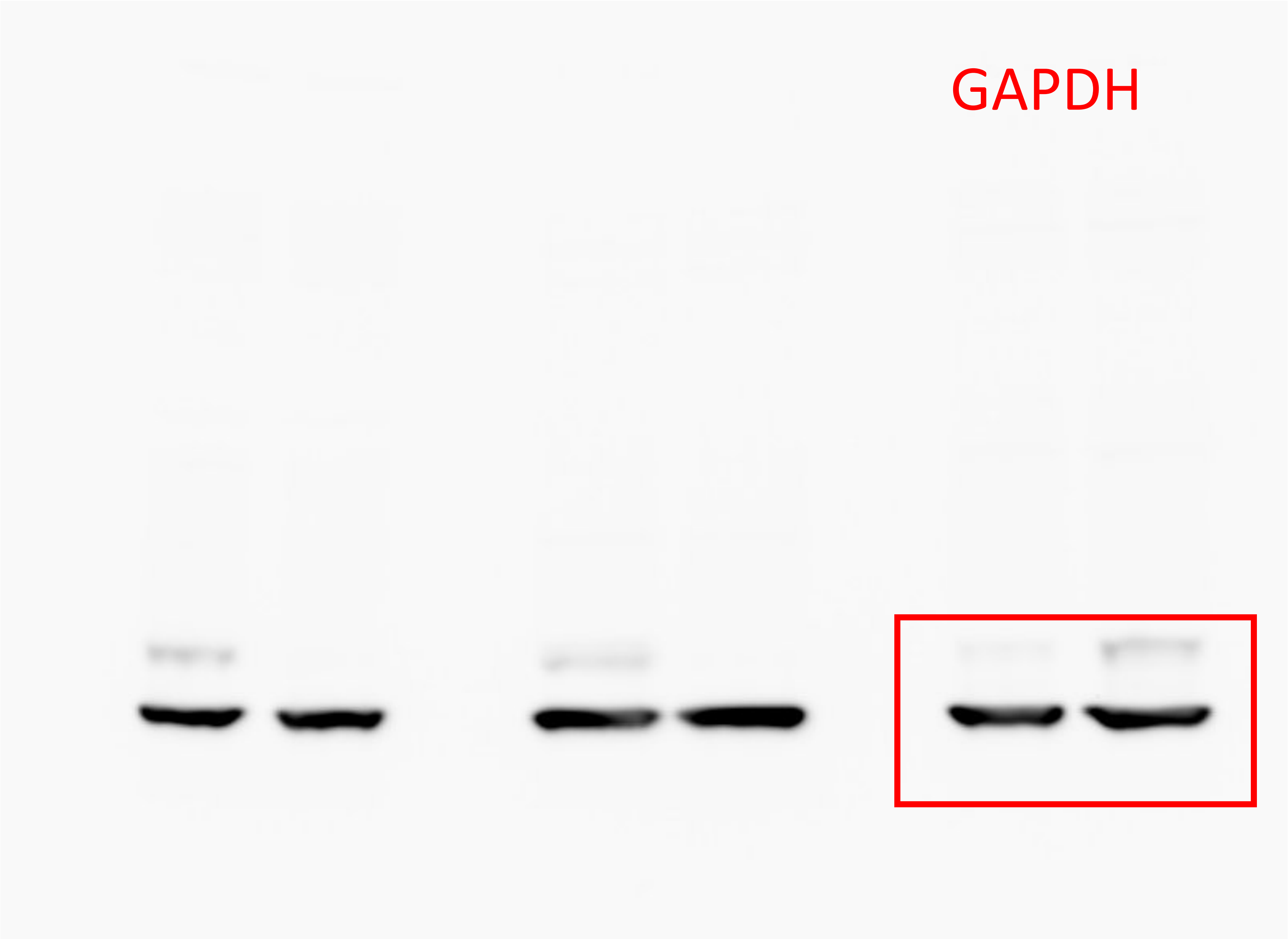

Full unedited gel for Figure 2C - M207

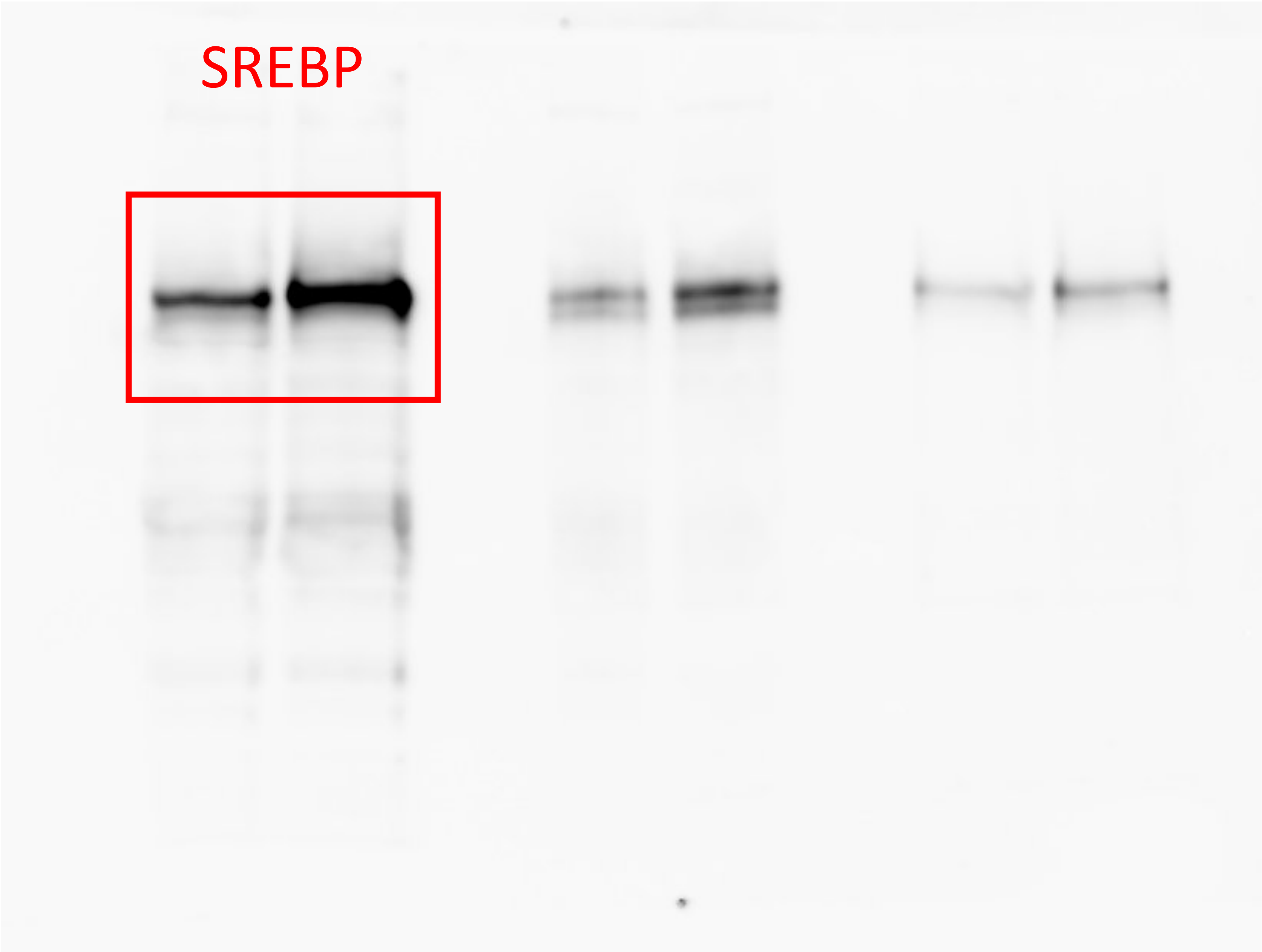

Full unedited gel for Figure 2C - M207

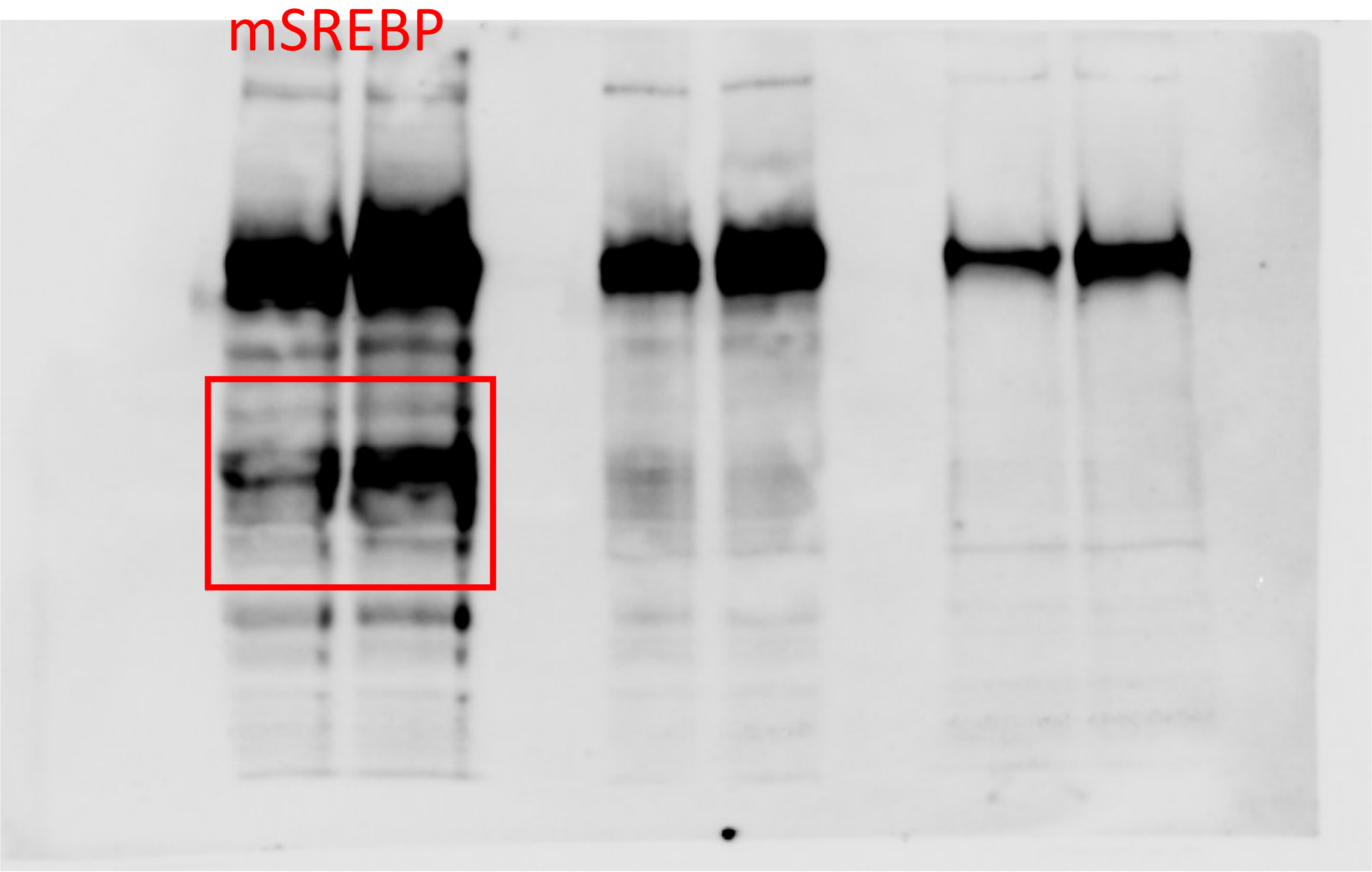

Full unedited gel for Figure 2C - M207

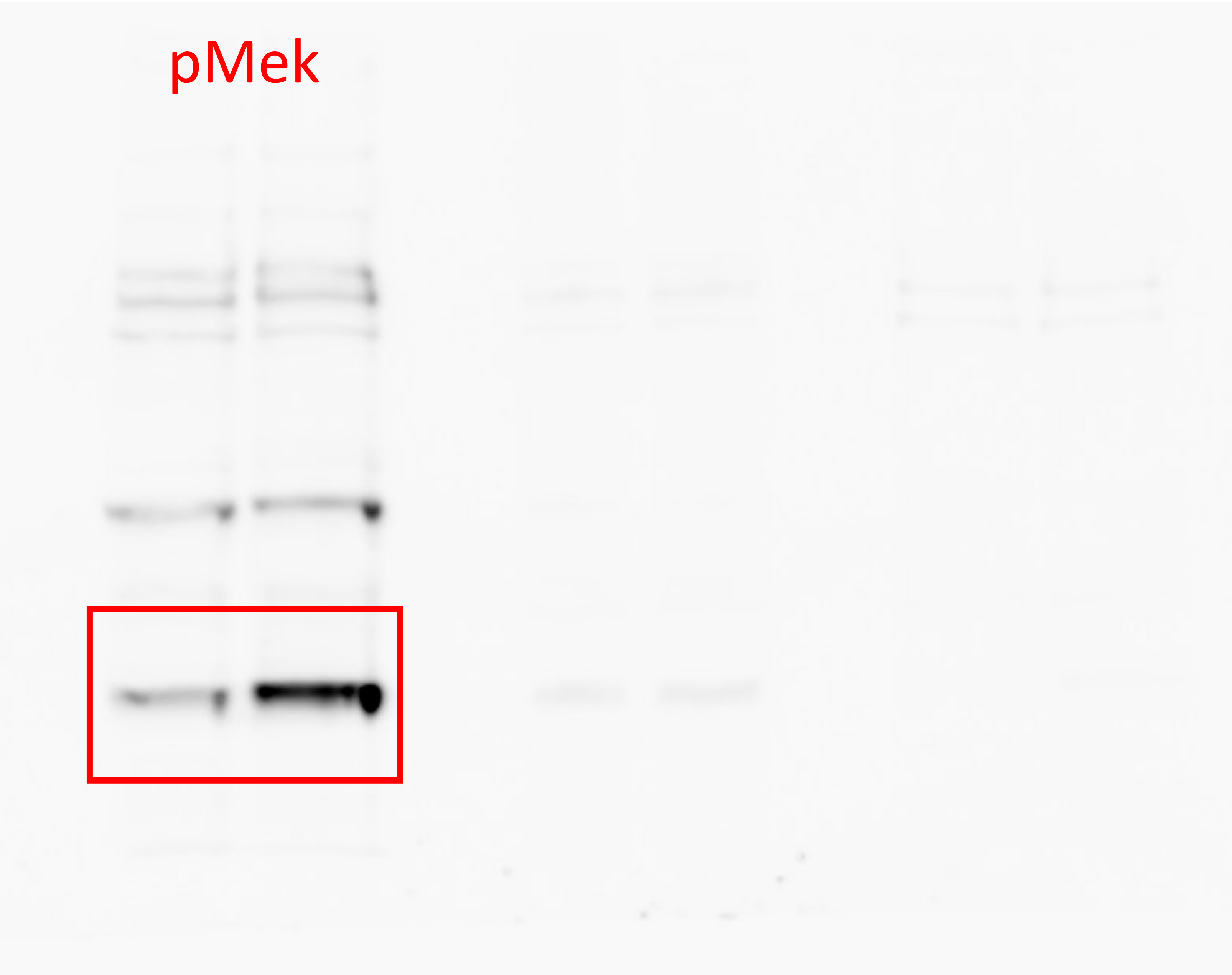

Full unedited gel for Figure 2C - M207

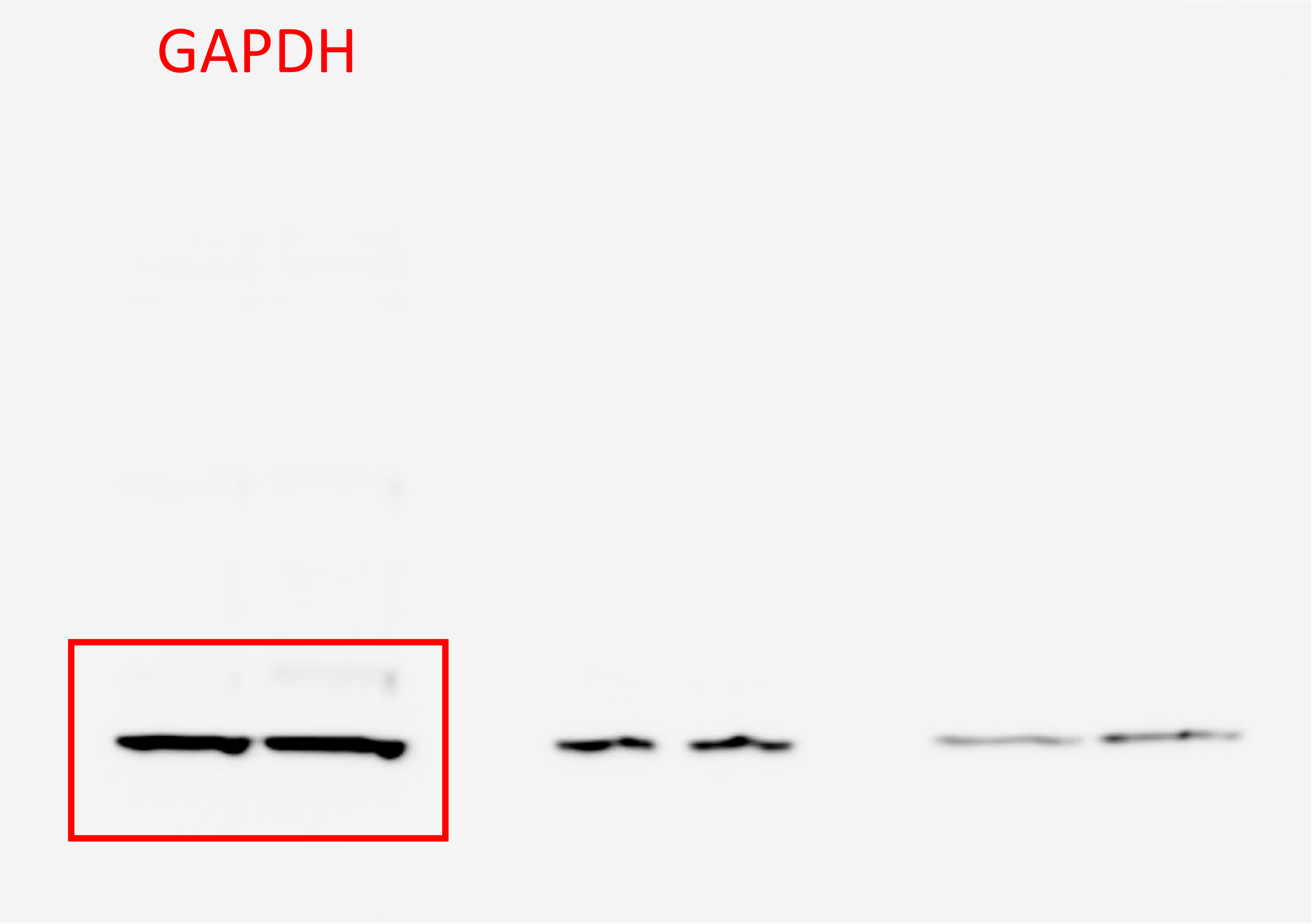

Full unedited gel for Figure 2D

Full unedited gel for Figure 2D - M202

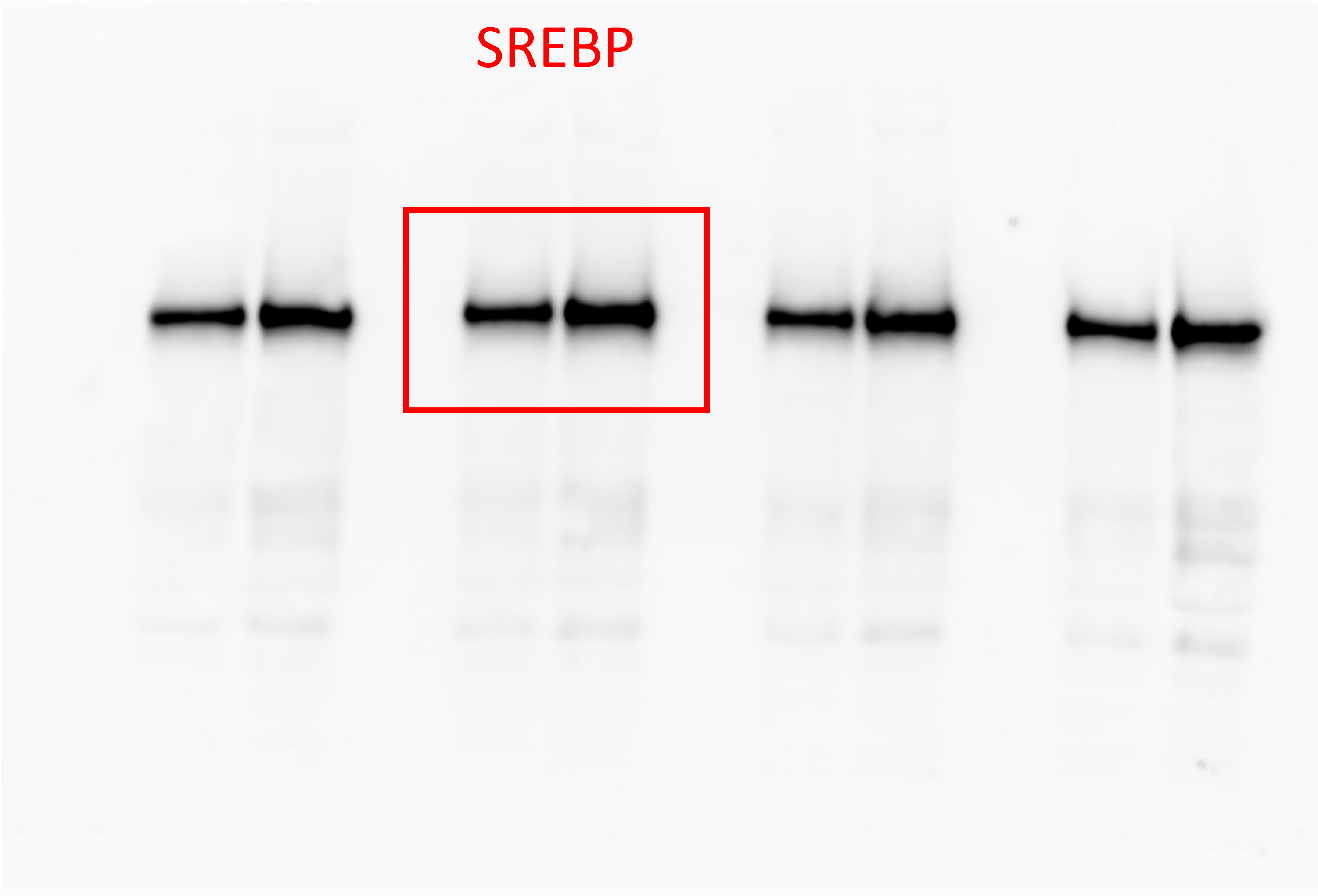

Full unedited gel for Figure 2D - M202

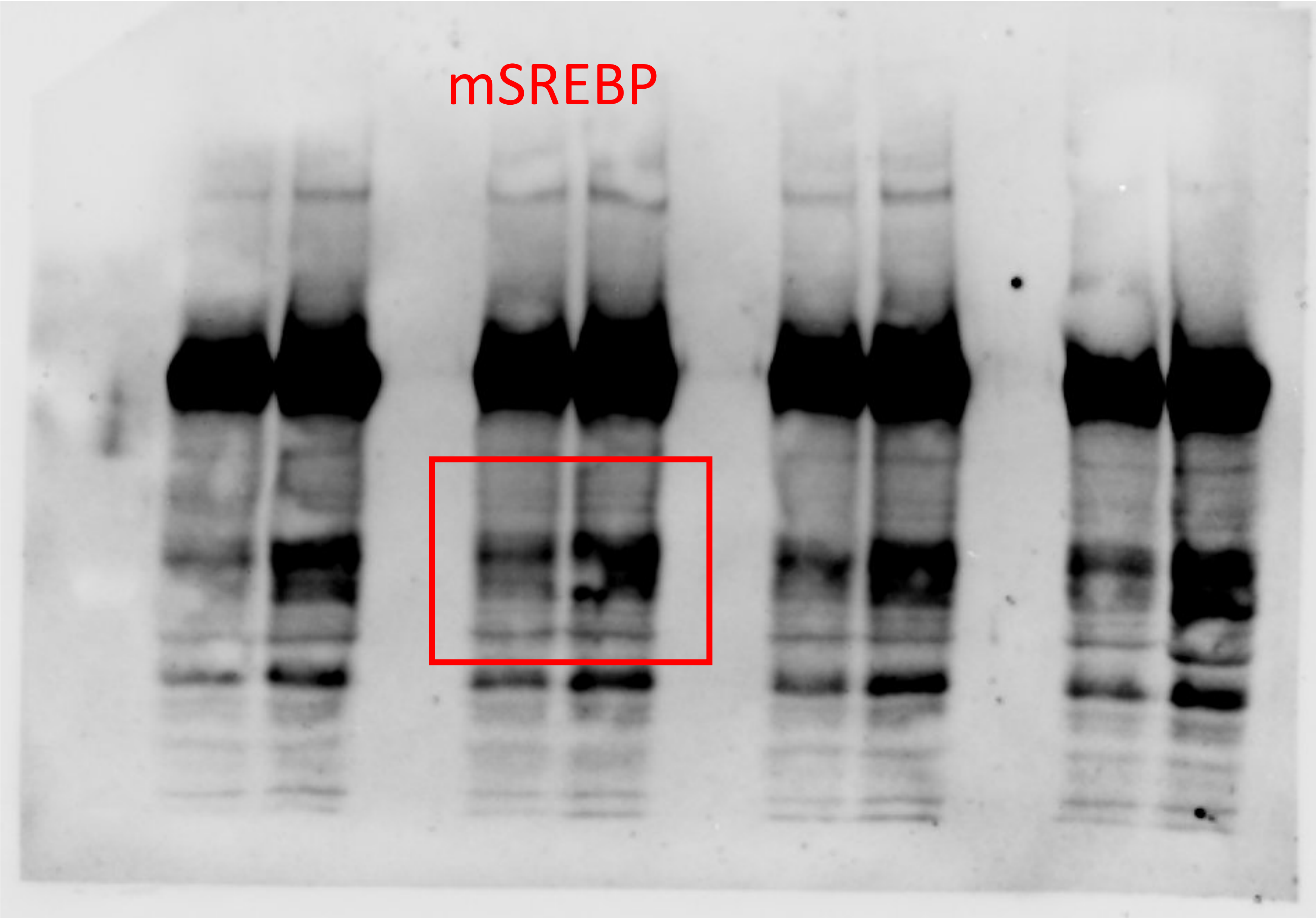

Full unedited gel for Figure 2D - M202

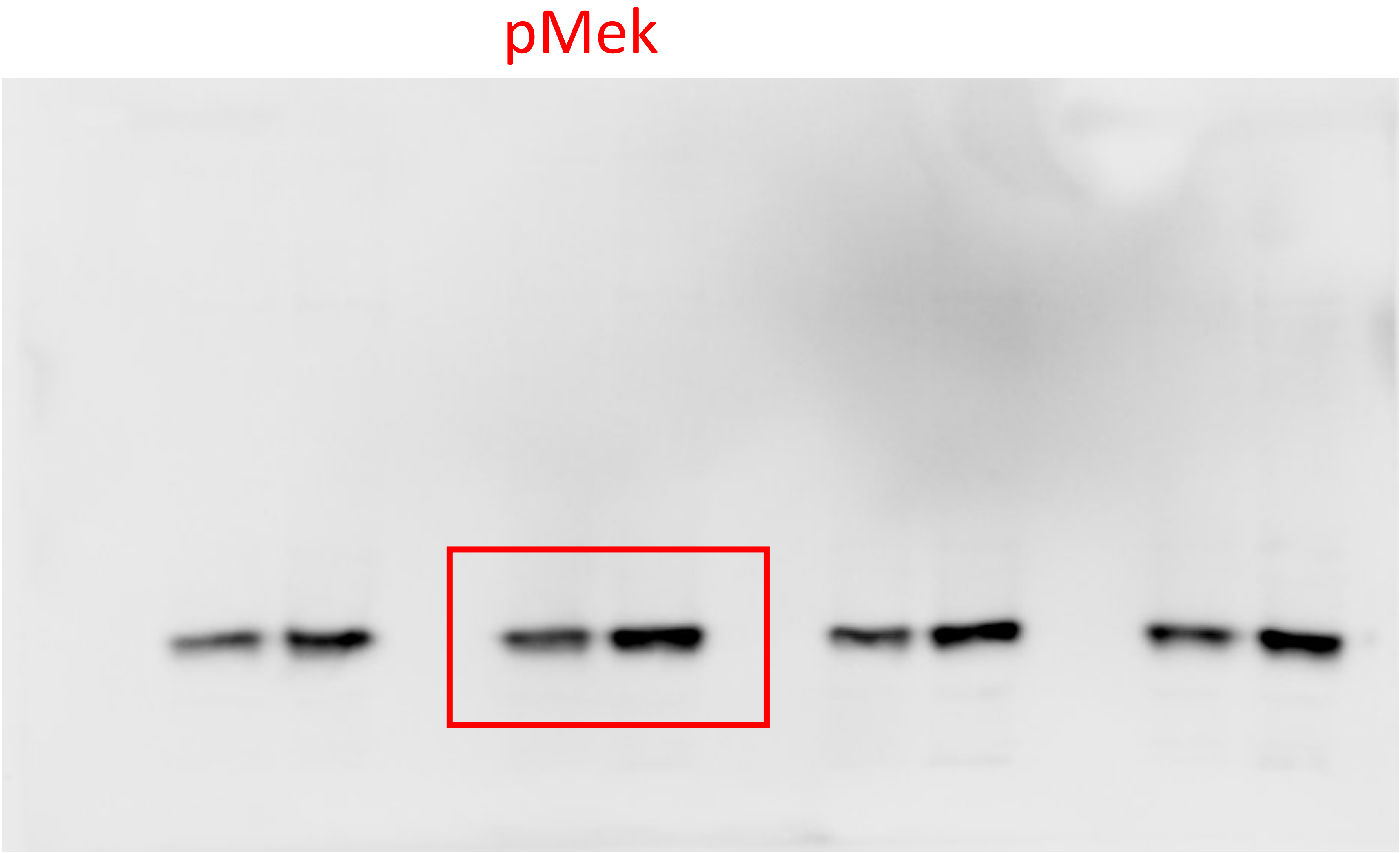

Full unedited gel for Figure 2D - M202

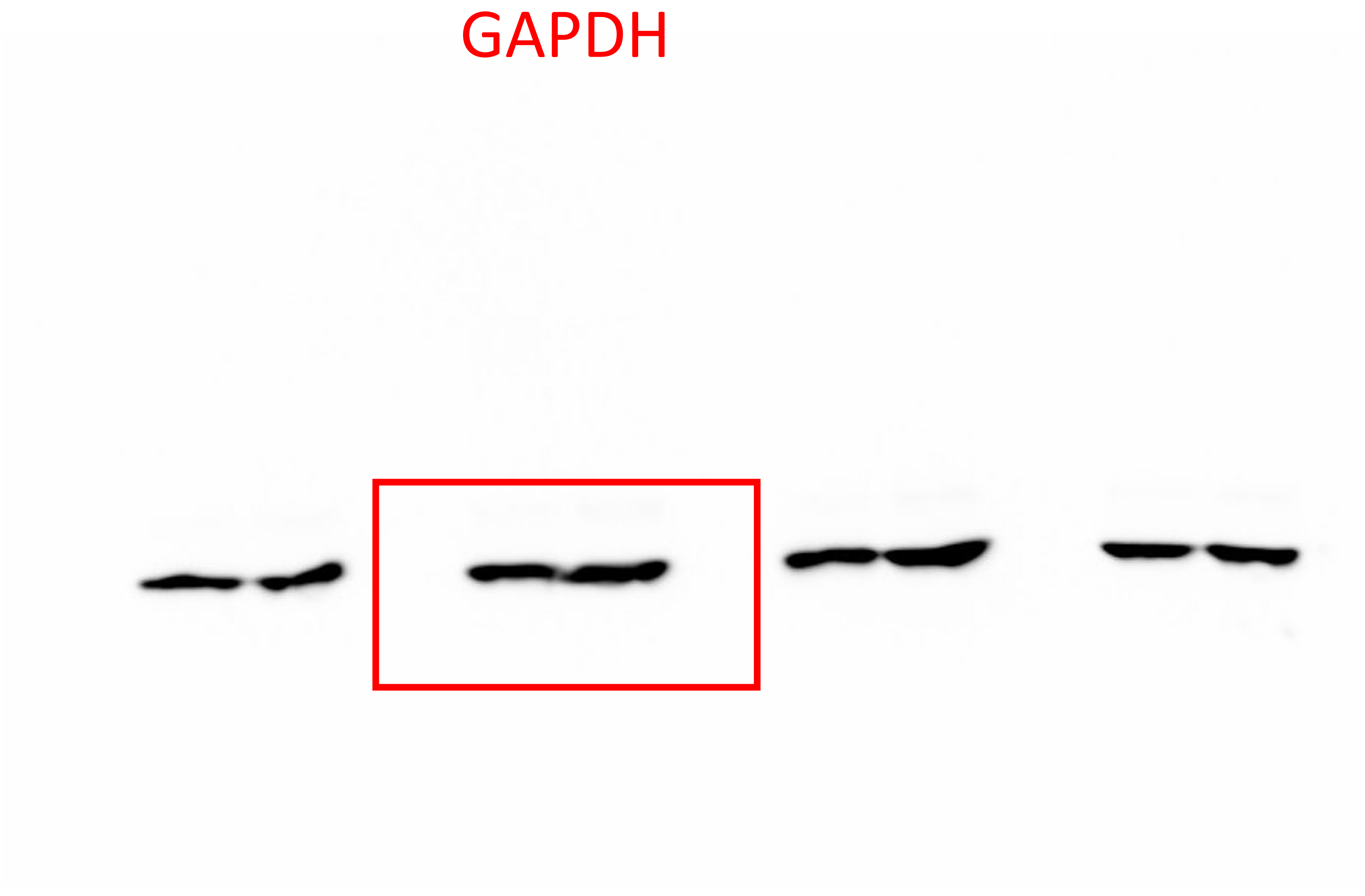

Full unedited gel for Figure 2D - M207

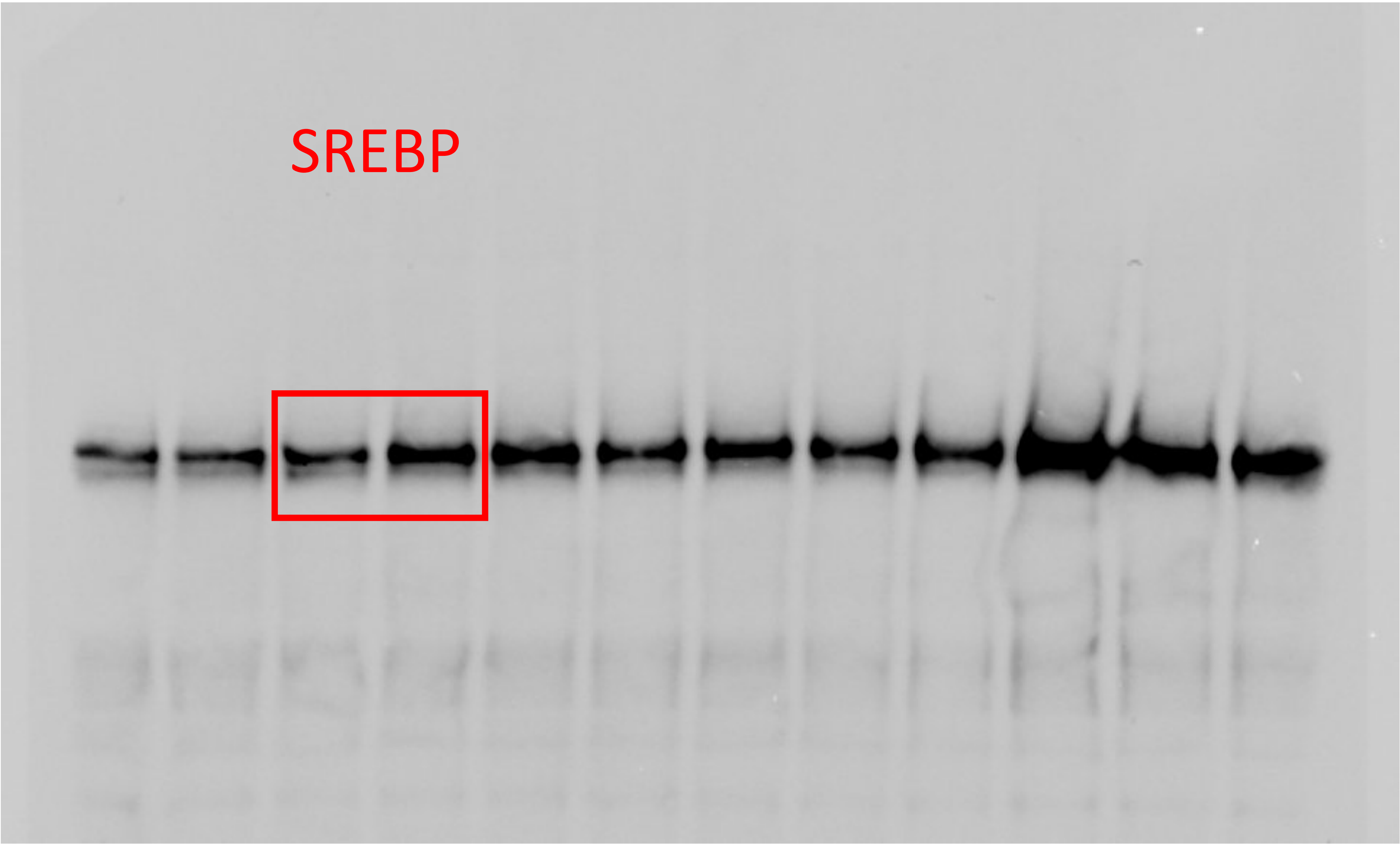

Full unedited gel for Figure 2D - M207

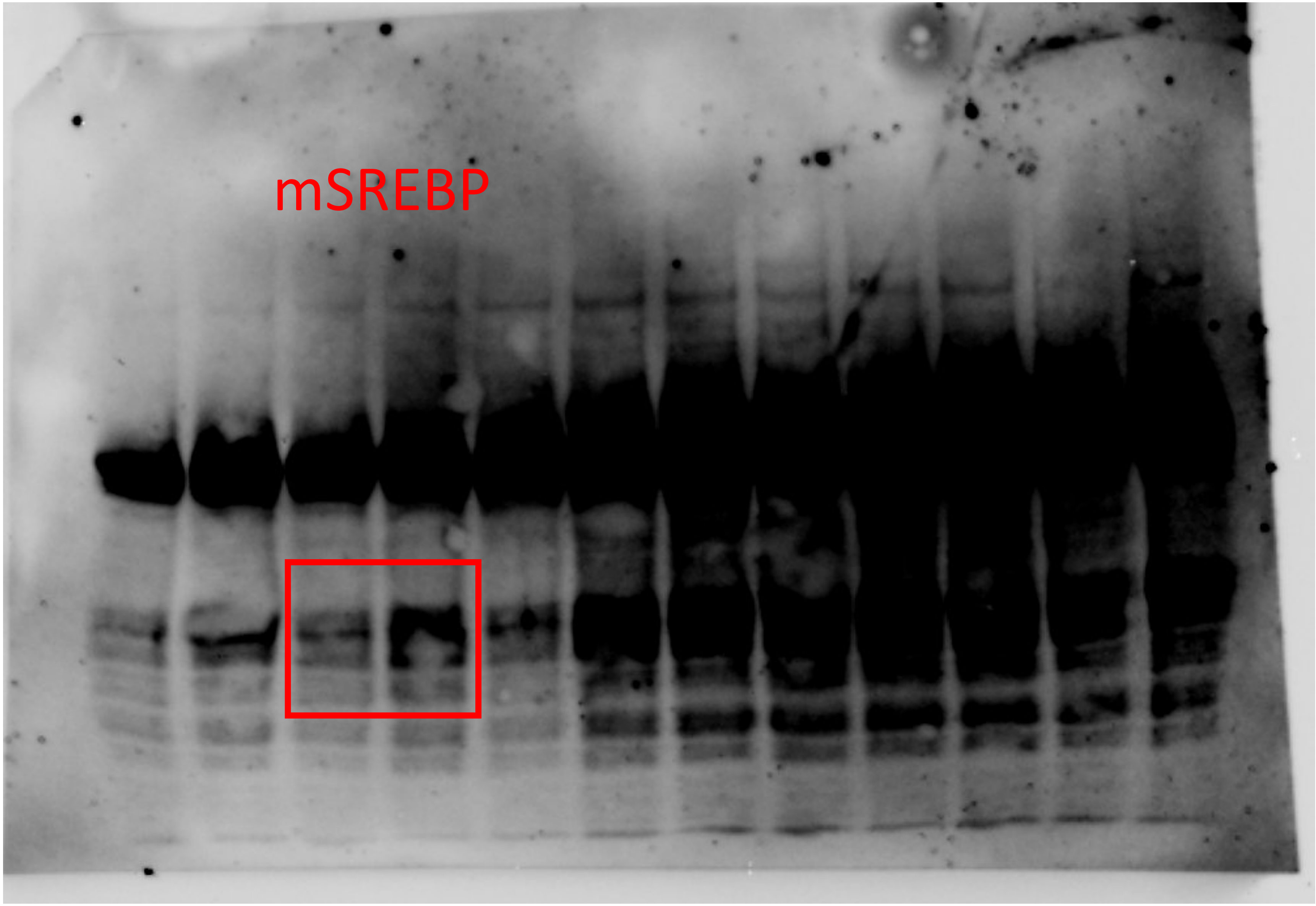

Full unedited gel for Figure 2D - M207

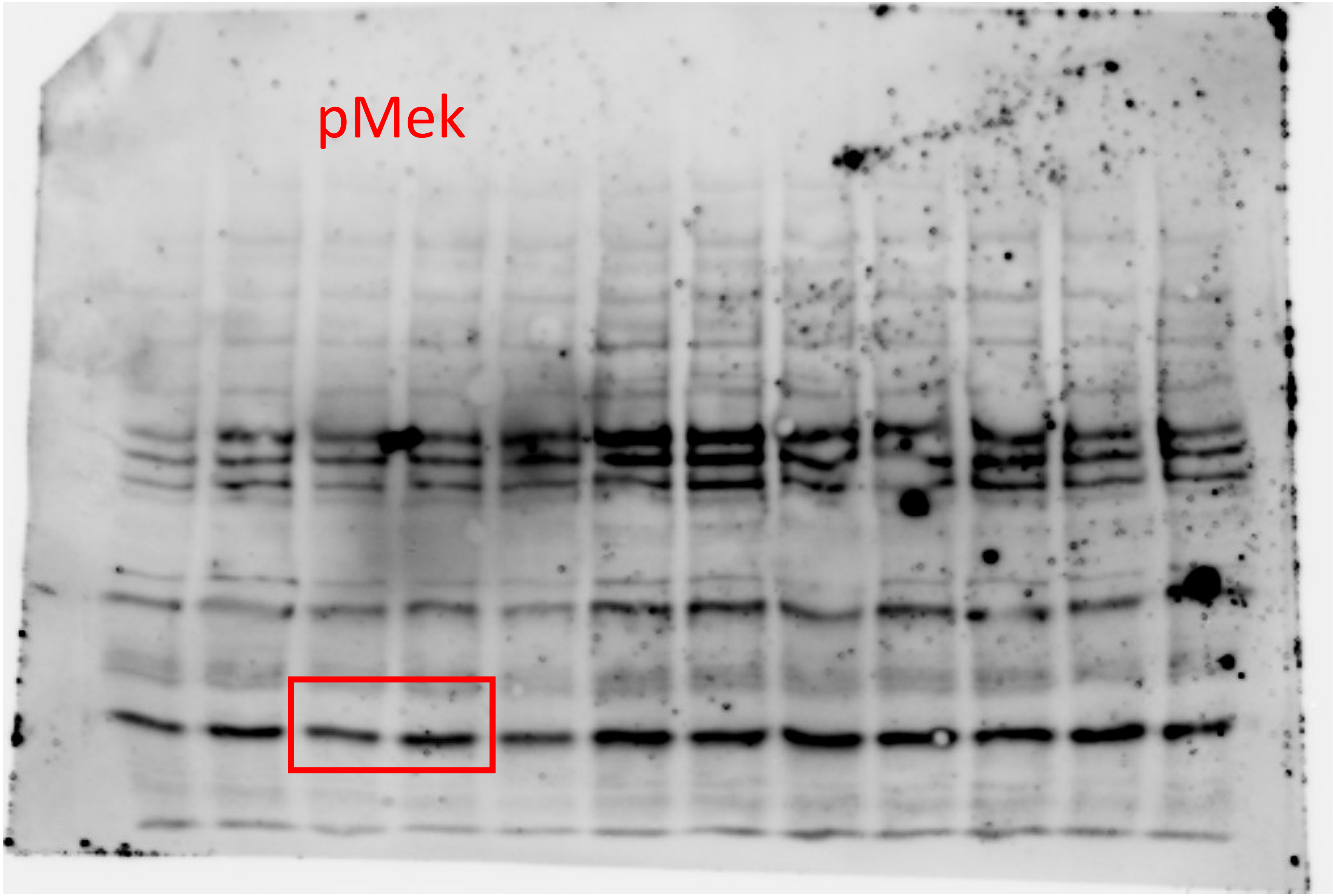

Full unedited gel for Figure 2D - M207

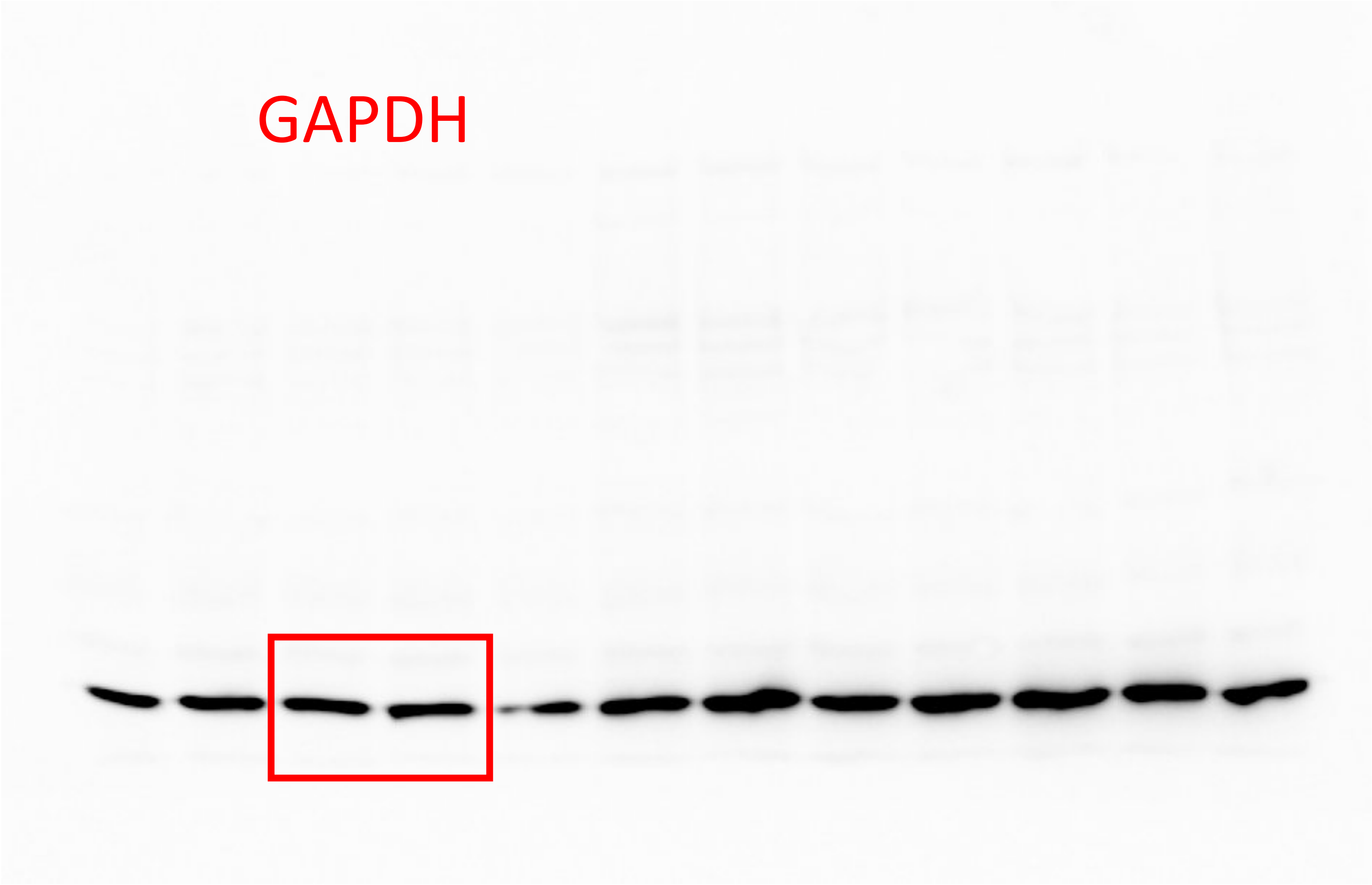

Supplement: Supplementary file 4 — Supplementary Data 1 [file 41467_2018_4664_MOESM4_ESM.pdf]
